# Genome-Wide Analysis of the Biosynthesis and Deactivation of Gibberellin-Dioxygenases Gene Family in *Camellia sinensis* (L.) O. Kuntze

**DOI:** 10.3390/genes8090235

**Published:** 2017-09-19

**Authors:** Cheng Pan, Kunhong Tian, Qiuyan Ban, Leigang Wang, Qilu Sun, Yan He, Yuanfei Yang, Yuting Pan, Yeyun Li, Jiayue Jiang, Changjun Jiang

**Affiliations:** 1 State Key Laboratory of Tea Plant Biology and Utilization, College of Tea and Food Technology, Anhui Agricultural University, Hefei 230036, China; panchengtt@163.com (C.P.); tiankh@ahau.edu.cn (K.T.); banqiuyan717@163.com (Q.B.); 15720340@ahau.edu.cn (L.W.); sunql@ahau.edu.cn (Q.S.); heyan@ahau.edu.cn (Y.H.); yangyf@ahau.edu.cn (Y.Y.); panyt@ahau.edu.cn (Y.P.); lyy@ahau.edu.cn (Y.L.); jiangjy@ahau.edu.cn (J.J.); 2 Henan Provincial Key Laboratory of Tea Plant Biology, College of Life Sciences, Xinyang Normal University, Xinyang 464000, China

**Keywords:** genome-wide analysis, gibberellin, Gibberellins-dioxygenases, abiotic stress, *Camellia sinensis*

## Abstract

Gibberellins (GAs), a class of diterpenoid phytohormones, play a key role in regulating diverse processes throughout the life cycle of plants. Bioactive GA levels are rapidly regulated by Gibberellin-dioxygenases (GAox), which are involved in the biosynthesis and deactivation of gibberellin. In this manuscript, a comprehensive genome-wide analysis was carried out to find all GAox in *Camellia sinensis*. For the first time in a tea plant, 14 *CsGAox* genes, containing two domains, DIOX_N (PF14226) and 2OG-FeII_Oxy, were identified (PF03171). These genes all belong to 2-oxoglutarate-dependent dioxygenases (2-ODD), including four *CsGA20ox* (EC: 1.14.11.12), three *CsGA3ox* (EC: 1.14.11.15), and seven *CsGA2ox* (EC: 1.14.11.13). According to the phylogenetic classification as in *Arabidopsis*, the *CsGAox* genes spanned five subgroups. Each *CsGAox* shows tissue-specific expression patterns, although these vary greatly. Some candidate genes, which may play an important role in response to external abiotic stresses, have been identified with regards to patterns, such as *CsGA20ox2*, *CsGA3ox2*, *CsGA3ox3*, *CsGA2ox1*, *CsGA2ox2*, and *CsGA2ox4*. The bioactive GA levels may be closely related to the *GA20ox*, *GA3ox* and *GA2ox* genes. In addition, the candidate genes could be used as marker genes for abiotic stress resistance breeding in tea plants.

## 1. Introduction

Gibberellins (GAs), a class of diterpenoid phytohormones, play a key role in regulating diverse processes throughout the life cycle of plants, such as stem elongation, anther development, and flower induction [1,2,3,4]. Although up to 136 different gibberellin molecules have hitherto been discovered, most of these molecules have been identified as biosynthetic intermediates or catabolites of bioactive GAs, with GA_1_, GA_3_, GA_4_, and GA_7_ being the only bioactive GAs [1,5]. Recent studies on GA biosynthesis and deactivation, as well as crosstalk of GA with other plant hormones and environmental cues have achieved great progress, along with advancements in molecular genetics and functional genomics [1,6].

**Figure 1:**
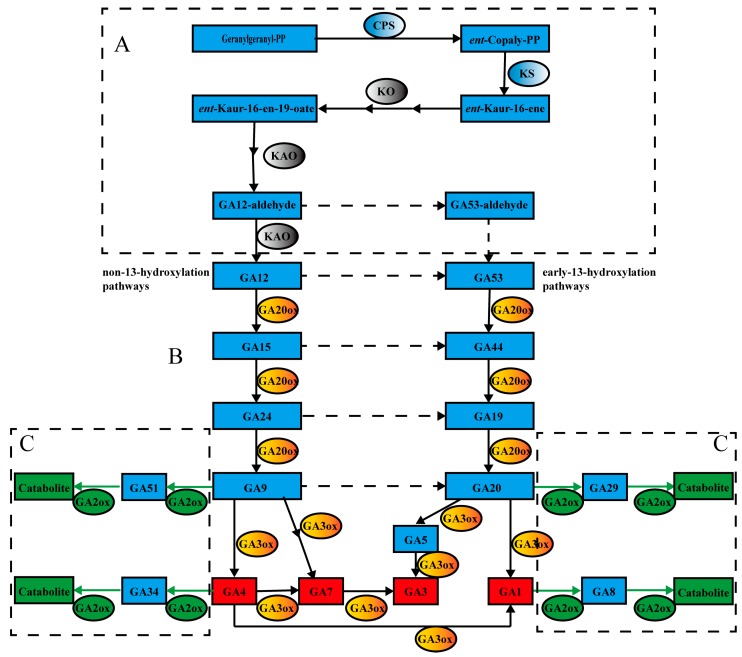
The principal pathways of gibberellin (GA) biosynthesis and deactivation GA-dioxygenases in higher plants. In this figure, Ggeranylgeranyl-PP = geranylgeranyl diphosphate; *ent*-Copaly-PP = *ent*-copalyl diphosphate; CPS = *ent*-copalyl diphosphate synthase; KS = *ent*-kaurene synthase; KAO = *ent*-kaurenoic acid oxidase; KO = *ent*-kaurene oxidase; GA20ox = GA 20-oxidase; GA3ox = GA 3-oxidase; GA2ox = GA 2-oxidase.

In higher plants, the biosynthesis and deactivation of GA mainly involves three stages of reactions (Figure 1) [1,3]. In the first stage, the synthesis of GAs begins from geranylgeranyl diphosphate (Geranylgeranyl-PP), which creates the metabolite *ent*-kaur-16-ene through two synthetases, *ent*-copalyl diphosphate synthase (CPS) and *ent*-kaurene synthase (KS). Following this, GA_12_ and GA_53_ are synthesized from *ent*-kaur-16-ene by cytochrome P450-dependent monoxygenases—these are *ent*-kaurene oxidase (KO) and *ent*-kaurenoic acid oxidase (KAO) respectively—through three consecutive steps (Figure 1A) [1,7]. In the final stage of biosynthesis, the synthesis of various GAs occurs via two pathways: the early-13-hydroxylation and the non-13-hydroxylation pathways (Figure 1) [6,8]. The 2-oxoglutarate dependent dioxygenases (2-ODDs), including GA 20-oxidases (GA20ox) and GA 3-oxidases (GA3ox), are the key enzymes in a series of oxidation steps, which convert GA_12_ and GA_53_ to various GA intermediates and bioactive GAs (GA_1_ and GA_4_) (Figure 1B). In some species, bioactives (GA_3_ and GA_7_) probably derive from GA_9_ and GA_20_ under the oxidation catalyzed by GA3ox enzymes, respectively, via 2,3-didehydro-GA_9_ and GA_5_ [1]. In the pathways and regulation of GA degradation, GA 2-oxidases (GA2ox) are the unique enzymes, which also belong to 2-ODDs. These enzymes inactivate the bioactives (GA_1_ and GA_4_) and their immediate precursors (GA_20_ and GA_9_) (Figure 1C) [1]. 

Interestingly, the enzymes involved in the first step in the degradation of bioactive GAs and the final step of GA biosynthesis were found to be similar (Figure 1B,C). Furthermore, previous analyses found that all the GA20ox, GA3ox and GA2ox sequences belonged to the 2-ODDs superfamily, which share high homology with the functional domains [9,10,11,12]. Most of the *GA20ox* genes of the biosynthesis and catabolism of gibberellin have been now cloned and identified [2,8]. GA20ox, identified as a multi-catalytic enzyme, may play a regulatory role in the biosynthetic flux of the pathway [12]. The putative sequence of GA20ox contains the proposed consensus sequence NXYPXCXXP and three histidine residues, which are involved in binding the common co-substrate 2-oxoglutarate and Fe^2+^, respectively [13]. The sequence LPWKET, which may be related to the binding of the GA substrate, is also conserved in the GA20oxes of pumpkin [14] and *Arabidopsis* [13]. In *Arabidopsis*, the first three *GA2ox* genes cloned were annotated as *AtGA2ox1*, *AtGA2ox2* and *AtGA2ox3* [15]. After this, the genes identified in Hedden and Phillips [6] were described as *AtGA2ox4* and *AtGA2ox6*. Furthermore, *AtGA2ox7* and *AtGA2ox8*, distinct from *AtGA2ox1*–*AtGA2ox6* [6], were noted by Schomburg et al. [11]. In summary, GA20ox, GA3ox, and GA2ox each encoded by a small gene family, with all *CsGAox* gene expression patterns showing tissue-specific and different responses to abiotic stresses [6]. Expression patterns of GA20ox and GA3ox could be involved in the negative feedback regulation of bioactive GAs [1,2], while GA2ox may be regulated by positive feedback regulation [1,16]. Many environmental responses are regulated through GA abundance, while GA metabolism is regulated by environmental signals as well as by abiotic and biotic stresses [1]. GA metabolism and signaling seem to be closely regulated through feedback mechanisms to maintain GA homeostasis [1,2].

Endogenous GA and abscisic acid (ABA) strongly influence the growth of tea plants [17,18,19]. However, many studies focus on physiology, with few having examined gene expression. Results of the *Camellia sinensis* var. *assamica* tea tree genome sequence, which provided the opportunity to perform a genome-wide scan of gene families, were published in 2017, while the genome database was opened publicly at the same time [20]. In this study, a genome-wide survey of the *Gibberellin-dioxygenase* (*GAox*) gene family was conducted using the Tea Tree (*C. sinensis* var. *assamica*) Genome Database [20]. Meanwhile, sequences were supplied from the tea plant (*C. sinensis*) transcriptome databases [21]. Predicted sequences of all tea *GAox* genes were identified with the tool SMART, Pfam 31.0, and the NCBI database. Markedly, 14 full-length *GAox* genes were confirmed for the first time in tea plants (*C. sinensis*), using cloning and sequencing. Some characteristics and putative protein sequences were predicted and identified. Moreover, to gain insights into the evolutionary diversity of *GAox* genes, a comparative phylogenetic analysis of the tea plant with the *Arabidopsis* and the other ten species plant *(Citrus sinensis*, *Cucumls sativus*, *Glycine max*, *Medicago truncatula*, *Physcomitrella patens*, *Populus trichocarpa*, *Selaginella moellendorffii*, *Sorghum bicolor*, *Vitis vinifera* and *Zea mays) GAox* gene family was performed. Tissue-specific expression and expression patterns of *CsGAox* genes in response to abiotic stresses, including high- or low-temperature stress, exogenous GA or ABA, polyethylene glycol (PEG), and high salinity treatments were analyzed. In tea plants, *CsGAoxes* has different patterns of response in tissues and abiotic stress. For future research, this study will serve as a foundation into the functional roles of *CsGAox* genes.

## 2. Materials and Methods

### 2.1. Plant Materials and Abiotic Treatments

The plant material we picked was the tea cultivar ‘Wuniuzao’ (*C. sinensis* cv. Wuniuzao). For tissue and abiotic stress treatments, two-year-old cutting seedlings were planted in a pot and grown with a natural photoperiod under greenhouse conditions at the State Key Laboratory of Tea Plant Biology and Utilization, Tea Science Research Institute, College of Tea and Food technology, Anhui Agricultural University (Hefei, China). We also provided thorough pest and fertilizer management [22].

For the GA or ABA treatment, a freshly prepared working solution of 100 μM GA or ABA was sprayed on the leaves. For high- or low-temperature treatment, tea plants grown in plant growth chambers (temperature 25 °C ± 2 °C) were transferred to chambers maintained at 38 °C or 4 °C for 24 h [23]. The second and/or third mature leaves were obtained at 0, 4, 12 and 24 h under the above treatment for gene expression analysis. To initiate drought stress, whole plants were removed from the pots, washed with tap water to remove the soil from the roots, and rinsed with purified water for 15 min. The plants were transferred to a 10% (*w/v*) PEG-6000 solution for 24 h [24,25]. Finally, stress was alleviated by washing the plants with tap water to remove the PEG-6000, with the plants recovered without PEG for another 48 h. The second and/or third mature leaves were obtained at 0, 4, 8, 12, 24 h as well as and recovery at 48 h under drought stress treatment for gene expression analysis. To initiate salt stress, two-year-old plants were irrigated with 100 mM NaCl for 24 h [24,25]. The second and/or third mature leaves were obtained at 0, 4, 8, 12, 24, 48, 72 and 120 h under salt stress treatment for gene expression analysis. Controls (non stress-treated plants) were set up at each time point, and for all the above assays, plants of approximately equal size were selected for all treatments. The three independent biological replicates of each varieties were harvested. The samples were harvested, quickly frozen in liquid nitrogen, and stored at −80 °C for RNA extraction.

### 2.2. RNA Isolation and cDNA Synthesis

The sample was ground into powder with liquid nitrogen, before the total RNA was isolated in accordance with the method of a commercial RNAprep pure plant kit (QIAGEN Co., Dusseldorf, Germany). A Nanodrop 2000 spectrophotometer (Thermo Scientific, Wilmington, DE, USA) was used to measure the concentration of isolated RNA, and the quality were assessed using 1.2% formaldehyde–agarose gel electrophoresis. For quantitative reverse transcriptase polymerase chain reaction (qRT-PCR), the first-strand copy DNA (cDNA) of the sample was synthesized with the PrimeScript™ II 1st Strand cDNA Synthesis Kit (TaKaRa, Tokyo, Japan). The cDNA was diluted 10-fold for PCR amplification. 

### 2.3. Data Mining for GAox Protein Genes 

A multiple database search was performed to find all of the members of the GAox family in *Sorghum bicolor*, *Physcomitrella patens*, *Populus trichocarpa*, *Cucumls sativus*, *Medicago truncatula*, *G.max*, *Citrus sinensis*, *Vitis vinifera*, *Zea mays*, *Selaginella moellendorffii* and *Arabidopsis* [26]. The strategy to obtain every gene of the GAox family in an *Arabidopsis* genome involved the following steps.

The *A. thaliana* GAox proteins were retrieved from the The *Arabidopsis* Information Resource (TAIR) [27] database. For GA-dioxygenase protein genes in *Arabidopsis* (AtGAox), the key word “gibberellin” was first used as a query to search in the TAIR database [28,29]. Following this, the sequences obtained were used as queries to search in the TAIR10 Protein databases using BioEdit software [30], with the BLASTP program at the e-value of 10^−3^ to search all similar sequences [31]. All sequences obtained were then used as queries to search against the TAIR Protein databases using TAIR BLAST 2.2.8 [27] to avoid false positives [32]. After this, we verified these sequences using the tool SMART [33], the Pfam 31.0 database [34], and the NCBI database [35].

For GA-dioxygenase protein genes in tea plant (*CsGAox*), all predicted GAox protein sequences of multiple databases were used as query sequences to search against the Tea Tree Genome Database (Available online: http://www.plantkingdomgdb.com/tea_tree/) [20] using the BLASTP program. The e-value used in the BLASTP was 10^−3^. The tool SMART, Pfam 31.0, and NCBI database were finally used to confirm each predicted *CsGAox* protein sequence as the GAox protein family.

### 2.4. Cloning the Full-Length of cDNA of GAox Protein Genes

All predicted CsGAox protein sequences were used as query sequences to search in transcriptome databases (previously generated from tea plant cultivar ‘Shuchazao’) [21] using TBLASTN program. All the target unigenes were identified by BLASTx (NCBI), and sequences with more than 60% matching identity were used for assembly using SeqMan software (DNAStar, Inc. Madison, WI, USA) in the DNAStar package. Finally, all predicted protein of assembled sequences were compared with known GAox sequences by applying ClustalX [36] to verify that the sequences were candidates. PCR primer (Supplementary Table S1) pairs were designed, using the software Primer Premier 5 (Premier Biosoft International, Palo Alto, CA, USA), to clone the full-length cDNA sequences of the *CsGAox* genes by RT-PCR. The PCR product was purified with an Axygen Gel Extraction Kit (Axygen, Union City, CA, USA), and was ligated into a pMD-19T vector (TaKaRa, Tokyo, Japan), from which single clones were sequenced by Sangon Biotech Co. (Shanghai, China). The sequence accuracy was confirmed by sanger dideoxy sequencing.

### 2.5. Analysis of Sequences

Information about the length of coding sequences and amino acid sequenced was determined by DNAMAN and DNAstar software. The molecular weights and theoretical pIs were predicted using the ProtParam [37] tool. WoLF PSORT [38] and TargetP [39] were combined to predict the subcellular localization of the proteins. Predicted amino acid sequence alignment and phylogenetic tree of the *CsGAoxes* with the GAoxes from other plant species were aligned using the ClustalW method with default parameters, in Bioedit and MEGA6.0 [30,36,40]. To determine the subgroup classification of *CsGAox* genes, the phylogenetic tree and conserved motifs were assessed for the *CsGAox*-encoded proteins. GAox proteins from *Arabidopsis* and ten plant species (*Citrus sinensis*, *Cucumls sativus*, *G. max*, *M. truncatula*, *P. patens*, *P. trichocarpa*, *S. moellendorffii*, *S. bicolor*, *V. vinifera*, and *Z. mays*) GAox proteins were used to classify the tea plant proteins into different groups using the neighbor-joining method with 1500 bootstrap replicates in MEGA6.0. The full-length amino acid sequences of *CsGAox* genes were entered into the MEME analysis tool to find their conserved motifs with the maximum number of motifs to identify set to 15 [41]. Each de novo detected motif was further subjected to a search in the Interpro database [42] to find any resemblance with known domains. Consensus sequences were also separately scanned in the Interpro database to find the domains present in pre-identified *GAoxes*.

### 2.6. Gene Expression Analysis by qRT-PCR

Specific primers (Supplementary Table S2) for qRT-PCR were designed using the software Primer Premier 5, and synthesized by Sangon Biotech Co. (Shanghai, China). The primer sequences with the cDNA template were checked by PCR. To ensure the efficiency of optimal polymerization, the amplification length for each gene was restricted to 150–300 bp. The reaction program of qRT-PCR was performed under the following conditions: 95 °C for 30 s, followed by 40 cycles at 95 °C for 5 s, and 60 °C for 30 s. The reaction volume was 25 μL, which contained 4 μL of diluted cDNA strand, 6.5 μL of deionized water, 12.5 μL of SYBR^®^Premix Ex Taq™II (Tli RNaseH Plus; TaKaRa), 1 μL of forward primer, and 1 μL of reverse primer. The mean values and standard deviation were calculated based on three independent biological replicates. *CsActin* [43,44] was used as a reference gene to normalize the expression of related genes involved in GAs biosynthesis and recycling of tea plants.

The relative gene expression levels were calculated using the comparative 2^−ΔΔCt^ method [45]. Regarding the heatmaps, the 2^−ΔΔCt^ values of the transcripts of the *CsGAox* genes from various tea plant tissues samples were normalized to bud tissues and expressed as a log2-fold change. In the stress-treated plant samples, the values were normalized to plant samples of the 0 h treatment and expressed as a log_2_-fold change. Concerning the histogram of qRT-PCR analysis, the relative gene expression levels were calculated and expressed as the fold change relative to expression of the 0 h treatment (expression = 1). Differences in gene expression levels were detected by Tukey’s multiple-range test at a 0.05 probability level. The qPCR experiments were conducted with three independent total RNA samples. 

## 3. Results

### 3.1. Isolation, Identification and Annotation Information of the GAox Family Genes in Tea Plants

Based on genome and transcriptome databases, more than 60 candidate *GAox* genes were obtained in the tea plant. Several sequences, which shared approximately 99% open reading frame (ORF) identity with other candidate *CsGAox* genes (data not shown), were excluded, and 14 genes were further analyzed. Afterwards, we named genes based on the nomenclature and the name of *Arabidopsis*. The gene names, accession numbers, Gene ID, number of deduced amino acid, molecular weights, predicted subcellular localizations and groups classifications are summarized in Table 1. *CsGA20ox1*, *CsGA3ox1* and *CsGA3ox2* isoforms have been submitted to NCBI (Direct submission). The table shows that the identified GAox family genes encode proteins ranging from 332 (GA2ox2) to 383 (GA20ox1) amino acids (aa) in length, with an average of 353 aa. There was a predicted molecular mass range of 37.18–43.03 kDa, and a pI ranging from 5.30 (CsGA20ox4) to 8.22 (CsGA3ox2). Most *CsGAox* family genes were predicted to be located in the nucleus and cytoplasm, which is related to their functional location [1,2,3,6,46]. 

### 3.2. Similarity and Phylogenetic Gene Structure Analysis of the GAox Genes

**Figure 2:**
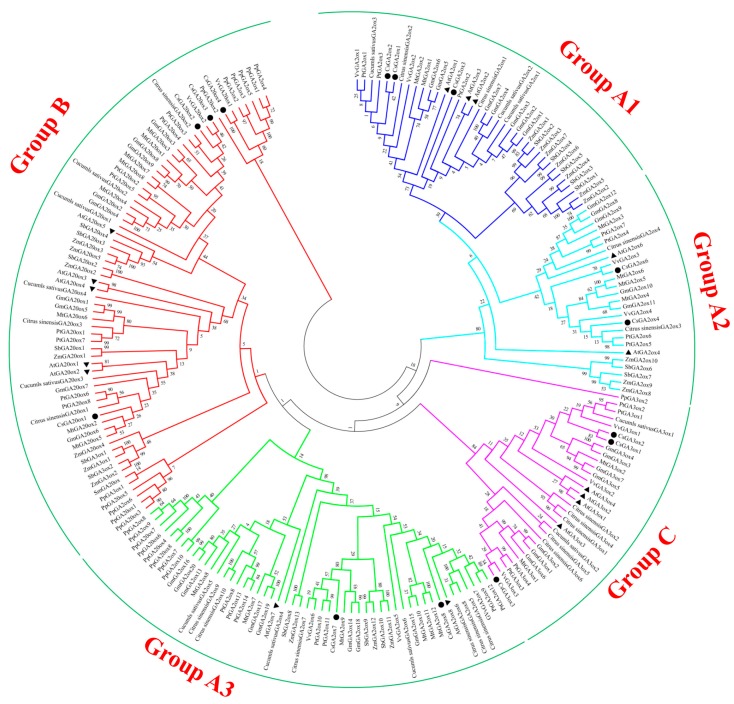
Phylogenetic analysis of putative GAox family proteins in *C. sinensis* with other plant GAoxes. The following species were analyzed: *Arabidopsis*, *C. sinensis*, *Citrus sinensis*, *Cucumls sativus*, *G.max*, *M. truncatula*, *P. patens*, *P. trichocarpa*, *S. moellendorffii*, *S. bicolor*, *V. vinifera*, and *Z. mays*. The predicted amino acid sequences of the 14 CsGAoxs and their corresponding sequences from *Arabidopsis* and from 10 other plant species were aligned using the ClustalW [36] sequence alignment program. The phylogenetic tree was constructed using MEGA6.0 [40] software with the neighbor-joining tree method with 1500 bootstrap replicates. Five subgroups are shown in various colors. The sequences of *C. sinensis* and *Arabidopsis* are highlighted with black dots and triangles, respectively.

In order to investigate evolutionary relationships, a total of 213 GAox protein sequences from *C. sinensis*, *Arabidopsis* and other plant species were subjected to phylogenetic analysis. All sequences derived from dicotyledonous plants are well divided, but for monocotyledon plants, some sequences (SbGA3ox1, SbGA3ox2, ZmGA3ox1 and ZmGA3ox2) were not accurately classified. However, most sequences of *P. patens* were not well divided following the same distinction. The results indicated that all the *CsGAox* sequences belonged to the 2-ODDs superfamily. From a functional point of view, the *CsGAox* genes were classified into three subfamilies: the biosynthesis of gibberellin gene family containing the *GA20ox* and *GA3ox* genes encoding GA20ox protein (EC: 1.14.11.12) and GA3ox protein (EC: 1.14.11.15). Furthermore, the most studied deactivation pathway is 2β-hydroxylation, which is catalyzed by a class of GA2ox enzymes (EC: 1.14.11.13). These are soluble 2-ODDs [1,2,3,6,7,15,46,47,48,49]. Particularly, *GA2ox* genes were classified into three subgroups. Therefore, *CsGAox* genes were classified into three final subfamilies and five different subgroups (Figure 2 and Table 1). 

Predicted amino acid sequence alignment and phylogenetic tree of the *CsGAoxes* with the GAoxes from other plant species were aligned using the ClustalW method. Results of their similarities and differences were listed in Supplementary Figure S1 (group B), Figure S2 (group C), Figure S3 (group A1), Figure S4 (group A2) and Figure S5 (group A3). Except *CsGA2ox7*, most of the *CsGA3ox* (group C) and *CsGA2ox* (group A) genes showed that the phylogenetic relationships of *CsGAoxes* were closer to edicots compared to monocots, indicating that the evolutionary rate of *CsGAox* gene family was faster than previously thought. With regards to *GA20ox* genes (group B), *CsGA20ox1*, *CsGA20ox2*, and *CsGA20ox3* showed a closer relationship with edicots. *CsGA20ox4* and *VvGA20ox1* however, which were clustered together in the group of *Physcomitrella patens*, showed notable differences from the *GA20ox* genes of edicot and monocot. The variant phylogenetic relationships of CsGAoxes indicated that the *GAox* genes had different evolutionary rates during tea plant evolution. Using a multiple sequence alignments program to analyze the 14 ORFs and their encoded amino acid sequences, the results of their similarities were listed in Supplementary Table S3. The nucleotide sequence similarities varied from 38.9% (*CsGA3ox2* and *CsGA2ox8*) to 87.0% (*CsGA2ox2* and *CsGA2ox1*), while proteins exhibited similarities from 22.3% (*CsGA2ox3* and *CsGA2ox7*) to 86.1% (*CsGA2ox1* and *CsGA2ox2*).

**Figure 3:**
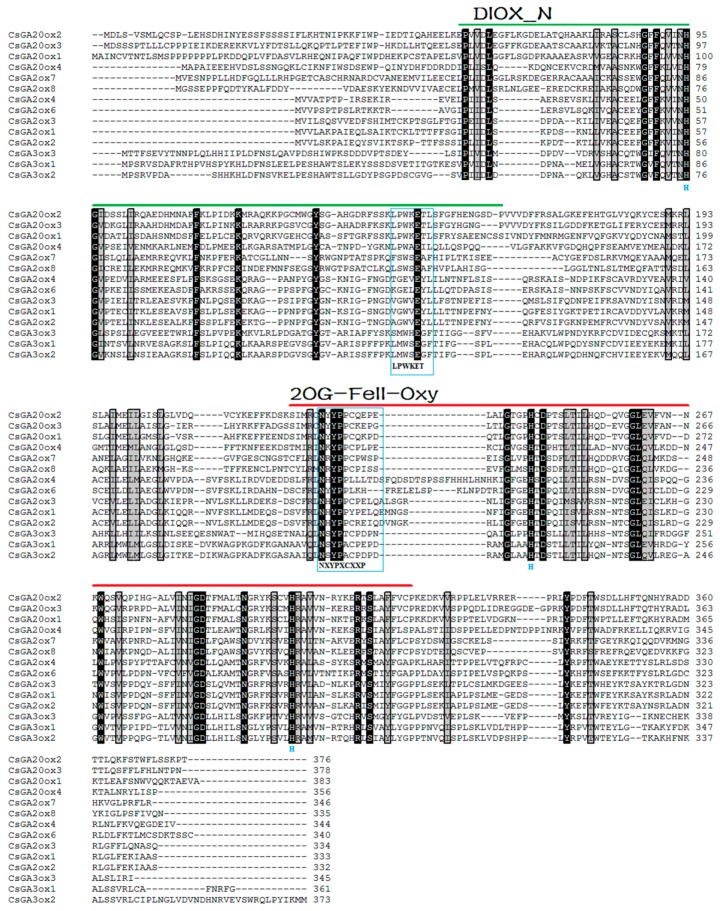
Alignment of the predicted amino acid sequences for the CsGAoxs. Putative tea plant GAox amino acid sequences were aligned using ClustalW [36] method and were edited in Bioedit [30]. Identical and similar residues are shaded in black and gray, respectively. The DIOX_N (PF14226) and 2OG-FeII_Oxy (PF03171) domains are highlighted with a bold line. The three histidine residues for binding Fe^2+^ are all conserved, which are marked under the alignment. The proposed consensus sequence NXYPXCXXP and LPWKET are highlighted by boxes.

The alignment in Figure 3 shows that these sequences contained the two domains DIOX_N (PF14226) and 2OG-FeII_Oxy (PF03171). GA20ox, GA3ox and GA2ox are members of the 2-ODDs. The proposed consensus sequence NXYPXCXXP of 2-ODDs, which may be involved in the binding of a common cosubstrate (2-oxoglutaric acid) [13,50], was highly conserved in all CsGA20ox and CsGA3ox (Supplementary Figures S1 and S2), but only a partial (NXYPXC) sequence was highly conserved in CsGA2ox (Supplementary Figures S3–S5). Meanwhile, the three histidine residues for binding Fe^2+^ were all conserved in all putative CsGAoxes [13,50]. The sequence LPWKET, which is highly conserved in all GA20ox so far (Supplementary Figure S1) has never been certified to exist other 2-ODDs [1,9,11], and may be involved in the binding of the GA substrate [13]. The related enzymes, GA3ox and GA2ox, did not contain this proposed consensus sequence (Supplementary Figures S2–S5) [13]. The proposed consensus sequence of CsGA3ox may be the sequence MWSEGXT (Figure 3 and Supplementary Figure S2). However, the sequence of CsGA2ox may be classified into three subgroups (XGWVEYLL, XGEXEYLL and XSWSEAXH). Lee and Zeevaart [51] suggest that *GA2ox* family can be divided into three classes on the basis of the phylogenetic relationships. The *CsGAox* genes were also classified into three subfamilies and five different subgroups based on this partial sequence. These individual genes play specific roles in different developmental processes that are regulated by GA. The diversified sequence may be related to the binding of the GA substrate in all *GAoxes* and thus, more studies are needed to clarify this relation.

**Figure 4:**
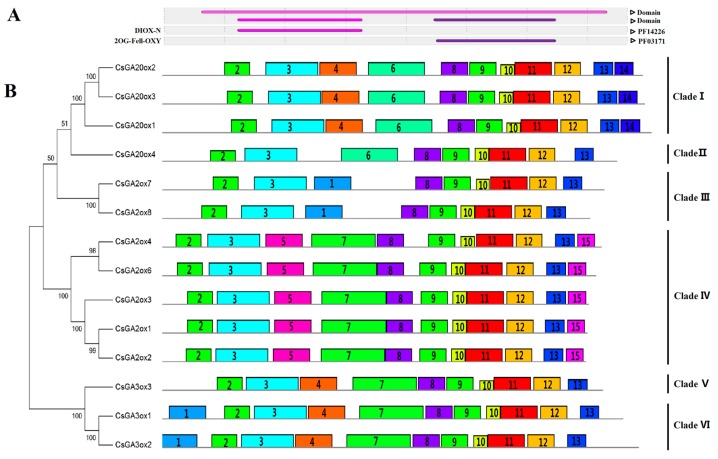
Phylogenetic analysis and conserved motif identification in CsGAox proteins from tea plants. (**A**) Interpro analysis revealed two types of domains as a part of the 2-oxoglutarate-dependent dioxygenase superfamily in previously defined GAox proteins; (**B**) De novo motif identification of GAox proteins. Motifs 2 and 3 show resemblance to the DIOX_N (PF14226) domain, while motifs 8–12 show resemblance to the 2OG-FeII_Oxy (PF03171) domain. Finally, motifs 4–7 may be involved in the binding of the GA substrate.

A phylogenetic tree was constructed and 15 discrete motifs were found, with 14 CsGAox proteins used to further assess and classify the CsGAox proteins (Figure 4). The CsGAox proteins could be classified into six clades when analyzed together with the motifs. Due to the lack of Motifs 4 and 14, CsGA20ox4 was separated from other members of GA20ox into Clade II. GA3ox3 was classified to Clade V, due to the lack of Motif 1. Motifs 2 and 3 may be contained in the DIOX_N (PF14226) domain, while the 2OG-FeII_Oxy (PF03171) domain may consist of the motifs 8–12. Motifs 4–7 were highly diversified in all CsGAox proteins. These motifs may be involved in the binding of the GA substrate in all *GAoxes* and thus, more studies are needed to clarify this relation. The results of the MEME prediction may suggest evolutionary conservation in the basal architecture of *GAox* family members. 

### 3.3. Tissue-Specific Expression of CsGAoxes

**Figure 5:**
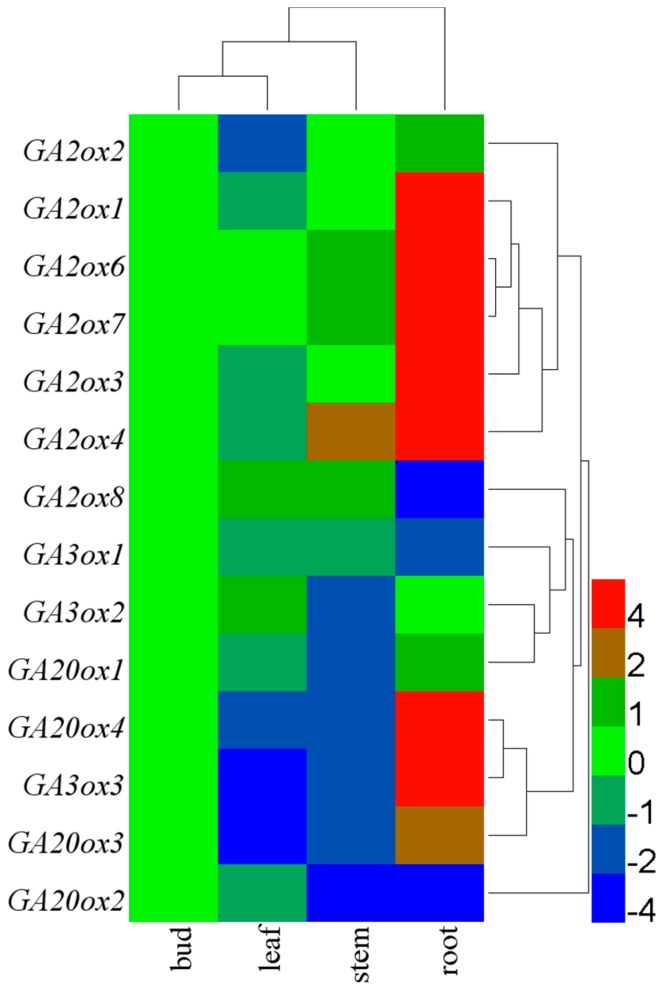
Tissue-specific expression of *CsGAoxs*. The relative gene expression levels were calculated using the 2^−ΔΔCt^ method and expressed as the fold change relative to expression of the bud tissue. Actin was used as housekeeping gene. The mean expression values were again normalized with logarithm with the base of 2 using the HemI [52] software. The color bar in all heat maps represents the expression values: blue represents low expression, green represents no significant difference in expression, and red denotes high expression.

Gibberellins play a key role in regulating diverse processes throughout the life cycle of plants [1,2,3,4]. The tissue specificity of *GAox* gene expression may be related to the physiological and biochemical functions of each tissue. Hence, it is important that the *CsGAox* gene expression in specialized tissue should be documented and analyzed. The transcript abundance of 14 *CsGAoxes* in four different tissues, including roots, stems, leaves and bud, was obtained. Variable transcription levels showed that these genes were ubiquitously and specially expressed in all tissues (Figure 5 and Supplementary Figure S6). *CsGAox* genes, such as *CsGA2ox1*, *CsGA2ox3*, *CsGA2ox4*, *CsGA2ox6*, *CsGA2ox7*, *CsGA3ox3*, *CsGA20ox3*, and *CsGA20ox4* showed maximum expression levels in the root tissues, while some other *CsGAox* genes, such as *CsGA2ox8*, *CsGA3ox1*, and *CsGA20ox2* showed several-fold lower expression in root tissues compared to the other three tissues. The similar expression levels of all *CsGAox* genes have been showed in leaf and stem tissues with few exceptions. Overall, each *CsGAox* shows tissue-specific expression patterns [6], although these patterns varied greatly.

### 3.4. Differential Expression of CsGAox Genes under Abiotic Stresses

**Figure 6:**
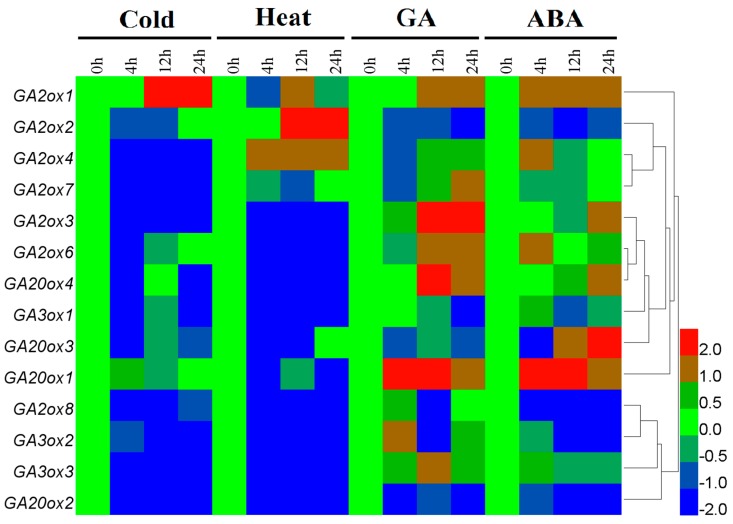
Effect of cold, heat, exogenous GA and ABA stresses on *CsGAox* gene expression in leaves of tea plants. The relative gene expression levels were calculated using the 2^−ΔΔCt^ method and expressed as the fold change relative to expression of the 0-h treatment. Actin was used as housekeeping gene. The mean expression values were again normalized using logarithm with the base of 2 using the HemI software [52]. The color bar in all heat maps represents the expression values: blue represents low expression, green represents no significant difference in expression, and red denotes high expression.

**Figure 7:**
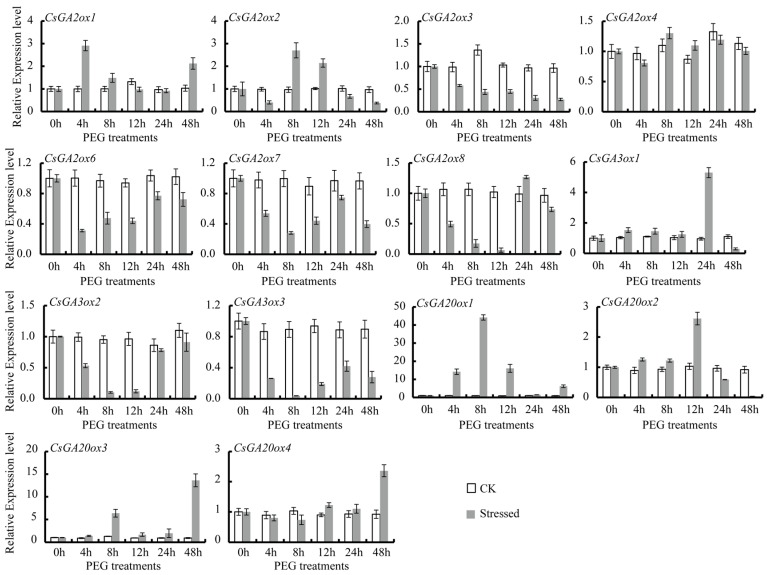
Effect of drought stresses on the *CsGAox* gene expression in leaves of tea plants. CK represents controls (non stress-treated plants) and stressed represents polyethylene glycol (PEG) stress-treated plants. The relative gene expression levels were calculated using the 2^−ΔΔCt^ method and expressed as the fold change relative to expression of the 0-h treatment. Actin was used as a housekeeping gene.

**Figure 8:**
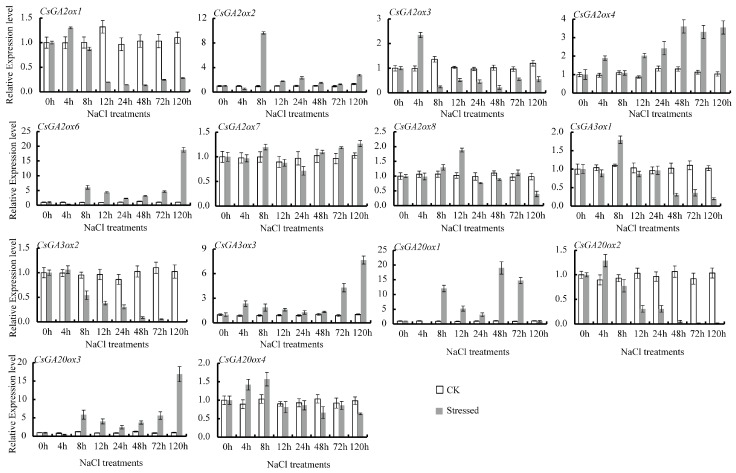
Effect of high salinity stresses on *CsGAox* gene expression in leaves of tea plants. CK represents controls (non stress-treated plants) and stressed represents NaCl stress-treated plants. The relative gene expression levels were calculated using the 2^−ΔΔCt^ method and expressed as the fold change relative to expression of the 0-h treatment. Actin was used as a housekeeping gene.

In tea plants, *CsGAoxes* take part in numerous abiotic stress responses. qRT-PCR was used to analyze the expression patterns of *CsGAox* genes in tea plants subjected to various abiotic stresses, such as cold (4 °C), heat (38 °C), ABA (100 μM), GA (100 μM), drought, and salinity (100 mM NaCl). Differential expression levels of *CsGAox* genes were observed under various stress conditions (Figure 6, Figure 7 and Figure 8). 

In the present study, the expression profile of *CsGAoxes* showed that the tea plants were markedly affected by conditions of short-term stress (0, 4, 12, and 24 h). As shown in Figure 6, several *CsGAox* genes were downregulated in leaves after cold and heat treatments. Notably, *CsGA2ox3*, *CsGA2ox8*, *CsGA3ox2*, *CsGA3ox3*, and *CsGA20ox2* showed several-fold lower expression after temperature stress. In contrast, another *CsGAox* gene (*CsGA2ox1*) was induced after 12 h of cold stress, while *CsGA2ox2* and *CsGA2ox4* were induced after 12 h of heat stress. Overall, CsGAox exhibited highly sensitive upregulation or downregulation of gene expression in response to temperature stress compared to controls (Figure 6, Supplementary Figures S7 and S8).

The susceptibility and resistance of plants to diseases can be increased with exogenous application of GA3 and an inhibitor of GA synthesis (uniconazole), respectively [53]. Endogenous GA and ABA strongly influence tea plant growth. The antagonistic roles played by GA and ABA regulate numerous developmental processes [54,55]. Under exogenous GA or ABA, *CsGAoxes* expression profiles are complicated. The expression pattern in response to GA or ABA of many genes were remarkably upregulated during 24 h of stress. The highest levels of *CsGA2ox3* and *CsGA20ox4* were observed after 12 h of GA treatment, while *CsGA20ox1* was dramatically induced after 4 h of GA or ABA stress. Meanwhile, GA or ABA stress suppressed the transcription of certain *CsGAoxes*, such as *CsGA2ox2*, *CsGA2ox8*, *CsGA3ox2* and *CsGA20ox2*. In general, seven genes containing *CsGA2ox3*, *CsGA2ox8*, *CsGA3ox2* and *CsGA20ox1-4* were upregulated or downregulated by at least four-fold after GA or ABA stress (Figure 6, Supplementary Figures S9 and S10). Similar conclusions have been obtained from previous studies. Most of GA2oxes are upregulated by GA treatment [15,16]. While, most of *GA20ox* and *GA3ox* genes are downregulated by applying GA [6,49]. The GA metabolism may be influenced by a feedback mechanism regulating bioactive GAs [6,56,57].

Under PEG treatment to leaves, almost all *CsGAoxes* had diminished expression levels during a 24-h treatment period. Notably, *CsGA2ox3*, *CsGA2ox6*, *CsGA2ox7*, *CsGA2ox8*, *CsGA3ox2*, and *CsGA3ox3* showed several fold lower expression after PEG stress (Figure 7). However, the expression patterns of certain genes varied greatly. For example, the expression of *CsGA2ox1*, *CsGA3ox1*, *CsGA20ox1* and *CsGA20ox3* was significantly induced at different points in time. In particular, *CsGA20ox1* was upregulated by at least 10-fold after PEG stress, and maintained relatively high levels after 48-h rehydration (Figure 7). In comparison, the other twelve genes, excluding the constitutively expressed *CsGA2ox3* and *CsGA3ox1* genes, displayed no significant changes in response to PEG after 24 h of treatment.

Upon NaCl stress to leaves, there were different responses from the *CsGAoxes* (Figure 8). The transcription level of four genes (*CsGA2ox2*, *CsGA2ox6*, *CsGA20ox1*, *CsGA20ox3*) was downregulated by high salinity stresses after 4 h, but increased significantly (at least five-fold) after this time point and maintained relatively high levels until 120 h (Figure 8). However, *CsGA2ox1*, *CsGA2ox3*, *CsGA2ox8*, *CsGA3ox1*, *CsGA3ox2*, *CsGA20ox2* and *CsGA20ox4* had diminished expression levels. In particular, four genes (*CsGA2ox1*, *CsGA2ox3*, *CsGA3ox2* and *CsGA20ox2*) were downregulated by at least four-fold after 8 h high salinity treatment (Figure 8). *CsGA3ox3* expression rapidly increased by at least four-fold at 72 and 120 h (Figure 8). 

## 4. Discussion

GAs play a key role in regulating diverse processes throughout the life cycle of plants, such as stem elongation, anther development, and flower induction [1,2,3,4]. GA metabolism may be influenced by a feedback mechanism that regulates bioactive GAs [6,56,57]. Recently, several plant genomes have been sequenced with the development of next-generation sequencing. As a result, the tea tree genome sequencing was published in 2017 [20], and the genome database fortunately opened publicly at the same time, which created opportunities for us to identify 14 full-length *CsGAox* protein-coding genes with genome-wide analysis in this study. We observed that these *CsGAoxes* can be classified into three final subfamilies and five different subgroups, including the groups A1, A2, A3, B and C. Most CsGAox family proteins were predicted to be located in the nucleus and cytoplasmic. Huang et al. [46] found that the OsGA2ox6-GFP protein had the same localization as the control GFP construct, giving fluorescent signals in both the nucleus and cytoplasm. Perhaps, there was no correlation between function and location. However, further research is also warranted for *CsGAoxes* in order to improve and perfect the tea plant genome database, such as chromosomal localization and the exon–intron organization of the corresponding *CsGAox* genes [58,59].

The GAox protein sequences from *C. sinensis*, Arabidopsis and *G. max* were subjected to phylogenetic analysis. The results indicated that these sequences contained the two domains of DIOX_N (PF14226) and 2OG-FeII_Oxy (PF03171), which belong to the 2-ODDs superfamily (Figure 3). Meanwhile, all *CsGAox* enzymes have been involved in the principal pathways of GA biosynthesis (group B and C) and deactivation (group A1, A2 and A3) in higher plants [1].

For group B and C, *GA20ox* and *GA3ox* converted GA_12_ and GA_53_ into various GA intermediates and bioactive GAs through two parallel pathways in the third stage of GA biosynthesis. Both *GA20ox* and *GA3ox* are soluble 2-ODDs that are present in the cytosol [13,60,61,62,63]. The two parallel pathways are: the non-13-hydroxylation branch, which leads to the production of 13-H GAs; including GA_4_, and the 13-hydroxylation branch, which leads to the biosynthesis of 13-OH GAs, including GA_1_ (Figure 1B) [1]. In group B, the *GA20ox* enzymes (EC: 1.14.11.12) are encoded by multigene families that are responsible for the production of GA_9_ and GA_20_, which are precursors of bioactive GAs (Figure 1B) [1,6,14,64,65]. To date, *Arabidopsis* contains five paralogous *GA20ox* genes (*AtGA20ox1*–*AtGA20ox5*), while rice contains four paralogous *GA20ox* genes (*OsGA20ox1*–*OsGA20ox4*). In the study, similar numbers of *CsGA20oxes* were isolated and identified, including *CsGA20ox1*–*CsGA20ox4*. In the next reaction via the *GA3ox* enzymes (EC: 1.14.11.15), GA_9_ and GA_20_ are hydroxylated to form the biologically active hormones GA_4_ and GA_1_ (Figure 1B) [1,65]. GA3ox enzymes are also encoded by multiple genes in all plant species. There are four GA3ox enzymes in *Arabidopsis* and two GA3ox enzymes in rice [6,60,66,67,68]. Three *CsGA3ox* isoforms have been confirmed and sequenced by RT-PCR amplification with genome-wide analysis in *C. sinensis*. The production of bioactive GAs has been shown to be limited by the *GA20ox* step [61,64,69,70]. There is controversy as to whether *GA3ox* may be a rate-limiting step in GA biosynthesis.

For group A, the most studied deactivation pathway is catalyzed by a class of *GA2ox* enzymes. [1,2,3,6,7,15,46,47,48,49]. In this study, seven *CsGA2ox*, isolated and identified in tea plants, were classified into three groups for the analysis of *Arabidopsis* genes (Figure 2). The present findings were consistent with findings by Lee and Zeevaart [51], who found that the *GA2ox* family can be divided into three classes on the basis of phylogenetic relationships. Members of class I and II catabolize C_19_-GAs, while class III members can only hydroxylate C_20_-GAs [6,11,15,38,71]. *Arabidopsis* has five C_19_*GA2ox* genes, including the group A1 (*AtGA2ox1*, *AtGA2ox2* and *AtGA2ox3*) and group A2 (class II) (*AtGA2ox4* and *AtGA2ox6*) [3,15,49]. The group A3 (class III) (*AtGA2ox7* and *AtGA2ox8*) belong to C_20_*GA2ox* genes [11]. The C_20_*GA2ox* subgroup only acts on C_20_-GA precursors, such as GA_12_ and GA_53_, to form GA_110_ and GA_97_, but not on C_19_-GAs. However, *SoGA2ox1* (*Spinacia oleracea*) can act on both C_19_-GA and C_20_-GA [72]. The C_20_*GA2ox* enzymes contain three unique conserved motifs that are absent in the class of C_19_*GA2ox* [51]. Motifs 5, 7 and 15 may be unique conserved motifs, which were found to be important for the activity of this class of *GA2ox* [37]. Deactivation mechanisms and pathways for effectively regulating bioactive hormone levels are critical for proper plant growth and development. Several different GA inactivation pathways have been revealed, which are critical for controlling endogenous GA levels. 

In the present study, the expression levels of 14 *CsGAox* genes were observed in tea plant tissues, although these levels varied greatly depending on the tissue type. In root tissues, *CsGAox* genes, such as *CsGA2ox1*, *CsGA2ox3*, *CsGA2ox4*, *CsGA2ox6*, *CsGA2ox7*, *CsGA3ox3*, *CsGA20ox3*, and *CsGA20ox4* showed maximum expression levels, while some other *CsGAox* genes, such as *CsGA2ox8*, *CsGA3ox1* and *CsGA20ox2*, showed several-fold lower expression compared to the other three tissues. The similar expression levels of all *CsGAox* genes have been showed in leaf and stem tissues with a few exceptions. From these results, the different expression levels in all the tea plant tissues is indicated that some *CsGAox* genes might play important roles in plant development and have unique functions in specific developmental stages.

Some candidate genes, which may play important response to low- and high-temperature, have been identified with regards to expression patterns. Heat maps show that *CsGAox* genes were downregulated in leaves after cold and heat treatments. Notably, *CsGA2ox3*, *CsGA2ox8*, *CsGA3ox2*, *CsGA3ox3*, and *CsGA20ox2* showed several-fold lower expression after temperature stress. In contrast, another *CsGAox* gene (*CsGA2ox1*) was induced after 12 h of cold stress, while *CsGA2ox2* and *CsGA2ox4* were induced after 12 h of heat stress. Similar to *Arabidopsis*, cold treatment also induced a transient increase in *AtGA2ox1* transcription levels [73]. However, *AtGA3ox1* gene is induced by cold temperatures in seeds soaked in the dark [74]. At high temperatures, *GA20ox* (*AtGA20ox1*, *AtGA20ox2*, and *AtGA20ox3*) and *GA3ox* (*AtGA3ox1* and *AtGA3ox2*) are suppressed, which leads to low levels of bioactive GAs [75]. High temperatures repress GA synthesis in *Arabidopsis* [1]. Meanwhile, cold treatments modulate GA metabolism via upregulation of *AtGA2ox3* and *AtGA2ox6* gene transcription levels, which decreases bioactive GAs [73].

Endogenous GA and ABA strongly influence tea plant growth [17,18,19]. The antagonistic roles played by GA and ABA regulate numerous developmental processes [54,55]. In *Arabidopsis* seeds, higher amounts of ABA were found to accumulate in GA-deficient *GA1-3* mutant seeds [76], whereas activation of GA biosynthesis genes, such as *AtGA3ox1* and *AtGA3ox2*, was observed in the *ABA2-2* mutant during seed development [77]. *AtGA3ox1* and *AtGA3ox2* were severely repressed at high temperatures, but their expression was completely de-repressed in ABA-deficient mutants [75]. In this study, seven genes containing *CsGA2ox3*, *CsGA2ox8*, *CsGA3ox2* and *CsGA20ox1-4* were upregulated or downregulated by at least four-fold after GA or ABA stress (Figure 6, Supplementary Figures S9 and S10). Most GA2oxes were upregulated by GA treatment [15,16], while most *GA20ox* and *GA3ox* genes were downregulated by applied GA [6,49]. ABA reduces bioactive GA levels by decreasing *AtGA20ox1* and increasing *AtGA2ox6* transcription levels [78]. Therefore, ABA plays a key role in the suppression of GA biosynthesis. Meanwhile, endogenous and exogenous ABA could induce GA deactivation through regulating the expression of some critical genes [79]. This evidence suggests that GAs play an important role in the abiotic stress response, which probably occurs through modulating the ABA signaling pathway [1]. 

We also examined *CsGAox* gene expression patterns in leaves under drought and high salinity stress. GAs influence drought tolerance in plants [80]. Almost all *CsGAoxes* had diminished expression levels during the 24-h treatment period. Notably, *CsGA2ox3*, *CsGA2ox6*, *CsGA2ox7*, *CsGA2ox8*, *CsGA3ox2*, and *CsGA3ox3* showed several-fold lower expression after PEG stress. However, the expression of *CsGA2ox1*, *CsGA3ox1*, *CsGA20ox1*, and *CsGA20ox3* was significantly induced at different points in time. *Arabidopsis* mutants over-expressing *AtGAox1* and *AtGAox2* exhibited decreased drought tolerance, while *AtGA20ox1 AtGA20ox2* and *AtGA3ox1 AtGA3ox2* double mutants, or the *AtGA20ox1 AtGA20ox2 AtGA20ox3* triple mutant presented dramatically increased drought tolerance compared to the wild-type *Arabidopsis* [80]. In *Arabidopsis*, six *AtGA2ox* genes were upregulated under high-salinity stress, including *AtGA2ox1*, *AtGA2ox2*, *AtGA2ox4*, *AtGA2ox6*, *AtGA2ox7* and *AtGA2ox8* [81]. In comparison, the transcription level of four genes (*CsGA2ox2*, *CsGA2ox6*, *CsGA20ox1* and *CsGA20ox3*) was downregulated by high salinity stresses after 4 h, but increased significantly (by at least five-fold) after that time point and maintained relatively high levels until 120 h. However, four genes (*CsGA2ox1*, *CsGA2ox3*, *CsGA3ox2* and *CsGA20ox2*) were downregulated by at least four-fold after 8 h of high salinity treatment (Figure 8). Additionally, *CsGA3ox3* rapidly increased by at least four-fold of expression after 72 and 120 h.

## 5. Conclusions

For the first time in tea plants, 14 *CsGAox* genes, containing the two domains of DIOX_N (PF14226) and 2OG-FeII_Oxy, were identified (PF03171), all of which were 2-oxoglutarate dependent dioxygenases (2-ODD). These included four *CsGA20ox* (EC: 1.14.11.12), three *CsGA3ox* (EC: 1.14.11.15) and seven *CsGA2ox* (EC: 1.14.11.13). These 14 *CsGAox* genes were phylogenetically clustered into three subfamilies and five subgroups (Figure 2). The genes in these subfamilies displayed differential expression profiles in the studied tissues. Each *CsGAox* shows tissue-specific expression patterns, although the level of expression varied greatly. Moreover, the qRT-PCR experiment was used to analyze the expression patterns of *CsGAox* genes in tea plants subjected to various abiotic stresses. Some candidate genes, which may play important roles in responding to cold, heat, PEG, high salinity, or exogenous GA or ABA stresses, have been identified with regards to expression patterns, such as *CsGA20ox2*, *CsGA3ox2*, *CsGA3ox3*, *CsGA2ox1*, *CsGA2ox2*, and *CsGA2ox4*. However, *CsGA2ox3*, *CsGA2ox6*, and *CsGA2ox8* may have some different expression patterns in response to external abiotic stresses. The production of bioactive GAs may be limited by the *GA20ox* step. It is controversial whether *GA3ox* may be a rate-limiting step in GA biosynthesis. Different *GA2ox* enzymes convert bioactive GAs and their precursors to limit bioactive GA levels and regulate many stages of plant development. In addition, the candidate genes could be used as marker genes for abiotic stress resistance breeding in tea plants.

## Figures and Tables

**Table 1:** The 14 *CsGAox* family genes in the tea plant.

Gene names	Accession Numbers	Gene ID ^a^	Number of Deduced Amino Acid	Molecular Weight (kDa)	Subcellular Location (WoLF PSORT/TargetP)	Groups	Theoretical pI
*CsGA20ox1*	KC193604	CSA007392	383	42.94	nucl	B	6.06
*CsGA20ox2*	KY296366	NF	376	43.02	nucl/cyto	B	6.98
*CsGA20ox3*	MF370231	CSA000105	378	43.03	nucl/cyto	B	6.94
*CsGA20ox4*	MF370232	CSA002490	356	40.31	cyto	B	5.30
*CsGA2ox1*	KY296367	CSA032124	333	37.18	nucl/cyto	A1	5.93
*CsGA2ox2*	KY296368	CSA026961	332	37.31	cyto/nucl	A1	7.61
*CsGA2ox3*	KY296369	CSA004444	334	37.56	nucl/cyto/chlo	A1	5.47
*CsGA2ox4*	MF370234	NF	344	38.67	nucl	A2	5.73
*CsGA2ox6*	MF370235	CSA014596	340	38.02	nucl/chol/mito	A2	7.94
*CsGA2ox7*	MF370236	CSA013052	346	39.24	cyto/nucl	A3	6.79
*CsGA2ox8*	MF370237	CSA034015	335	38.53	cyto/nucl	A3	5.33
*CsGA3ox1*	KF703743	CSA002905	361	40.29	chlo/mito/nucl	C	6.67
*CsGA3ox2*	KF703744	CSA020111	373	41.08	nucl/cyto	C	8.22
*CsGA3ox3*	MF370233	CSA034282	345	38.72	cyto/nucl	C	5.66

WoLF PSORT [38] and TargetP [39] were used to predict the subcellular localization of the 14 *CsGAox* family genes; the most likely locations are listed. nucl: nucleus; chlo: chloroplast; cyto: cytoplasmic; mito: mitochondrion. Groups were classified based on phylogenetic trees with their corresponding numbers in *Arabidopsis*. ^a^ Gene identifier of tea tree genome database [20] is available or not. NF: not found.

## Supplementary Materials

The following are available online at www.mdpi.com/2073-4425/8/9/235/s1. Table S1: Primers of *CsGAox* genes in RT-PCR. Table S2: Primers of *CsGAox* genes in qRT-PCR. Table S3: Comparison of the 14 *CsGAox* ORFs and putative amino acid sequences. Figure S1: Predicted amino acid sequence alignment and phylogenetic tree of the *CsGA20oxes* with the GA20oxes from other plant species. Figure S2: Predicted amino acid sequence alignment and phylogenetic tree of the *CsGA3oxes* with the GA3oxes from other plant species. Figure S3: Predicted amino acid sequence alignment and phylogenetic tree of the *CsGA2ox1*, *2*, *3* with the GA2oxes from other plant species. Figure S4: Predicted amino acid sequence alignment and phylogenetic tree of the *CsGA2ox4*, *6* with the GA2oxes from other plant species. Figure S5: Predicted amino acid sequence alignment and phylogenetic tree of the *CsGA2ox7*, *8* with the GA2oxes from other plant species. Figure S6: Tissue-specific expression profiles of *CsGAoxes*. Figure S7: Expression profiles of *CsGAox* genes in tea plants under cold stresses in leaves. Figure S8: Expression profiles of *CsGAox* genes in tea plants under heat stresses in leaves. Figure S9: Expression profiles of *CsGAox* genes in tea plants under exogenous GA stresses in leaves. Figure S10: Expression profiles of *CsGAox* genes in tea plants under exogenous ABA stresses in leaves. List of protein sequences used in this study: List of putative *GAox* genes in *Sorghum bicolor*, *Physcomitrella patens*, *Populus trichocarpa*, *Cucumis sativus*, *Medicago truncatula*, *G.max*, *Citrus sinensis*, *Vitis vinifera*, *Zea mays*, *Selaginella moellendorffii*, and Arabidopsis.
