# Supplementary material for: Genome-Wide Analysis of the Biosynthesis and Deactivation of Gibberellin-Dioxygenases Gene Family in Camellia sinensis (L.) O. Kuntze
Source: Genes (Basel). 2017 Sep 19;8(9):235. doi: 10.3390/genes8090235 (PMC5615368; doi:10.3390/genes8090235)
Supplement: Supplementary file 1 [file genes-08-00235-s001.doc]

**Supplemental material**

**Table S1. Primers of *CsGAox* genes in RT-PCR.**

| **Genes** | **Forward primer sequence (5′— 3′)** | **Reverse primer sequence (5′—3′)** |
| --- | --- | --- |
| *CsGA20ox1* | — | — |
| *CsGA20ox2* | ATGGACTTGAGTGTCTCAATG | TCAAGTGGGTTTGGAAGAT |
| *CsGA20ox3* | ATGGACTCAAGTTCCCCAACTC | AGGAAAGAGTTAGTTGGGGGTG |
| *CsGA20ox4* | ATGGCACCGGCAATTGA | TCAAGGAGAAATGAGATATCTGTTAAG |
| *CsGA3ox1* | — | — |
| *CsGA3ox2* | — | — |
| *CsGA3ox3* | ATTATCAAAGCACCCACC | GTTCCAACTCATAACCTTAC |
| *CsGA2ox1* | ATGGTGGTTCTCGCTAA | AATAAGGTGGGGACAA |
| *CsGA2ox2* | CATCATCCTGCACTTC | TTATGAGGCTGCTATTT |
| *CsGA2ox3* | TCTCCATAACAACCACAAC | CCATCTATGCCAAGTTTAGTCAA |
| *CsGA2ox4* | CTCTTACTCCAAGCCCATAG | CGTTCTTAACTGCAAAATCG |
| *CsGA2ox6* | TCTTCCATTTCATTCCTCCTA | CCTGCTGACATCAGAGCATG |
| *CsGA2ox7* | CTTCATCACCCATAGGACAA | CTAACGCAGAAATCTGGGA |
| *CsGA2ox8* | TCTATAATGCAGGACCAATG | ATCACCTTCCCAAACTAAAC |

Note: ‘－’ means no primers because of the genes been submitted to NCBI by Yue C (Direct Submission).

**Table S2. Primers of *CsGAox* genes in qRT-PCR.**

| **Genes** | **Forward primer sequence (5′—3′)** | **Reverse primer sequence (5′—3′)** |
| --- | --- | --- |
| *CsGA20ox1* | CGGCTTCTTTCTCGTAGTCAA | GAACAGTTTTCCTCAGCAGAGTATC |
| *CsGA20ox2* | AAAGCTGTGTGCATCGGG | GAGTGAAATGGAGCAAATCGG |
| *CsGA20ox3* | GCAGTGGTGAACAAGGAGA | AGCTTTGGAGGGTAGTGAGA |
| *CsGA20ox4* | TTAGTGAGGCAATGGTGGAGTA | GTAAGGGGCAAGGAGGGTA |
| *CsGA3ox1* | TGCTCGGATTTCTCCCTTCT | GCCCATTTGACATCTTCTTTG |
| *CsGA3ox2* | TACTCTCGTGGTCAACATAGGG | AGGAGGGTGGCTTGGGTC |
| *CsGA3ox3* | CTCCACCATTCCAACACC | GCAGACCATAAAAATACCCCATAG |
| *CsGA2ox1* | GATGGCAACTGGATTTCTGTC | GAGTGAAGGCAATGGGGC |
| *CsGA2ox2* | TGGATTTGGAGAGCACACTG | CAACAACCCTATGCCTCACA |
| *CsGA2ox3* | GAATACCTCCTCTTCAGCAGC | CTCAATCTTCAACCCATCAGC |
| *CsGA2ox4* | CCAGACGCATCAGTGTTTAGT | CAGAAGGCAGTAGTGGGGTA |
| *CsGA2ox6* | GGCTCAATTACTACCCTCCTCTC | GGAACCCACAACCCATCATCT |
| *CsGA2ox7* | GGGTCACCAAAAGGAGGGA | GCGATTGGGTTTGACAGCA |
| *CsGA2ox8* | GAGCCACCATTTCAAGACACT | TCACCACTTGGAAGAACCC |
| *CsActin* | GCCATCTTTGATTGGAATGG | GGTGCCACAACCTTGATCTT |

**Table S3.** Comparison of the 14 *CsGAox* ORFs and putative amino acid sequences.

|  | Putative protein sequences identity (％) | | | | | | | | | | | | | |
| --- | --- | --- | --- | --- | --- | --- | --- | --- | --- | --- | --- | --- | --- | --- |
|  | *CsGA20ox1* | *CsGA20ox2* | *CsGA20ox3* | *CsGA20ox4* | *CsGA2ox1* | *CsGA2ox2* | *CsGA2ox3* | *CsGA2ox4* | *CsGA2ox6* | *CsGA2ox7* | *CsGA2ox8* | *CsGA3ox1* | *CsGA3ox2* | *CsGA3ox3* |
| ***CsGA20ox1*** | 100.0 | 52.0 | 53.2 | 33.2 | 27.7 | 29.1 | 26.7 | 29.4 | 30.6 | 29.7 | 28.5 | 28.9 | 29.7 | 25.8 |
| ***CsGA20ox2*** | 58.7 | 100.0 | 65.9 | 30.9 | 26.4 | 27.4 | 25.4 | 27.7 | 26.2 | 27.3 | 26.9 | 29.7 | 28.4 | 24.7 |
| ***CsGA20ox3*** | 59.6 | 71.7 | 100.0 | 32.6 | 27.3 | 27.4 | 25.9 | 28.6 | 28.8 | 26.1 | 28.3 | 31.6 | 31.7 | 25.7 |
| ***CsGA20ox4*** | 42.8 | 45.8 | 44.8 | 100.0 | 29.6 | 30.6 | 28.3 | 29.7 | 30.3 | 27.9 | 30.7 | 25.1 | 27.7 | 22.6 |
| ***CsGA2ox1*** | 42.0 | 43.6 | 42.9 | 44.9 | 100.0 | 86.1 | 70.0 | 52.6 | 52.6 | 25.5 | 25.2 | 28.2 | 27.4 | 28.4 |
| ***CsGA2ox2*** | 42.3 | 44.0 | 42.9 | 45.6 | 87.0 | 100.0 | 68.7 | 51.1 | 50.9 | 25.9 | 25.7 | 27.6 | 26.8 | 28.9 |
| ***CsGA2ox3*** | 40.8 | 41.0 | 41.1 | 44.1 | 74.4 | 73.8 | 100.0 | 48.8 | 51.1 | 22.3 | 23.3 | 27.4 | 27.9 | 28.1 |
| ***CsGA2ox4*** | 41.8 | 41.4 | 40.4 | 45.8 | 58.8 | 58.2 | 56.7 | 100 | 62.7 | 28.1 | 26.7 | 27.5 | 27.4 | 27.3 |
| ***CsGA2ox6*** | 42.0 | 40.7 | 41.3 | 45.0 | 59.3 | 59.8 | 58.0 | 67.9 | 100.0 | 26.4 | 25.4 | 26.2 | 26.5 | 27.3 |
| ***CsGA2ox7*** | 42.1 | 43.8 | 41.4 | 40.7 | 41.9 | 41.3 | 39.5 | 40.3 | 39.3 | 100.0 | 54.2 | 28.3 | 29.1 | 27.5 |
| ***CsGA2ox8*** | 42.1 | 43.3 | 41.9 | 44.6 | 41.7 | 41.7 | 40.6 | 42.8 | 42.5 | 63.1 | 100.0 | 26.4 | 25.6 | 27.7 |
| ***CsGA3ox1*** | 39.2 | 41.5 | 39.0 | 40.5 | 41.4 | 41.3 | 39.0 | 41.7 | 40.2 | 39.9 | 39.3 | 100.0 | 81.5 | 48.8 |
| ***CsGA3ox2*** | 40.4 | 40.7 | 40.0 | 40.5 | 42.3 | 42.1 | 39.8 | 41.0 | 40.5 | 39.1 | 38.9 | 82.6 | 100.0 | 50.4 |
| ***CsGA3ox3*** | 39.0 | 42.8 | 41.5 | 39.1 | 41.5 | 59.5 | 58.5 | 42.8 | 41.5 | 39.1 | 41.5 | 59.5 | 58.5 | 100.0 |
|  | ORF sequences identity (％) | | | | | | | | | | | | | |

Multiple sequence alignments were analyzed using ClustalW. The sequence similarities in the ORFs and amino acid sequences are listed.


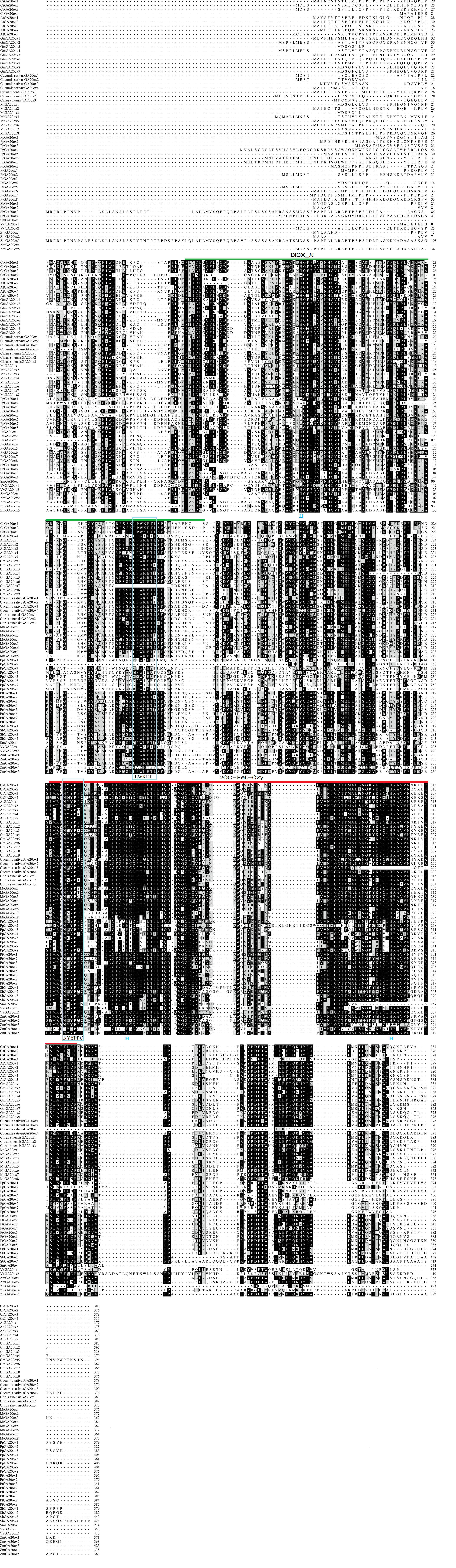

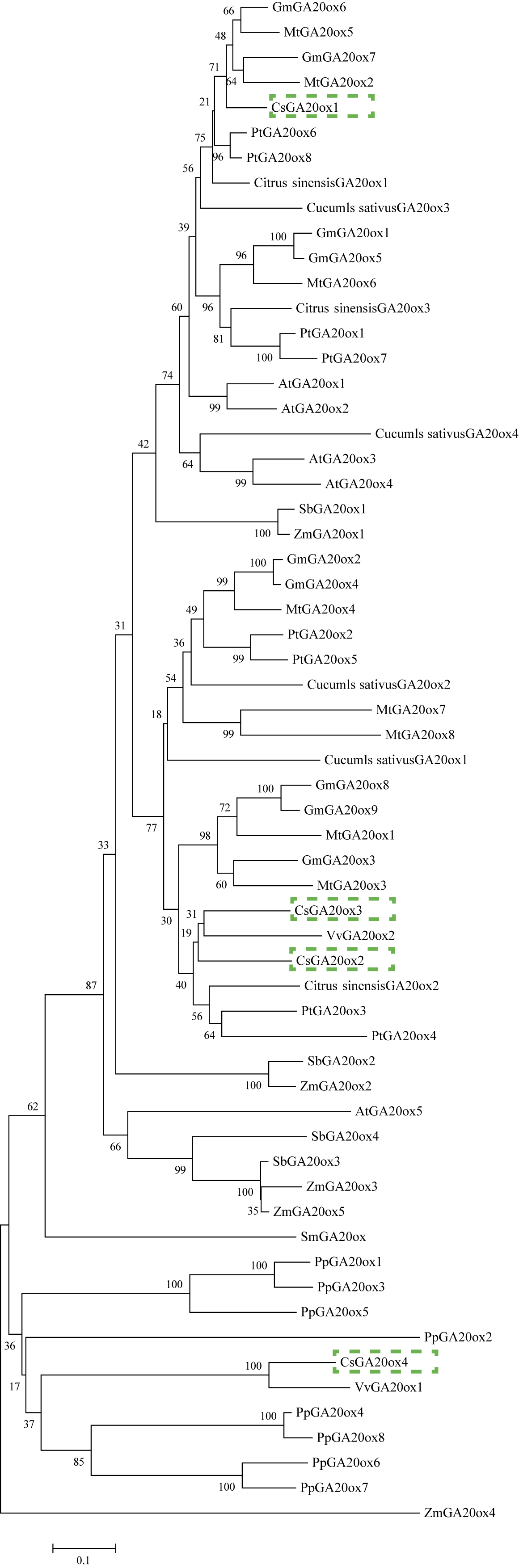


**Figure S1.** Predicted amino acid sequence alignment and phylogenetic tree of the *CsGA20oxes* with the GA20oxes from other plant species.


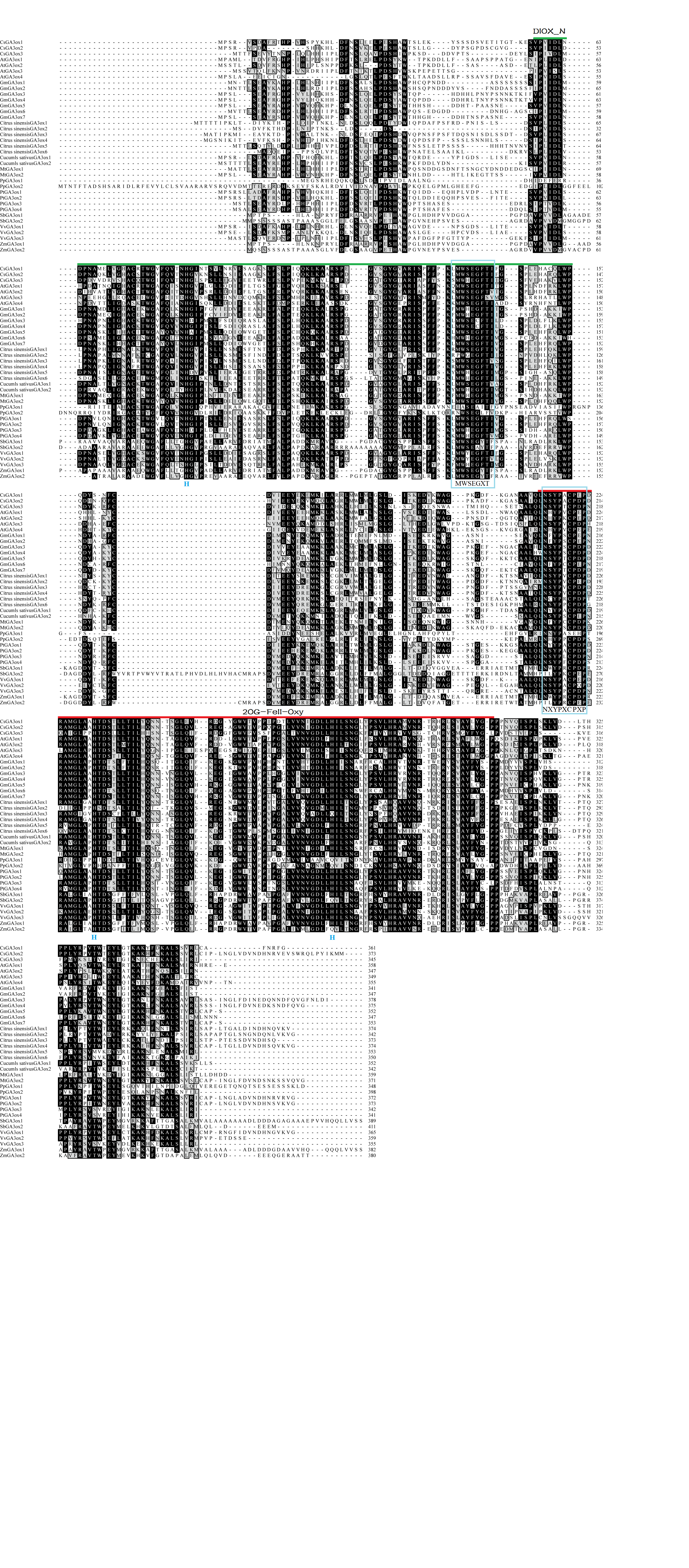

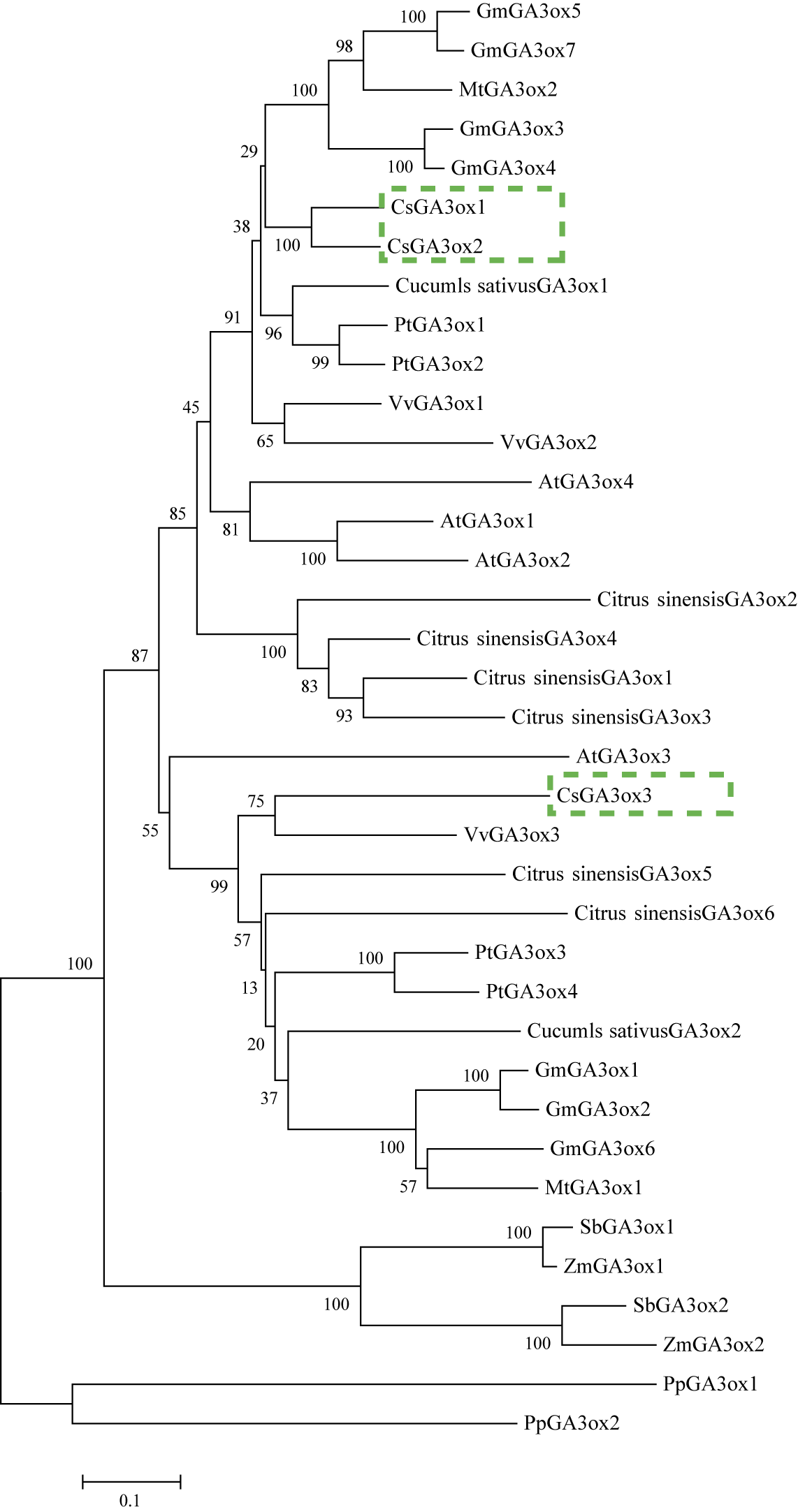


**Figure S2.** Predicted amino acid sequence alignment and phylogenetic tree of the *CsGA3oxes* with the GA3oxes from other plant species.


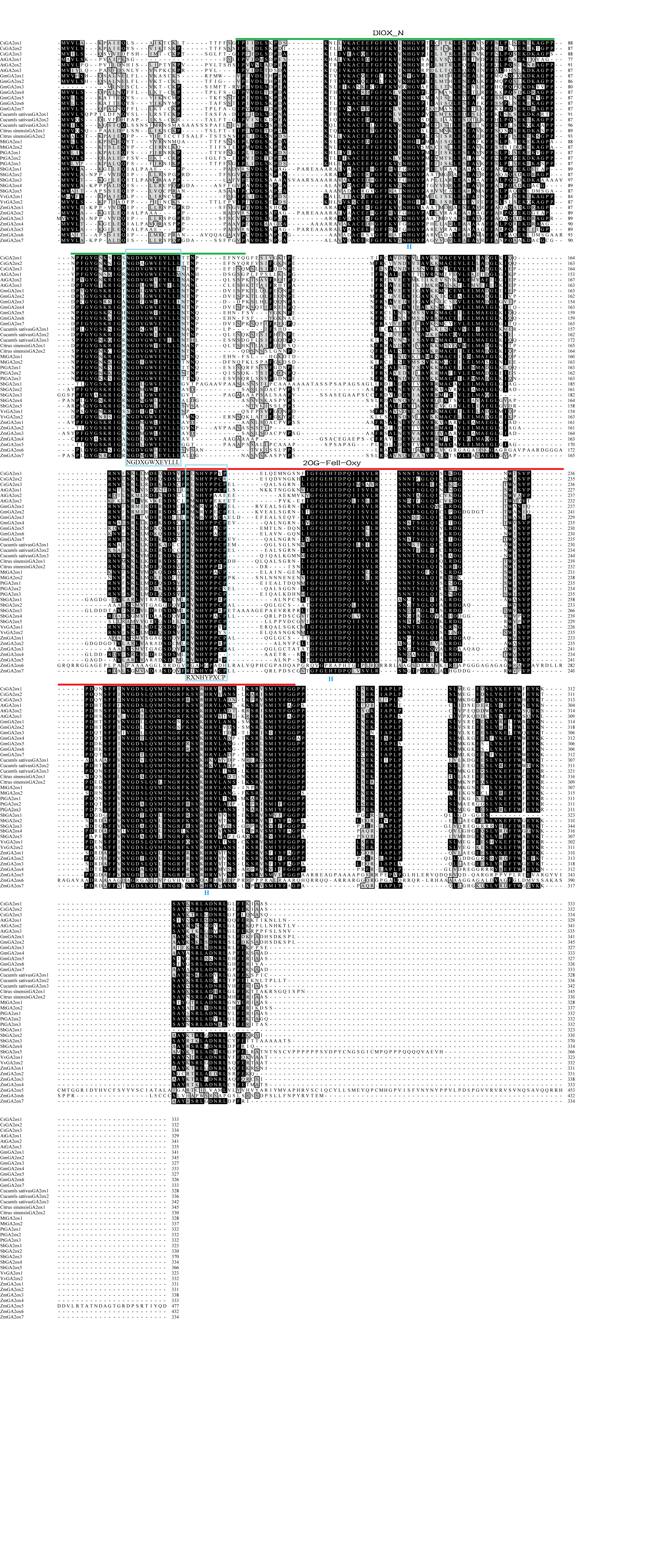

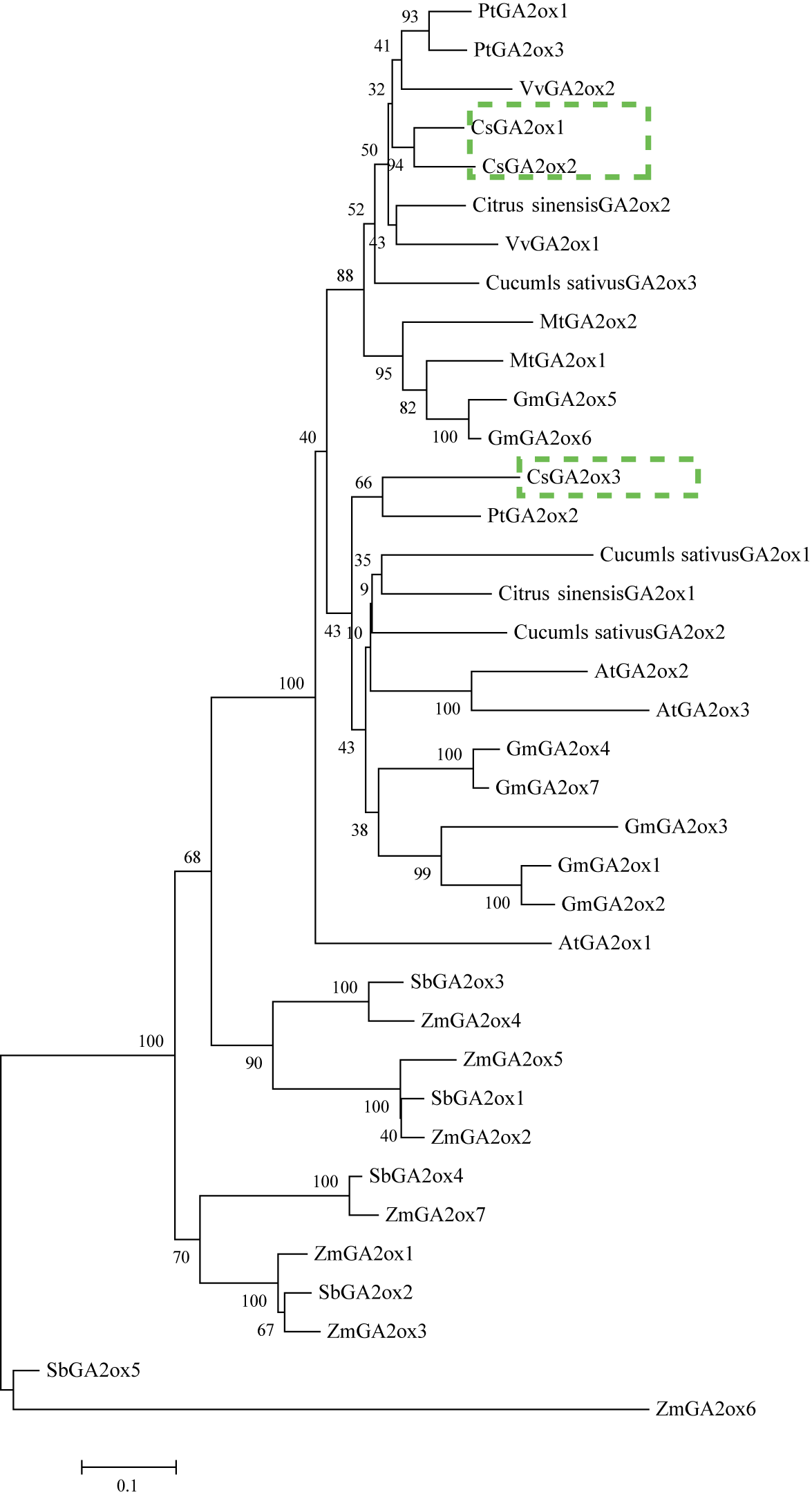


**Figure S3.** Predicted amino acid sequence alignment and phylogenetic tree of the *CsGA2ox1*, *2*, *3* with the GA2oxes from other plant species.


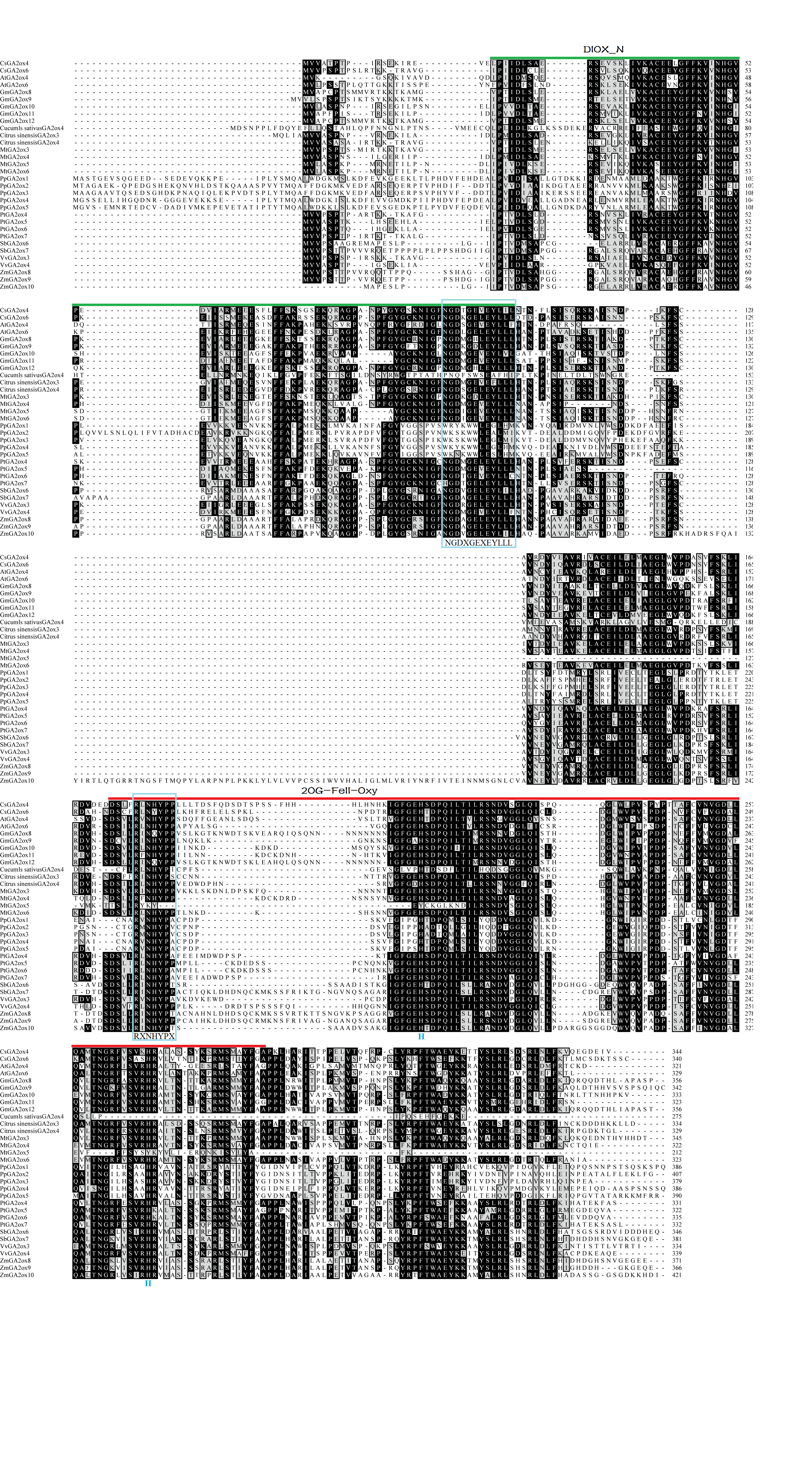

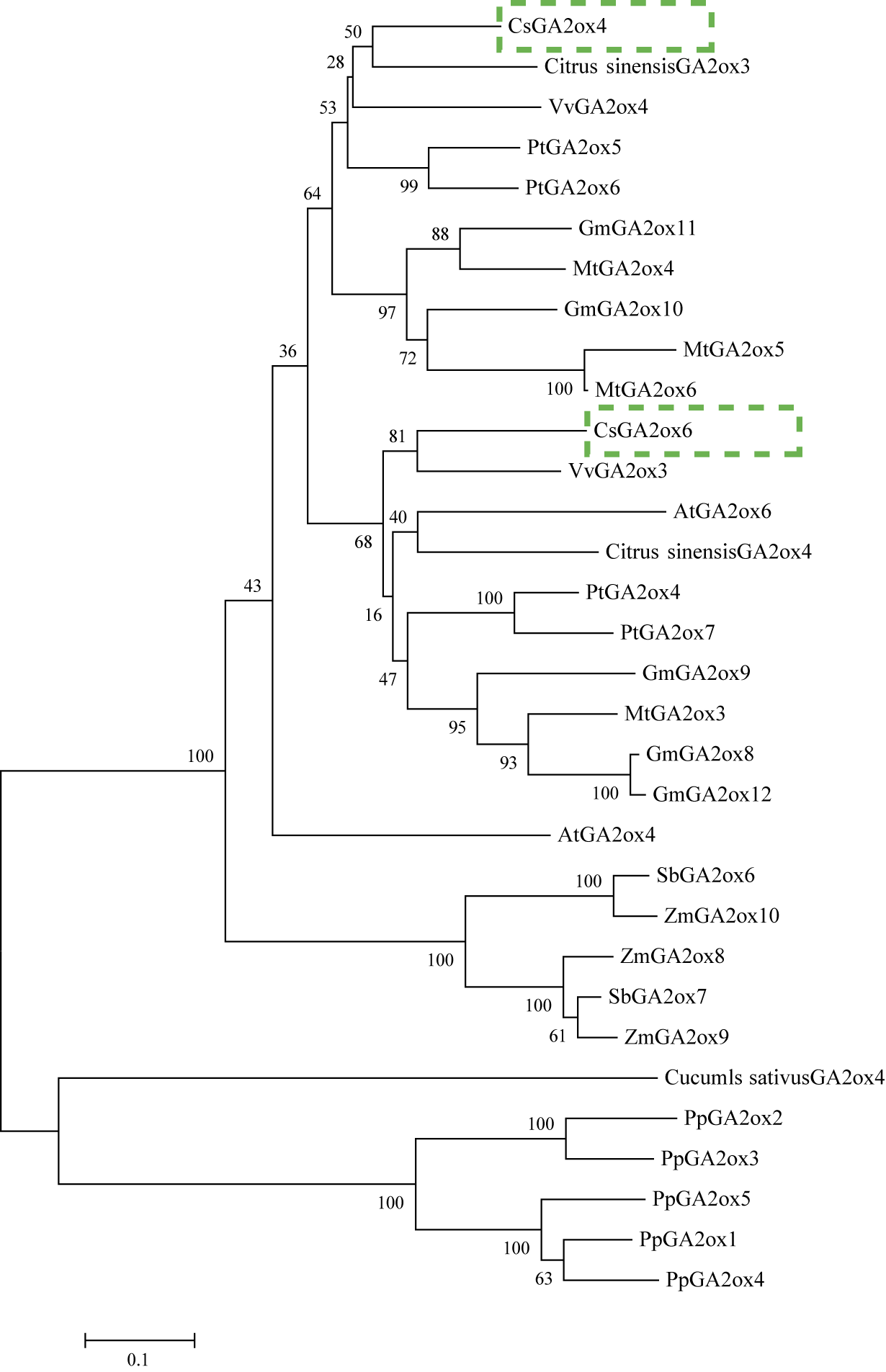


**Figure S4.** Predicted amino acid sequence alignment and phylogenetic tree of the *CsGA2ox4*, *6* with the GA2oxes from other plant species.


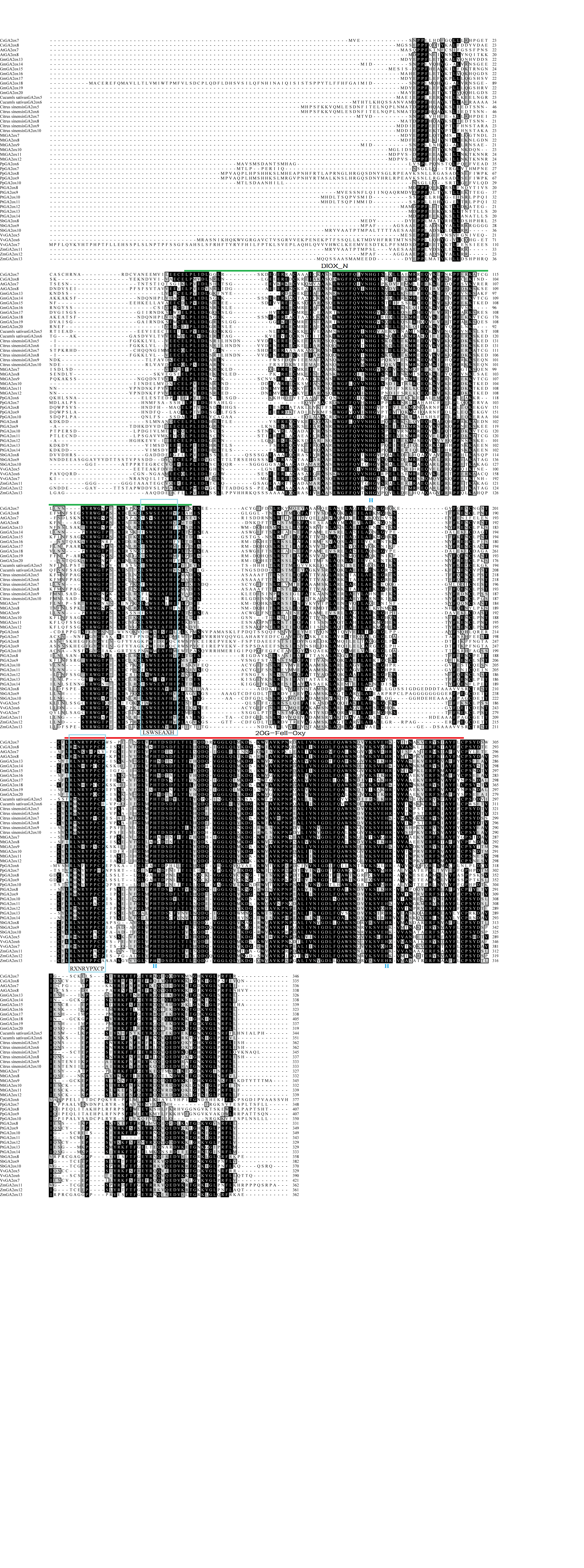

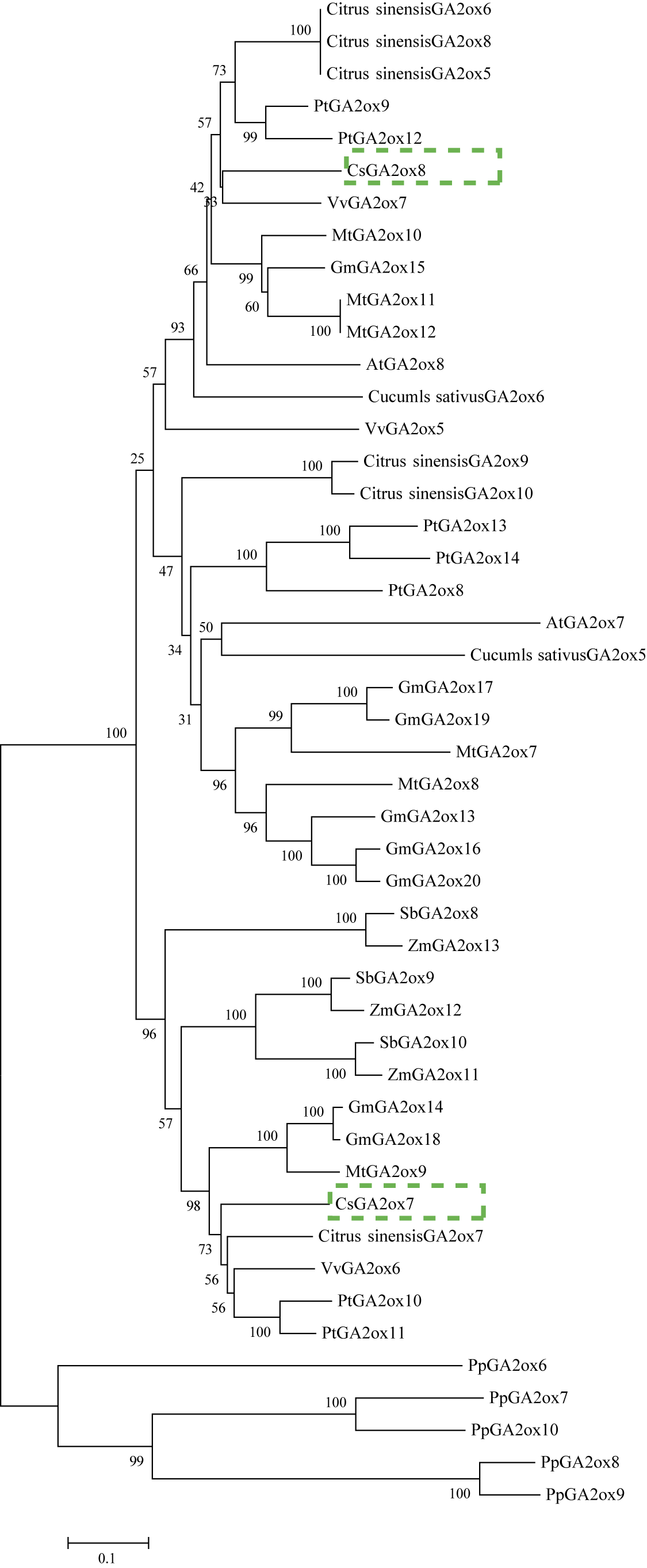


**Figure S5.** Predicted amino acid sequence alignment and phylogenetic tree of the *CsGA2ox7*, *8* with the GA2oxes from other plant species.

**
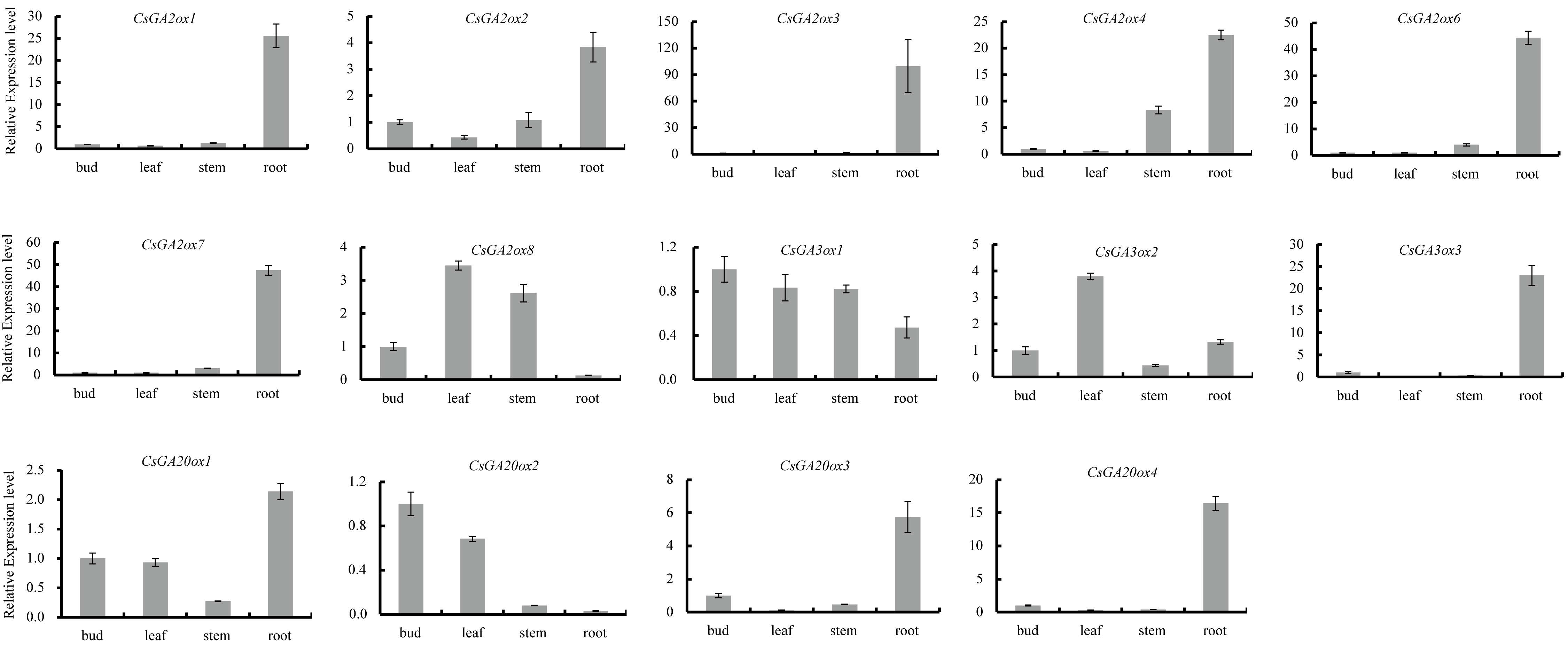
**

**Figure S6.** Tissue-specific expression profiles of *CsGAoxes.*


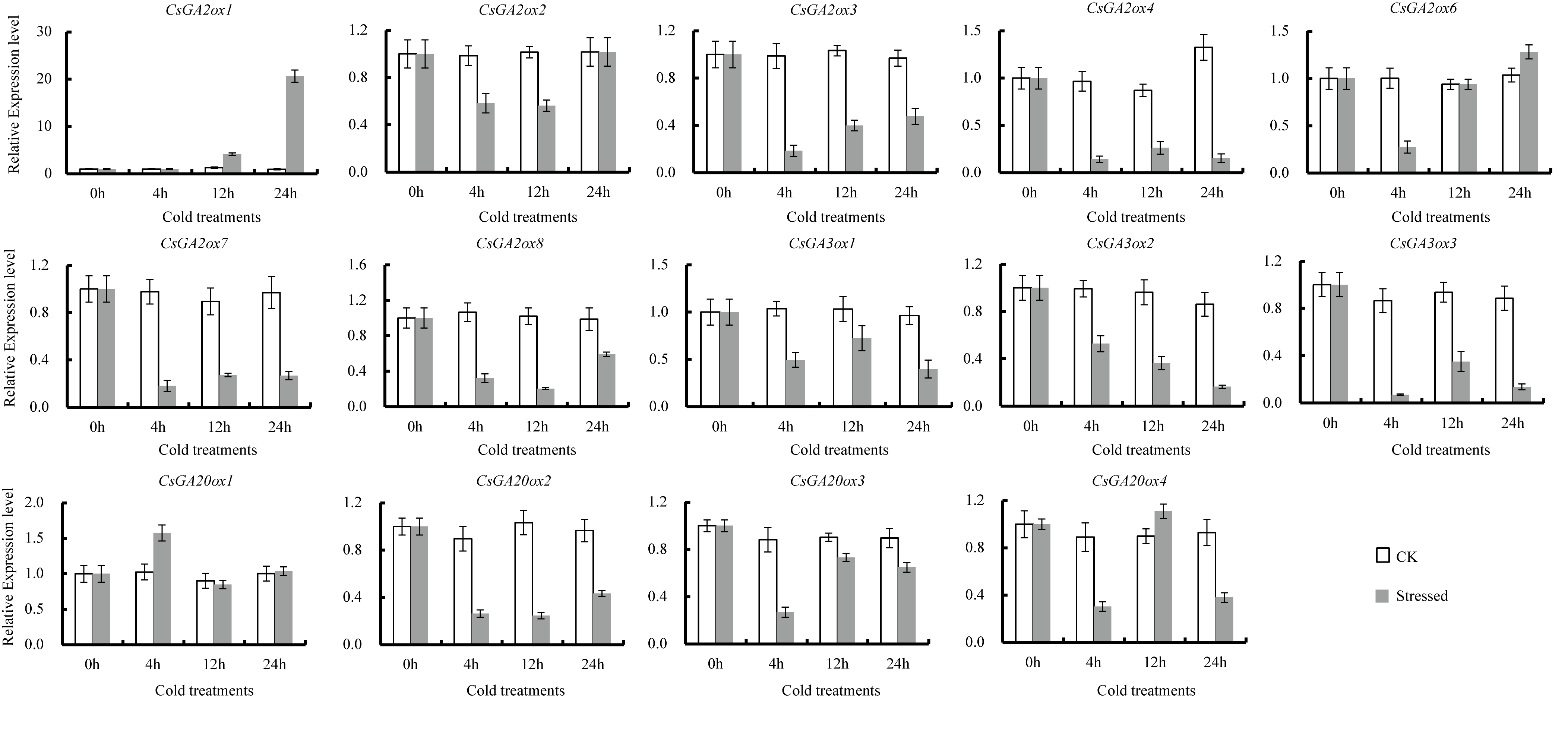


**Figure S7.** Expression profiles of *CsGAox* genes in tea plants under cold stresses in leaves.


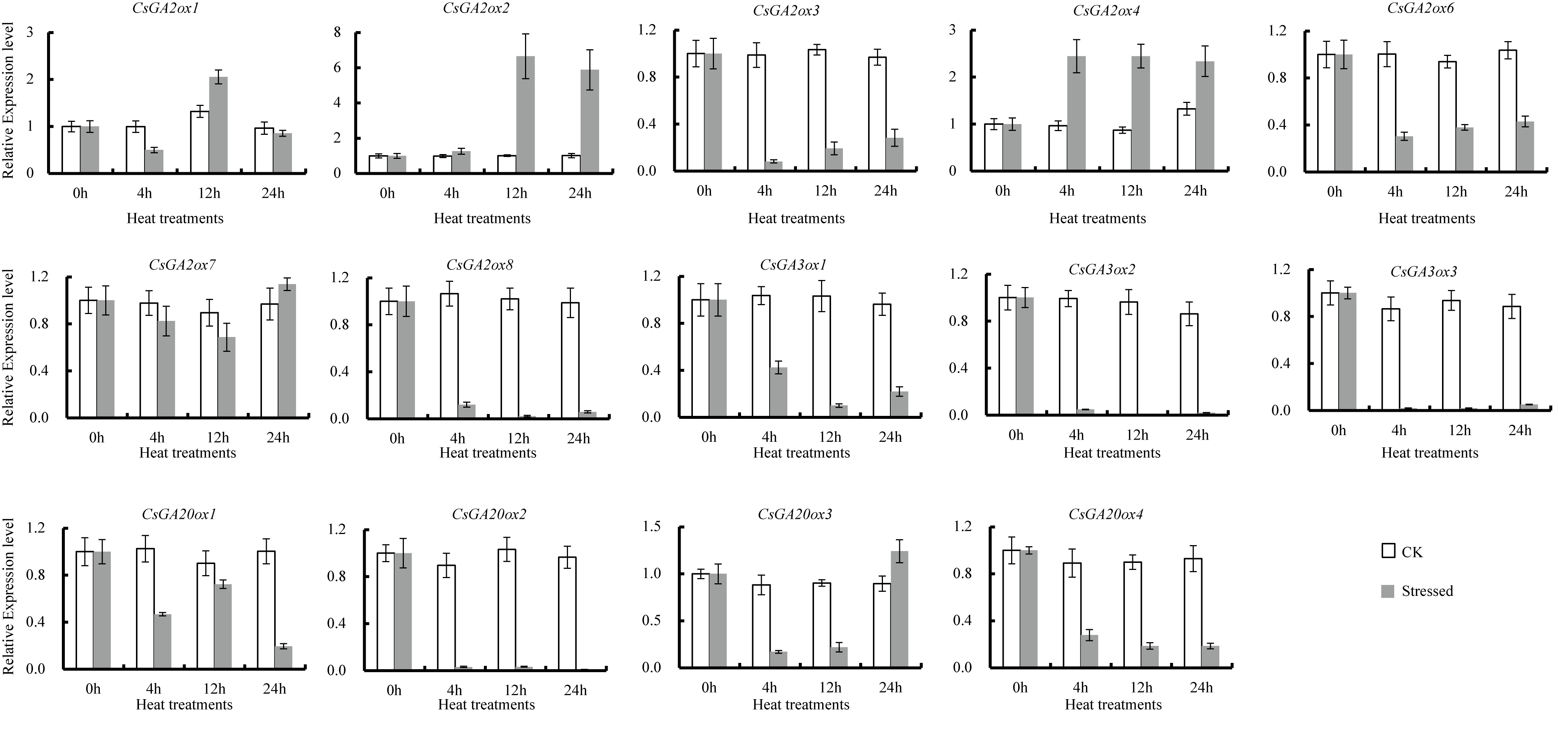


**Figure S8.** Expression profiles of *CsGAox* genes in tea plants under heat stresses in leaves.


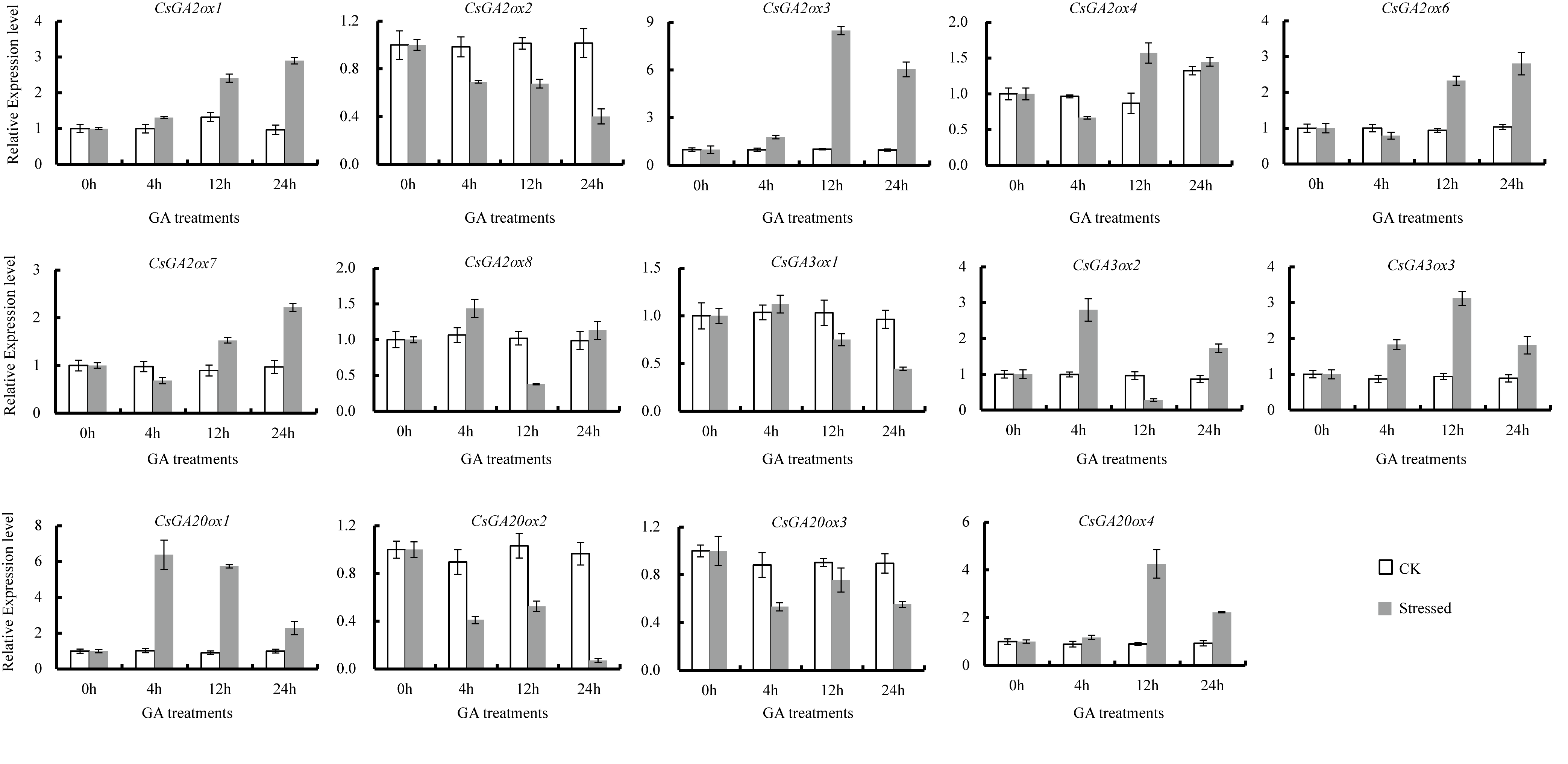


**Figure S9.** Expression profiles of *CsGAox* genes in tea plants under exogenous GA stresses in leaves.


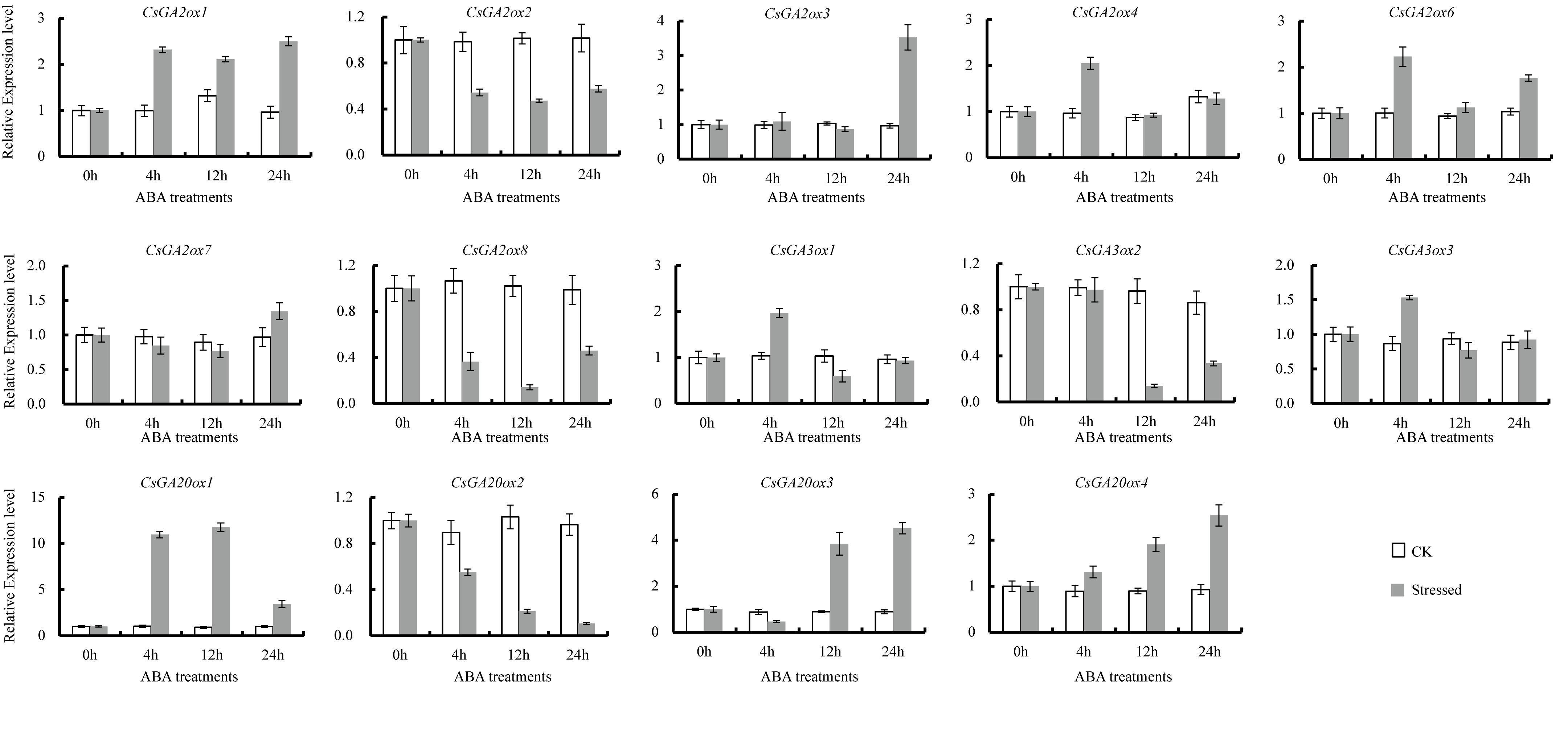


**Figure S10.** Expression profiles of *CsGAox* genes in tea plants under exogenous ABA stresses in leaves.

**List of protein sequences used in this study**: List of putative *GAox* genes in *Sorghum bicolor*, *Physcomitrella patens*, *Populus trichocarpa*, *Cucumls sativus*, *Medicago truncatula*, *G.max*, *Citrus sinensis*, *Vitis vinifera*, *Zea mays*, *Selaginella moellendorffii* and *Arabidopsis* database

**GA20ox protein (EC: 1.14.11.12)**

>CSA007392 CsGA20ox1

MAINCVTNTLSMSPPPPPPPLPKDDQPLVFDASVLRHEQNIPAQFIWPDHEKPCSTAPELSVPLVDLGGFLSGDPKAAAEASRVVGEACRKHGFFLVVNHGVDATLISDAHSNMDSFFELPLCEKQRVQRKVGEHCGYASSFTGRFSSKLPWKETLSFRYSAEENCSSIVNDYFMNRMGENFVQFGKVYQDYCNAMSTLSLGIMELLGMSLGVSRAHFKEFFEENDSIMRLNYYPPCQKPDQTLGTGPHCDPTSLTILHQDTVGGLQVFVDDQWHSISPNFNAFVVNIGDTFMALSNGRYKSCLHRAVVNNKTPRKSLAFFLCPKKDKVVSPPTELVDGKNPRIYPDFTWPTLLEFTQKHYRADMKTLEAFSNWVQQKTAEVA

>CsGA20ox2

MDLSVSMLQCSPLEHSDHINYESSFSSSSIFLKHTNIPKKFIWPIEDTIQAHEELKEPVVDLEGFLKGDELATQHAAKLIRASCLSHGFFQVINHGIDSSLIRQAEDHMNAFFKLPIDKKMRAQKKPGCMWGYSGAHGDRFSSKLPWKETLSFGFHENGSDPVVVDFFRSALGKEFEHTGLVYQKYCESMKRLSLAIMEILGISLGLVDQVCYKEFFKDSKSIMRCNYYPPCQEPELALGTGPHCDPTSLTILHQDQVGGLEVFVNNKWQSVQPIHGALVINIGDTFMALTNGRYKSCVHRAVVNRYKERTSLAFFVCPKEDKVVRPPLELVRRERPRLYPDFTWSDLLHFTQNHYRADDTTLQKFSTWFLSSKPT

>CSA000105 CsGA20ox3

MDSSSPTLLLCPPPIEIKDEREKKVLYFDTSLLQKQPTLPTEFIWPHKDLLHTQEELSEPLVDLEGFFKGDEAATSCAAKLVKTACLNHGFFQVTNHGVDKGLIRAAHDHMDAFFKLPINKKLRARRKPGSVCGYSGAHADRFKSKLPWKETLSFGYHGNGPVVVDYFKSILGDDFKETGLIFERYCEEMRRLSLALMELLAISLGIERLHYRKFFADGSSIMRCNYYPPCKEPGLTLGTGPHCDPTSLTILHQDEVGGLEVFANNKWQGVRPRPDALVINIGDTFMALCNGRYKSCMHRAVVNKERERRSLAYFVCPREDKVVSPPQDLIDREGGDEGPRKYPDFTWSDLLHFTQTHYRADLTTLQSFFLFHLNTPN

>CSA002490 CsGA20ox4

MAPAIEEEHVDSLSSNGQKLCIKNFIWSDSEWPQINYDHFDDRDDIPLISLQKDQNCEKVCRDMVAASNKWGFFKLVDHGVPSEIVENMKARLNEMFDLPMEEKLKGARSATMPLGYCATNPDYGKNLPWAEILQLLQSPQQVLGFAKKVFGDQHQPFSEAMVEYMEALDKLGMTIMEMLANGLGLQSDFFTKNFEEKDSTMIRINKYPPCPLPEKCLGVGSHTDPHTLTILLADNVGGLQVLKDDNQWVGIRPIPNSFVINIGDTLEAWTNGRLKSVIHRAVVNKEKSRLSIAYFLSPALSTIIDSPPELLEDPNTDPPINRKYVPFTWADFRKELLIQKRVIGKTALNRYLISP

>AT4G25420 AtGA20ox1

MAVSFVTTSPEEEDKPKLGLGNIQTPLIFNPSMLNLQANIPNQFIWPDDEKPSINVLELDVPLIDLQNLLSDPSSTLDASRLISEACKKHGFFLVVNHGISEELISDAHEYTSRFFDMPLSEKQRVLRKSGESVGYASSFTGRFSTKLPWKETLSFRFCDDMSRSKSVQDYFCDALGHGFQPFGKVYQEYCEAMSSLSLKIMELLGLSLGVKRDYFREFFEENDSIMRLNYYPPCIKPDLTLGTGPHCDPTSLTILHQDHVNGLQVFVENQWRSIRPNPKAFVVNIGDTFMALSNDRYKSCLHRAVVNSESERKSLAFFLCPKKDRVVTPPRELLDSITSRRYPDFTWSMFLEFTQKHYRADMNTLQAFSDWLTKPI

>AT5G51810 AtGA20ox2

MAILCTTTSPAEKEHEPKQDLEKDQTSPLIFNPSLLNLQSQIPNQFIWPDEEKPSIDIPELNVPFIDLSSQDSTLEAPRVIAEACTKHGFFLVVNHGVSESLIADAHRLMESFFDMPLAGKQKAQRKPGESCGYASSFTGRFSTKLPWKETLSFQFSNDNSGSRTVQDYFSDTLGQEFEQFGKVYQDYCEAMSSLSLKIMELLGLSLGVNRDYFRGFFEENDSIMRLNHYPPCQTPDLTLGTGPHCDPSSLTILHQDHVNGLQVFVDNQWQSIRPNPKAFVVNIGDTFMALSNGIFKSCLHRAVVNRESARKSMAFFLCPKKDKVVKPPSDILEKMKTRKYPDFTWSMFLEFTQKHYRADVNTLDSFSNWVITNNNPI

>AT5G07200 AtGA20ox3

MATECIATVPQIFSENKTKEDSSIFDAKLLNQHSHHIPQQFVWPDHEKPSTDVQPLQVPLIDLAGFLSGDSCLASEATRLVSKAATKHGFFLITNHGVDESLLSRAYLHMDSFFKAPACEKQKAQRKWGESSGYASSFVGRFSSKLPWKETLSFKFSPEEKIHSQTVKDFVSKKMGDGYEDFGKVYQEYAEAMNTLSLKIMELLGMSLGVERRYFKEFFEDSDSIFRLNYYPQCKQPELALGTGPHCDPTSLTILHQDQVGGLQVFVDNKWQSIPPNPHAFVVNIGDTFMALTNGRYKSCLHRAVVNSERERKTFAFFLCPKGEKVVKPPEELVNGVKSGERKYPDFTWSMFLEFTQKHYRADMNTLDEFSIWLKNRRSF

>AT1G60980 AtGA20ox4

MECIIKLPQRFNKNKSKKNPLRIFDSTVLNHQPDHIPQEFVWPDHEKPSKNVPILQVPVIDLAGFLSNDPLLVSEAERLVSEAAKKHGFFLVTNHGVDERLLSTAHKLMDTFFKSPNYEKLKAQRKVGETTGYASSFVGRFKENLPWKETLSFSFSPTEKSENYSQTVKNYISKTMGDGYKDFGSVYQEYAETMSNLSLKIMELLGMSLGIKREHFREFFEDNESIFRLNYYPKCKQPDLVLGTGPHCDPTSLTILQQDQVSGLQVFVDNQWQSIPPIPQALVVNIGDTLMALTNGIYKSCLHRAVVNGETTRKTLAFFLCPKVDKVVKPPSELEGERAYPDFTWSMFLEFTMKHYRADMNTLEEFTNWLKNKGSF

>AT1G44090 AtGA20ox5

MCIYASRQTVCPYLTPFKVKRPKSREMNSSDVNFSLLQSQPNVPAEFFWPEKDVAPSEGDLDLPIIDLSGFLNGNEAETQLAAKAVKKACMAHGTFLVVNHGFKSGLAEKALEISSLFFGLSKDEKLRAYRIPGNISGYTAGHSQRFSSNLPWNETLTLAFKKGPPHVVEDFLTSRLGNHRQEIGQVFQEFCDAMNGLVMDLMELLGISMGLKDRTYYRRFFEDGSGIFRCNYYPPCKQPEKALGVGPHNDPTAITVLLQDDVVGLEVFAAGSWQTVRPRPGALVVNVGDTFMALSNGNYRSCYHRAVVNKEKVRRSLVFFSCPREDKIIVPPPELVEGEEASRKYPDFTWAQLQKFTQSGYRVDNTTLHNFSSWLVSNSDKKST

>GM03G019800 GmGA20ox1

MLVPHHPSMLIAPQNTSAENHDNMEGQKQLHHFDESLMPNQSNIPSQFIWPDHEKPCLTPPELHIPPIDLKAFLSGDPQAVSAICAEANEACKKHGFFLVVNHGVDRKLIAQAHKLIDDFFCMQLSQKQKAQRKIGEHCGYANSFIGRFSSKLPWKETLSFHYSADKSSKSVEDYFLNVMGEDFRKFGSVFQEYCEAMSKLSLGIMELLGMTLGVGRECFRDFFEGNESVMRLNYYPPCQKPELALGTGPHCDPTSLTILHQDQVEGLQVFVDGRWYSVAPKEDAFVVNIGDTFMALSNGLFKSCMHRAVVNNKIVRKSLAFFLCPNRDKVVTPPKDLISNENPRTYPDFTWPSLLEFTQKHYRSDTETLDAFSRWLLEKNN

>GM04G211100 GmGA20ox2

MSPPLMESSASTLVIYPASQPQQEPKNENNGGIVFGSNLQQMQGEMPKEFLWPSRDLVDTTQEELKEPLVDLAIFKNGDEKAIANAAELVRTACLKHGFFQVINHGVDPDLIDAAYHEIDSIFKLPLSKKMGAKRKPGGVSGYSGAHADRYSSKLPWKETFSFLYDHQSFSNSQIVDNFKSVLGEDLQHTGRVYQKYCEAMKDLSLVIMELLAISLGVDRGHYRRFFEDGDSIMRCNYYPPCNSANLTLGTGPHTDPTSLTILHQDQVGGLEVFVDNKWFAVRPRSEALVINIGDTFMALSNGRYKSCLHRALVNTYRERRSLVYFVCPREDKIVRPPDNLLCRNEERKYPDFTWSNLFEFTQKHYRADVATLQSFIEWQQCSNSKSKPSNF

>GM04G244200 GmGA20ox3

MDSGGLLRCELEVLPHVPTNFIWPKEYLVDAQHELQAPVVDLYGFLRGENEATKHAAKLISEACLKHGFFQVINHGVDPHLIRQAHDQMDTFFKLPIHRKLSVHKTPGSMWGYSGAHAHRFSSQLPWKETLSFPYHDNTLEPVVTNYFKSTIGEDFEQTGETFQKYCGAMKQLGMKLIELLAMSLGVDRLHYRDLFEEGCSIMRCNNYPSCQQPSLTLGTGPHCDPTSLTILHQDHVGGLHVFADNKWQTVPPRLDAFVVNIGDTFTALSNGRYKSCLHRAVVNKYKERKSLAFFLCPKEDKLVRAPNDIVSMDGTKHYPDFTWSHLLHFTQNHYRADQATLPNFTKWLLSSKTTHTS

>GM06G155000 GmGA20ox4

MSPPLMELSASTLVLYPASQPPQEPKNENNGGIVFDSKNLQQMQGEMPKEFLWPSRDLVDTTQEELKEPLVDLAIFKNGDEKAISNAAELVRKACLKHGFFQVINHGVDPDLIDAAYHEIDSIFKLPLSKKMGAKRKPGGVSGYSGAHADRYSSKLPWKETFSFLYDHQSFSNSQIVDYFKSVLGEDLQHTGRVYQKYCEAMKDLSLVIMELLGISLDGDSIMRCNYYPPCNRANLTLGTGPHTDPTSLTILHQDQVGGLEVFVDNKWLAVRPRSEALVINIGDTFMALSNGRYKSCLHRALVNTYRERRSLVYFVCPREDKIVRPPDNLLCRNEERKYPDFTWSNLFEFTQKHYRADVATLQSFIEWQQCSNSNPSNF

>GM07G081700 GmGA20ox5

MLVPHPSMLIAPQNTVENHDNIMEGQKLHFDGSLMPNQSNIPSQFIWPDHEKPCLTPPELQIPPIDLKCFLSADPQALSTVCAELSEACKKHGFFLVVNHGVDSKLIAQAHKLIDDFFCMQLSQKQKAQRKIGEHCGYANSFIGRFSSKLPWKETLSFHYSADKSRKTVEDYFLNVMGEDFKQFGSVFQEYCEAMSKLSLGIMELLGMSLGVGRECFRDFFEGNESVMRLNYYPPCQKPELALGTGPHCDPTSLTILHQDQVEGLQVFVDGRWYSVAPKEDAFVVNIGDTFMALSNGMFKSCLHRAVVNNKIVRKSLAFFLCPNRDKVVTPPKDLISYENSRTYPDFTWPSLLEFTQKHYRSDTKTLDAFSRWLLEKNNPNRGAPTNVPWPTKSIN

>GM09G149200 GmGA20ox6

MAIECITNIQSMSQPQKHHQEHKEDEAPLVFDASLLRHQLNLPKQFIWPDEEKPCMNVPELGVPLIDLGGFLSGDPVATMEAARIVGEACQKHGFFLVVNHGIDANLISNAHSYMDDFFEVPLSQKQRAQRKTGEHCGYASSFTGRFSSKLPWKETLSFQYSAEENSSTIVKDYLCNTLEKEFEQFGRVYQDYCDAMSNLSLGIMELLGMSLGVGKACFREFFEENNSIMRLNYYPPCQKPDLTLGTGPHCDPTSLTILHQDQVGGLQVFVDNEWHSISPNFNAFVVNIGDTFMALSNGRYKSCLHRAVVNSKTTRKSLAFFLCPKGDKVVSPPSELVDDLTPRIYPDFTWPMLLEFTQKHYRADMKTLEAFTNWLLQRKMS

>GM10G241100 GmGA20ox7

MAIDCITSIPMMPQPPTQETTKEQEQQQPLVFDASVLRHQLHIPSQFIWPDEEKACLDEPELFVPFIDLGGFLSGDPVAATEASRLAGEACQKHGFFLVVNHGIDQRLICDAHLYMEHFFGLPLSQKQRAQRKPGEHCGYASSFTGTDKNSSPALVKDYLCSKMGKEFEQFGKVYQDYCDAMSNLSLGIMELLGMSLGVGRACFREFFEENSSIMRLNYYPPCQKPDLTLGTGPHCDPTSLTILHQDQVGGLQVCVDNEWHSIKPDLNAFVVNVGDTFMALSNGRYKSCLHRAVVNSQTTRKSLAFFLCPRSDKVVSPPCELVDNLSPRLYPDFTWPMLLEFTQKHYRADMKTLEAFANWLRKSN

>GM13G035600 GmGA20ox8

MDSGFYLVSSLNHQEVVQSRFFDPSWLQMQPHVPMSFVWPKECLVDANEEFHAPMVDLGGFLRGDDDEATSRAVRLVRKACSSHGCFQVINHGVDSRLIREAYDQMDAFFKLSIRRKVSARKTPGSVWGYSGAHADRFSSKLPWKETLSFPFHDNNELEPVVTRFFNNTLGEDFEQAGVVFQNYCEAMKQLGMKLLELLAISLGVDKLHYKDLFEEGCSVMRCNFYPSCQQPSLALGTGPHCDPTSLTILHQDQVGGLDVFADNTWQTVPPRPDALVVNIGDTFTALSNGRYKSCLHRAVVNKYKERRSLAFFLCPKEDKVVRAPEDIVRRDGTKQYPDFTWSSLLEFTQKYYRADEATLQNFTKWLLSSKQQTL

>GM14G157400 GmGA20ox9

MDSGFCLVSSPNHQEVVQSRFFDPSWLQMQPHVPMSFVWPKECLVNANEEFHAPMVDLGGFLRGDDDDATNRAVRLVRKACSSHGFFQVINHGVDPLLIGEAYDQMDAFFKLPIRRKVSVKKTLGSVWGYSGAHADRFSSKLPWKETLSFPFHDNNELEPPVVTSFFNDTLGGDFEQAGVVFQKYCETMKQLGIKLLELLAISLGVDKLHYNYLFEEGCSVMRCNYYPSCQQPSLALGTGPHCDPTSLTILHQDQVGGLDVFADNTWQTVPPRPDALVINIGDTFMALSNGRYKSCLHRAVVNKYKERRSLAFFLCPKEDKVVSAPEDIVRRDGTKQYPDFTWSRLLEFTQKYYRADEATLQNFTKWLLSSKQQTL

>Cucsa.077290.1 Cucumls sativusGA20ox1

MDSNISQLESQEQAPNEALPFLHASKLTHKTLFVQSKFIWPKGDLVEAYEKLSEPHVDLEGFLKGDKKATLEASKLVRKACLKHGFFQVTNHGVDQNLLATALHEMGPIFNLPFNVKTRASQSHPAKMWGFSTAHSNRFSSKLPWKETFSFGFDHCNSFNNEPSVVDFFSSTLGKEFKEIGVIYEKYCEAMRDLSLALTELLGISLGLERSHFRKFFEDGSSIMRLNSYPICEQGGVALGTGPHCDPTALTILHQDQVGGLEVFANNQWHSVPPTPNALVVNIGDLFMAQCNGEYKSCVHRAVVNNYKKRRSLAFFLCPRKNKVVRPPEKLVADDESRKYPDFSWSELLEFTQKHYRADAATLQNFTKWVVSSRPCGH

>Cucsa.083940.1 Cucumls sativusGA20ox2

MESTTTVGRVAGIILPTMVDKKKKLPREFVWPRGELAGEERGELKEPLIDLGGFRRGEEEPTAEAAAMVRMACMKHGVFQVTNHGVEEELIKAAYEEGEGIFKMPLVKKISVGKKPGRVSGYSGAHADRFSSKLPWKETFSFEYSNDDSQPLHVLHHFKSLFGCDFENTGWVYQRYCEEMTRTALMIMELLAISLGVERYHYRKFFEDGKSIMRCNYYPPCENASLTLGTGPHCDPTSLTILHQDQVGGLEVFANNAWLSVKPRPDALVINIGDTFMALSNGAYKSCLHRAVVNRKRERRSLVFFVCPKDDKVVRPPQDLVGREGPRQYPDFTWSELLEFTQKHYRADVATLQSFVHWLQAKPHPPKIPF

>Cucsa.115530.2 Cucumls sativusGA20ox3

MHVVTSSMAKEAANNDGVPLVFDASVLRHQHKIPKQFIWPDEEKPSEAGCPEMEVPLIDLSGFLSGDKDSVREAVRHVGEACEKHGFFLVVNHGVDLKLIADAHKYMNEFFELPLCEKQRAQRKVGEHCGYASSFTGRFSSKLPWKETLSFRFSADESLDDHVFHYLRTKLGDRFANFGRVYQEYCEAMSGLSLGIMELLGKSLGVEGEYFKNFFKDNDSIMRLNFYPPCQKPHLTLGTGPHCDPTSLTILHQDQVGGLQVFVDNQWRLIAPNFDAFVVNIGDTFMVIKQTTPIIIKIYS

>Cucsa.251180.1 Cucumls sativusGA20ox4

MATECMMNSGRDSTQRVEFGCSEESMVPEKFVWPDEFKPKEGLPELQVPHIDLQKFLSGNQSDIEETIRLVDEACKKHGFFLLVNHGVDMELVKKLHECMEEFFTFPFDVKQKAQRKFGENFGYANSFIGRFSNKLPWKETLSVPYVADHQKSTAHDFIFQIYGNELSHHGKVYQECGEVLSELGLKIVELLGLCLGVPKEKFRKIYEDNESIMRLNYYPPCEKPELTLGTGPHCDPTSITILHQDHVSGLQVYVDDEWHTIPPTTDSFVINIGDTFMALTNGVYKSCLHRAVVNCKESRKSMAFFLNPAADKVVRAPDEVVEKNPPRKFPDFTWPMLLELTQKFYRSDSNTLKAFIPWLEEQQKLAKDTNTAPPL

>orange1.1g016776m Citrus sinensisGA20ox1

MAIDCIKNIPTMLHQPKEEYKDEQKPLVFDASVLKHQTQIPKQFIWPDDEKPCVNAPELQVPLIDLGGFLSDDPVAAKEASRLVGEACRKHGFFLVVNHGVDSSLIADAHRYMDHFFELPLNEKQRARRKLGEHCGYASSFTGRFSSKLPWKETLSFRYSAEKSLSNNIVEDYLLNTMGDEFKQFGRVYQDYCESMSRLSLGIMELLAISLGVDRAHFKEFFEENDSIMRLNYYPPCQKPELTLGTGPHCDPTSLTILHQDQVGGLQVFVENEWRSISPNFEAFVVNIGDTFMALSNGRYKSCLHRAVVNSQTTRKSLAFFLCPKNDKVVSPPSELVDTYSSPRIYPDFTWPMLLEFTQKHYRADMKTLEVFTNWLQQKKQLK

>orange1.1g035820m Citrus sinensisGA20ox2

MESSSSTVLFLPSPVELQGQRDHCGVSLVDSSLWQKQTNLPVEFTWSKGDLVSSHGELMEPVVDLEGFFRGDEVATQRAARIIKDACMSHGFFQVINHGIDASLISAAHDQIDNFFKLPTSRKLKAQRMPGNMWGYSGAHADRFCSKLPWKETLSFGFDDCSLNPMVVNFFNSNIGKDFEQTGIIYQKYCEAMKCLSLGITELLAISLGINDRLHYREFFEEGCSIMRCNNYPTCQASDLALGTGPHCDPTSLTILHQDQVGGLEVFANNRWQSVRPRDDALVINIGDTFTALSNGKYMSCLHRAVVNRFTERRSLAFFLCPREDKVVRPPQDLICRQGTRIYPDFTWSDLLEFTQKHYRSDYATLENFTKWLLTSKPTAKF

>orange1.1g041132m Citrus sinensisGA20ox3

MDCYNSSILPTQEQLLVFDASVLQFQSTIPSQFIWPDGEKPSLELPELVIPPIDLCSFLSGDPFEVDKICQLVDEACKKHGFFLVVNHGVDSMLIKKAHKYMDSFFATQLSKKQKAQRKIGDHCGYSSSFTGRFSNKLPWKETLTFRYRANDENSSTVVEDYFFNVMGQDFRQFGKVYQEYCEAMSSLSLGIMELLGMSLGVGRAHFREFFQGHDSIMRLNYYPPCQKPNLTLGTGPHCDPTSLTILHQDQVGGLQVYVDEQWQSVSPNPDAFVVNIGDTFMALSNGIYKSCLHRAAVNNTTVRKSLAFFLCPKMDKVVTPPNNLVNANNPRIYPDFTWHNLLHFTQNYFRADVKTLNAFSAWLQQHVNI

>Medtr1g081840.1 MtGA20ox1

MDSGLCLVSSPNHQNIVQNNFYDPSWLQKQRNVPMNFVWPKEYLVNANEEFQAPLIDLDGFLKGNEETTNNVAMLISKACSTHGFFQVINHGVDLSLIGEAYDQMDAFFKQPIDKKLIARKIKGSMWGYSGAHADRFSSKLPWKETLSFPFHDNNVFEPSVTNYFDSTLGEDFQQTGVAFQKYCEAMKKLGMKLMEILAISLGLDRFHFKSLFEDGCSIMRCNYYPSCQEPSVALGTGPHCDPTTLTILHQDQVGGLDVFADQKWQTVRPRSDAFVVNIGDTFTALSNGRYKSCLHRAVVNRYKERRSLAFFLCPKEDKMVRPSQDIVSRDGTKQYPDFTWSQLLQFTQNHYRADEATLQNFTKWLLSSKITNTLP

>Medtr1g102070.1 MtGA20ox2

MAIECITSMPQQLLNQETKEQEKPLVFDASVLKHQVNLPTQFIWPDEEQACLNVPELHVPFIDLGGFLSGDPVAAMEASKVVGEACKKHGFFLVVNHGIDEKLISDAHAFMDDFFELPLSQKQRAQRKTGEHCGYASSFTGRFSSKLPWKETLSFQFSADEKSPNLVRDYLCNTMGNEFEKFGEVYQDYCKAMSNLSLGIMEILGMSLGVGKAHFREFFEENSSIMRLNYYPTCQKPELTLGTGPHCDPTSLTILHQDQVGGLQVYVDDQWHSISPHFNAFVVNIGDTFMALSNGRYKSCLHRAVVNSEKTRKSLAFFLCPLSDKVVTPPCELVDNYNPRIYPDFTWSMLLEFTQKHYRADIKTLEAFAKWVQCKST

>Medtr3g088745.1 MtGA20ox3

MDSSQLLCELQIQSHVPKNFIWPKEYLEDAHEELQAPVVDLEGFLKGDNEATQHAAMLINEACLNHGFFQVINHGVDLHLITQAYVQMDTIFKLPLHRKESVYKAPGSMWGYSGAHAHRFSSKLPWKETFSFPYLENAVEPVVTNYFKSTLGDDFEQAGVTFEKYCNAMKELGMNLTELLAISLEVNDRLHYRELFEEGCSIMRCNNYPCCKQPSLVLGTGPHCDPTSITLLHQDQVGGLQVFVDNKWHTVQPLPNAIVVNIGDTFMALSNGRYKSCLHRAVVNQHKQRRSLAFFLCPKEDKVVRPPHDIISRDGTKQYPDFTWSDLLEFTQNYYRADESTLSNFTNWLLSSKSQNFTLINK

>Medtr3g096500.1 MtGA20ox4

MQMALLMNSSTSTHVLYPALKTEEPKTENMVSIFDSNLLQNQVNMPKEFIWPSMDLVNTAQEELKEPLIDLSVMKSGDEEAIASAAELVRKACLKHGFFQVINHGVDQELINDAYCEVDPIFNLPINKKLSAKRVHGGVSGYSGAHADRYSSKLPWKETFSFVYNHQNDSNSQIVNYFKSVLGEDFQQTGWVYQKYCEAMKELSLVIMELLAISLGIDRLHYRRFFEDGDSIMRCNYYPPCKSSNLTLGTGPHSDPTSLTILHQDQVGGLEVFADNKWVAVRPRPEALVINIGDTFMALSNGRYKSCLHRALVNRYRERRSLVFFVCPREDKVVRPPENLLSKNEARKYPDFTWSSLFEFTQKHYRADVATLQSFFQWHSSCNL

>Medtr6g464620.1 MtGA20ox5

MAIECITSTKAMTQSPKQNHGKNEDEESSLVFDASFLRHTINLPKQFIWPDEEKPCMNVPELDVPLIDLKNFLSGDPFAAMEASKIIGEACEKHGFFLVVNHGIDAKLIEHAHSYMDGFFENPLSQKQRAQRKIGEHCGYASSFTGRFSSKLPWKETLSFQFSDEKNSPNIVKDYLCNTLGEDFEEFGEVYQKYCEAMSTLSLGIMELLGMSLGVGKDCFRDFFEENKSIMRLNYYPPCQKPDLTLGTGPHCDPTSLTILHQDQVGGLQVFVDNEWHSIRPNFNAFVVNIGDTFMALSNGRYKSCLHRAVVNNKTTRKSLAFFLCPKGDKVVCPPSELVSDLTPRIYPDFTWPMLLEFTQKYYRADMRTLEAFTKWIQQKSS

>Medtr8g033380.1 MtGA20ox6

MHILNPSMLFAPPNTKEKQCHDTSFLPCQVTNIPSEFIWPEHEKPCLTPPKLQVPPIDLKAFLSGDPKAISNACSQVNDACKKHGFFLVVNHGVDKKLLAQAHKLVDDFFCMQLCEKQKAQRKVGEHCGYANSFIGRFSSKLPWKETLSFRYSDDKSCRTVEDYFVNVMGEDFRQFGSVYQDYCEAMSNLSLGIMELLGMSLGVDKEYFRHFFEANDSVMRLNYYPPCKNPDLALGTGPHCDPTSLTILHQDQVEGLQVLVDGIWHSIVPKEDAFVVNIGDTFMALSNGIFKSCLHRAVVNDTIVRKSLAFFLCPNEEKIVTPPKELINKENPMIYPNFTWPSLLEFTQKHYRADERTLDAFSRWLQEKQLN

>Medtr8g093930.1 MtGA20ox7

MASNLKSENDFKGLIDFKSLHEEAKVPQEFIWSSEDLVETSKEELNVPVIDLEAIFNGDDAALAAAAKIVRETCMEHGFFQVTNHGVDQNLIDATYQEFVSLFKLPLDRKLNAMRNPWGYSGAHAARYSASLPWKETFTFQYKHYDQSETQIVDFFTAALGDDHQHAGWVLQKYCEAMKKLTDVILELLAISLDVDRSYYKKFFEDAETMMRCNSYPPCSGIHAGALGTGPHCDPTSVTILFQDQVGGLEAFVDNKWLGIRPQPNNFVINIGDTFKALTNGVYKSCLHRVLANREKDRKTLAFFLCPKGDKIVRAPENILGRQEPTKYPDFTWKQFFEFTQRKHRADPNTLPDFVSWINSNSSF

>Medtr8g093980.1 MtGA20ox8

MESINTPSLPFFPPPKDQQGENKVQFFDFTLLQKEGNVPKEFIWPSEHWVKSSGENIELPLIDIGVIKSDEAAMANAARIVREACIKHGAFEVTNIGVDSDFINAVLQETYNIFKLPLSKKITAIAKDSGFSVAHAERYTTVLPWKETFTFMYKHNTKNETQVVDVVNSLLGEDFQQSGLVYQKYSDAMNDLTEVIMELLAISLGVDRKHYQRFFEDAESMMRCNFYPPCSANLTGALGNGPHCDPISITILLQDQVGGLEVFADNKWLAVPPKPDTFVINIGDTFMALTNGLYKSCLHRVLVSNELERKSLTFFLNPRGDKTVSPPNELLENEEARKYPDYKWSELYEYTQKTRRVDASTLDSFIAWRHSSETSKF

>Pp3c16_11720V3.1.p PpGA20ox1

MAAFVSDGNSTINAGLLAAILKDSKQLPEMFIESDQNHRPSLESASLEDPDLDIPVLDLSFLAAGNSTQRAEVVAAAAKACQTWGFFQIQNHGIDQKLIVQCEEEAHRMFQLPLDVKERCHRAPGATFGYGANTWINQKVMHWAESFHMQLNPTSNIREMASKLFPEGSSSQQFSSVVEEYMIAVENLASQVLEILTEGLGLEPSCFSQYLRRERMTSMRFNFYPPCPEPSLAIGLRAHTDPHLLTILHQDSVPGLQVQMGDKWITVKPRPDCFVVNIGDLFQILSNTRYKSVLHRAIVNSESRRLSLACFLNPPLNSTVVAPPELITPECPQVYRPFTWLEYLKNAYLYHPTAGNERHERFFLKTMVDVHTVAPSSVH

>Pp3c17_23940V3.2.p PpGA20ox2

MPDIHRPRLHSNAGGAITCEHSSLQNFSEPEMATPWSTVQFSTVSEKFVLPEHQRPTVKHDEYRDLDIPVIDLAPYFEGQRAALQTIIAQVKDSCSNWKFFHVVNHGVDEARLSRIEHQPQQFFKLDSYNEYTSKMQELSELFFHIIITGLGVDSTHFERITKNAGGLFRWNYYPPCPEPHKTIGLKPHTDFNLLTILHTSNVGGLQIWLEGKWSGIRPRCQSSLKLQHETIKCSWVQITVTIHGGLLTNARCEGPLHRAVVNATVPRISHSRTSTCHVPTTVIVAHPALVNENNPSKYIPFTFDAYMKAKQMQLFNTLELFTRKDS

>Pp3c25_4690V3.1.p PpGA20ox3

MLQSATMSACVSEANSTVSSGLHVALLRDSKQLPEMFIESDHNHRPSPDSRCLENPNFDIPVVDISYLTTGDANQRAKAVAAAAKACQTWGFFQVQNHGIDQMLTKRCEVEAHRLFQLPLHVKERCHRAPGTSFGYGANTWINQKVMHWAESFHMQLNPTSNILEMATKLFPDESSSHLFSSTVEEYMKSVENLATQVLELLTEGLGLERNCFSQYLQRERMTSMRFNFYPPCPEPSLAIGLRAHTDPHLLTILHQDSVPGLQVQMGDKWVTVKPRLDCFVVNIGDLFQILSNTRYKSVLHRAFVNSESQRLSLACFLNPPLHATVVAPPELITPECPQVYRPFTWLEYLKNAYLYHPTAGNERHERFYLKSMVDVPAVAPSSVH

>Pp3c3_32410V3.1.p PpGA20ox4

MVALSCESLESVHGSYLEQGGRKSRRVSGWGQKNWFKSIGCCGGATWPSRLLQSSLQSLLSQSSQDLADFVWPEEERPTIPHNDYRRDRELPVIDLSGLQGGSEATKLRLAKRIAAACSEWGFFQLINHGIPVAELDEVQMQTRRFFELPMEHKQRAMTLETANASNVYGYGLNTTGYKYAGRPWIERFQCSWSPKSNLREQAQRLLEGQAFQDFQTAVESFCSGAEKLAKLTLELCALGLGLSPDTFSKHMNPSHSIARLNYYPACPTPDLTLGLGAHTDPYTLTLLHQCQVGGLQVCKDGKWITVKPRRGAYVVNVGDNLQAWTNGRFKSVEHRAVLNDKVPRLSLVFFYAPPLETVITAPDAIIQADGKRRYKSFTWAEYLNFLKRQNFEKGKNERWVEGFAL

>Pp3c4_2230V3.1.p PpGA20ox5

MAAHPISSHSHNAADLAAVLTNTNTTLRNASLLSALALVQQLPAMFVEAEDQRPVLEMDGDPLAFSHLIPIVDMSLLKSSDPAVRASSVADIAAACEKFGFFQVVNHGVDESLIRRCETEAHTMFELPLDVKERVHRPPGTSFGYGANTWVNQTVMHWAESFHMQLHPKSNIREFSGKLFAESDPTKFSSTVEEYMGQIEKLARQLLELLTEGLGLEPTTFNHYVENERMMSMRFNLYPPCPQPELAIGLRAHTDPHLLTILHQDEVAGLQVHIDEKWITVKPRPDCFVVNVGDLFQVLSNTRYKSVLHRAAVNGTSKRLSLACFLNPPLSATVEAPPELITAERPQVYRPFTWGEYLSNAYKFHPAMGGERHERFFLERS

>Pp3c5_23450V3.1.p PpGA20ox6

MNPVATKAFMQETSNDLIQPSTLARGLSDNYSGLRPEVVKSKLLRGATSGLSEFIWPKDQWPSVPHDDFHLAQELPCVDLSDFRTGDAEQRQKTAEVLAKTFSEWGFVLVTNHGVSAEIIKRMQNQALKFFEMPLEQKEKGVVPSSSKYEGFGYGVESGFYYAGKPWIDRFQCRWSPACEIREPMEKVFCPSDAEEFINTIEVYNSRLDKLARKILELCAIGLGIAEDTFTKPFGGTAGDCIARMNYYPPCPLSSLTLGLGAHTDPNLLTILSQCEVGGLQVLRNDTWISVQPKPDMLIVNIGDTFEAWTNGRFRSVEHRAVVNESAARLSMVYFASPPMKAAIEIPGQLITANDPLKFRPSFTWEEYKGHLLQRHAAGNGVKMSKQWLLKPASSSASEDGNRQRF

>Pp3c6_6220V3.3.p PpGA20ox7

MSETRPMNPPPHKSIMHETLNHFRHVGLWDPQSGLIRGQSDRYSGLRPEAVKSNLLRGASSDLSEFIWPKDQWPSVPHDDFHFAKNLPTVDLSGLRAGDAELRFNAARLLAKTFSEWGFVQVKNHGVPAEVIERMQNQARRFFNLPLEQKEKGVATRSSKHEQFGYGVESGFYYAGKPWIDRFQCRWSPVCEIREPVEKVFSPSDAEEFSYSIEVYNSQLDKLATKILELCALGLDLPENTFTKPFKGTAGDCIARMNYYPPCPLSSLTLGLGAHTDPNLLTILSQCKVGGLQVRKNDTWISVEPKPDTLIINIGDTFEAWTNGRFRSVEHRAVVNESEARMSMVYFASPPAKATIEVPEQLVTSKHPLWFRRSFTWEEYKSHLFQMHAGGNGVKISKQWLLKP

>Pp3c8_3050V3.1.p PpGA20ox8

MASNQPPNPFSLIRAASITPAAQSSLQSLLSQGSQDLADFVWPEDERPTIPHNDYRRDRELPVIDLSGLQDGSDETKMKLAKRIAAAGSEWGFFHLINHGIPVADLDEVQLQTRRFFELPMEHKQRAMSLETANANNVYGYGLNTTGYKYAGRPWIERFQCSWSPKSNLREQAQRLLDGQAFHDFQAAVESFCSGAEKLAKLTLELCALGLGLAPDTFSQHMNPSQSIARLNYYPACPTPDLTLGLGAHTDPYTLTFLHQCQVGGLQVCKDGKWITVKPRRGAYVVNVGDNLQAWTNGRFKSVEHRAVLNDKVSRLSLVFFYAPPLDTVISAPQELIQADGKRRYKSFTWGEYLDYLKRQNFEKGKNERWVEGFAL

>Potri.002G151300.1 PtGA20ox1

MVMPPTLPPPRQPLVFDASILQHQANIPSQFIWPDHEKPCLESPKLAIPPIDFGSFLTGDPLAVSKATQLVNEACKKHGFFLVVNHGVDSKLIAKAHEYMNMFFGIQLSEKQRAQRKIGEQYGYASSFTGRFSSKLPWKETLSFRYCADNQSSDIVQEYFLNVMGENFKQFGKVYQEYCEAMNTLSLGIMELLGVSLGVGREYFRDFFEGNDSIMRLNYYPPCQKPDLTLGTGPHCDPTSLTILHQDHVGGLQVFVDEKWHSVSPDPEAFVVNIGDTFTALSNGIFKSCLHRAVVNNVTVRKSLAFFLCPKLDKVVKPPNTLIDSKNPRVYPDFTWPTLSEFTQKHYRADMKTLDVFTTWLQQKNN

>Potri.005G065400.1 PtGA20ox2

MSLLMDSTSSSLLLSPPPFHSKDETDAPVLYSSFLQKQANMPTEFIWPIGDLVHNQDELKEPLIDLDGFLKGDERATADAAELVRTACLNHGFFQVINHGVDIGLIHAAHEEIDKIFKLPLDKKLSTRRKPGDVSGYSGAHAHRYSSKLPWKETFSFGYHGDDDSEPLVVDYFKSVLGENFEHTGWVYQRYCEAMKKVSLVIFELLGISLGVDRLHYRKFFEDGSSIMRCNNYPPCNNSSLTLGTGPHCDPTSLTILHQDQVGGLEVFANNKWQAIRPRPDALVVNIGDTFTALSNGRYQSCLHRAVVNRERERKSLVFFVSPKEEKVVRPPQDLVCREGPRKYPDFTWSDLLEFTQKHYRADVATLQSFIQWLLSAKP

>Potri.005G184200.1 PtGA20ox3

MPRRFVWPKQDLVGAHQELTEPVVDLEGFFRGDEEAIKQASDIIKAACLQHGFFQVINHGVDLNLISLAHDHMHNFFKLPTCDKLRVRRMPGSIWGYSSGHADRYLSKLPWKETLSFGYNENCPNPIGIDFFKSTLGKDFEQTGLVYLKYCEAMKGLSLSIMELLAISLGVDRGYYKSFFEDGCSIMRCNFYPPCQEPGLILGTGPHRDPTSLTILHQDQVGGLEVFSDNVWQTVRPRQSALVINIGDTFMALSNGTYKSCLHRAVVNKYEERRSLAFFLCPKEDKVVRPPLDLVCNQGRRMYPDFTWSDLLHFTQKYYRADDATLQNFTKWIISLKSASL

>Potri.005G184400.1 PtGA20ox4

MDSPLKLQEQSKGFLFDSVLHKQAGFPKEFLWPDLVRAQQELSEPLVDLEGFFKGDEEATKQAANIIKDACSRHGFFQVINHGVDPNVIRDAEDYMDHFFRLPVSEKLKARRMPGSLCGYSGAHADRYASKLPWKETLSFRYHENSSDLVVLDFFKSALGNDFEQTGMVYQKYCEAMMDLSFAILELLAISLGVDRKLYRKFFEDGFSILRCNFYPPCQEPGNTLGTGPHCDSNSITILHQDQVGGLEIFTNKVWQTIPPLQGALIINIGDTFTALSNGKYKSCLHRAMVNQHEQRKSLAFFLSPREDKVVRPPQELVCSEGKRMYPDFTWLNLSRFVQNHYRADDSTLQNFTNWSQSVNL

>Potri.007G103800.1 PtGA20ox5

MSLLMDSTSSSLLLCPPPYLTKDETGALVFDSSFLQKQASLPTEFMWPHGDLVHNEDELKEPMIDLEGFLKGDEVATAQAAELVRTACLNHGFFQVTNHGVDISLIHSAHEEIGKIFKLPLDKKLSVRRKPGDVSGYSGAHAHRYSSKLPWKETFSFGYHGDDDSVPLVVDYFKSVLGKDFEHTGWVYQSYCEAMKKVSLVIFELLAISLGVDRLHYRKFFEDGSSIMRCNYYPPCNNSTLTLGTGPHCDPTSLTILHQDQVGGLQVFSNNKWLAIRPRPDALVVNIGDTFMALSNGRYKSCLHRAVVNRDSERRSLVFFVSPKEEKVVRPPQDLVSREGQRIYPDFTWSDLLEFTQKHYRADVATLQSFIQWLSSSKPSTF

>Potri.012G132400.1 PtGA20ox6

MAIDCIKTMPSKTTHHHHPKDQDQCKDDNKSLVFDAQVLRYQSSIPQQFIWPDHEKPSANAPKLQVPLIDLGDFLSGNPDAAMEASRLVGEACQKHGFFLVVNHGVDKTLIAHALNYMDNFFELPLSEKQKAQRKIGESCGYASSFTGRFSSKLPWKETLSFSYTAEKNSSKHIEQYFHDRMGEDFAKFGRVYQDYCEAMSTLSLGIMELLGMSLGVSRAHFREYFEENDSIMRLNYYPPCQKPELTLGTGPHCDPTSLTILHQDQVGGLQVYVDNEWCSISPNFDAFVVNIGDTFMALSNGRYKSCLHRAVVNSQTPRKSLAFFLCPRNDKKVTPPKELVDTCNPRIYPDFTWPMLLEFTQKHYRADMKTLEMFTNWLQQRNVS

>Potri.014G073700.1 PtGA20ox7

MPIDCFPSNMTIMPPPPPPPEPLVFDASILQHQANLPSQFIWPDHEKPCLEPPELAIPPIDFGSFLTGDPLAVSKLAQQVNEACKKHGFFVIVNHGVDSKLIAKAHEGMNMFFGKQLSEKPVQRKIGEQYGYASSFTGRFSSKLPWKETLSFRYCADNQSSNIVQEYFLNVMGEEFKQFGKVYQEYCEAMNTLSLGIMELLGVSLGVGNEYFRDFFEGNDSIMRLNYYPPCQKPELTLGTGPHCDPTSLTILHQDHVSGLQVFVEEKWHSVTPDPEAFVVNIGDTFMALSNGIFKSCLHRAVVNNVTVRKSLAFFLCPKMDKVVKPPNTLVDYKNPRVYPDFTWLALSEFTQKHYRADMKTLDVFTTWLQQKNNCGGTKNASSC

>Potri.015G134600.1 PtGA20ox8

MAIDCIKTMPSITTPHHHPKDQDQCKDDGKSFVFDAQVLRHQTNIPQQFIWPDHEKPNINAPELQVPLVDLGDFLSGNPVAAVEASRLVGEACQKHGFFLVVNHGVDKTLIAHAHNYMDTFFELPLSEKQKAQRKIGESCGYASSFTGRFSSKLPWKETLSFRYTAEKNSSKHIEEYFHNRMGEDFAEFGSVYQDYCEAMSTLSLGIMELLGMSLGVSREHFREFFDENDSIMRLNYYPPCQKPDLTLGTGPHCDPTSLTILHQDQVGGLQVFVDNEWRSISPNFDAFVVNIGDTFMALSNGIYKSCLHRAVVNSQTPRKSLAFFLCPKNDKMVTPPHELVDTCNPRIYPDFTWPMLLEFTQKHYRADMKTLEVFTNWLHQQSFS

>Sobic.001G005300.1.p SbGA20ox1

MVQQASLGEPLLLQPPPPSLVFDAARLSGLSDIPQQFLWPADESPTPDAAEELAVPLIDLSGDAAEVVRQVRRACDLHGFFQVVNHGIDDALLQEAHRCMDAFFTLPMSDKQRAQRRQGDSCGYASSFTGRFASKLPWKETLSFRYSDDQGDGDVVVDYFVDKLGDAYRHHGEVYGRYCSEMSRLSLELMEVLGESLGVGRRHFRRFFQGNDSIMRLNYYPPCQRPYDTLGTGPHCDPTSLTILHQDDVGGLQVFDAATGPGTGRWRSIRPHPGAFVVNIGDTFMALSNGRYRSCLHRAVVNSRVPRRSLAFFLCPEMDKVVRPPAELVDDANPRAYPDFTWRTLLDFTMRHYRSDMRTLEAFSNWLNHGGHLSSPPPP

>Sobic.002G046500.1.p SbGA20ox2

MAAAGVVVFDAEVLSREERIPAQFVWPAEDRAPSAGGCGVEEIAIPVVDLGEFLRHGDRELPRGVAEACERHGFFQVVNHGVPAALLADAYRCLDAFYARPLADKQRAQRRPGESHGYASSFTGRFRSCLPWKETLSFNCPAGTGGDTQSAAAVVDYFVDVLGEDYRHMGEVYQEYSDEMARLAMDVTEVLAAALGLRRGALRGFFDGGDSIMRLNHYPACRQPQLTLGTGPHRDPTSLTLLHQDDVGGLQVCAGGGGGGEWRAVRPRADAFVVNIGDTFAALTDGRHASCLHRAVVSGDRARRSLAFFFNPPLDRVVRPPDALLLEDEKRRRPRAFPDFTWREFLEFTQKHYRSDASTMDAFVSWIAGGRGDGHGGRQEGK

>Sobic.003G379500.1.p SbGA20ox3

MRPRLPPNVPLSLLANSLSSPLPCTLAHLMVSQERQEPALPLPSNSSSAKRAAASMDASSPAPPLLLRAPTPSPSIDLPAAAGKAAAVFDLRREPKIPAPFLWPHEEARPTSAAELEVPVVDVGVLRNGDRAGLRRAAAQVASACATHGFFQVCGHGVDAALGRAALDGASDFFRLPLADKQRARRVPGTVSGYTSAHADRFASKLPWKETLSFGFHDGAASPVVVDYFTGTLGQDFEPMGRVYQRYCEKMKELSLTIMELLELSLGVERGYYREFFEDSRSIMRCNYYPPCPEPERTLGTGPHCDPTALTILLQDDVGGLEVLVDGEWRPVRPVPGAMVINIGDTFMALSNGRYKSCLHRAVVNQRQERRSLAFFLCPREDRVVRPPASSATPRQYPDFTWADLMRFTQRHYRADTRTLDAFTRWLSHGPVPAQEAAAPCT

>Sobic.009G142400.1.p SbGA20ox4

MPENPHHGSSDRLALVGKQVDRRLLLPVSPAADDGKDDNGAAAVINLWGQLKIPDPFVWSHAETMVSSERELDAPVVDVGAAMRGGDDDCGAGIRRAAELVSGACSSHGLFQVTGHGLDPALARAALDGAAAFFRLPLATKQRASRAPGNVTGYTAAHADRFTANLPWKETLSFGHRDRRTSGSHVVVDYFTSTLGSDFKPLGVVYQNYCNAMKEVSLAIMEVIGVSLGVGRSYYRDFFADGSSIMRCNYYPPCPEPERTLGTGPHCDPSALTVLLQDGDVDGLQVLVDGEWRTVRPRPGALVVSIGDTFMALSNGRYRSCLHRAVVHRERERRSLVFFLCPREDRVVRPPPRLLLAVAAREQQQEQPRRRYPDFTWADLARFTQRHYRADAGTLDAFARWLGAAPTCAAATSAASQSPDKAHETV

>S.moellendorffii 115673.v1.91 SmGA20ox

MNTSCELESYVWPRGDCSLPEHGKFASQQDVPTIDLAGGSKAKVDEIGRACRQSGFFQVVNHGVDHELVNQVHASARQFFEFPSDTKLRAARSAGNSFGFAGKFAGRFKSKCPWKETFSLQYTPNSNIKDYMIKVYTAEQHEQHCARYETYCKAMEKLGRELIELIAQSLELAPDALNSYFDDGFSIFRMNMYPPSEHFPRLLGTGPHTDPCALTILHQDEVGGLQVYNRDETWVTVEPRADAFVISIGDTFQVNRARILLFLLVPLLSLLAAL

>GSVIVT01004790001 VvGA20ox1

MALEIEEHPVESLSSGPKLCVKNFVWSEHEWPLINHDDFADGDDIPTISLEGNLSGKPCQDYDKVCQVMVTACEKWGFFKLVDHGVAIETVENVKVQLNELFDLPMDQKLKGARSTSLPLGYCASNPEYGQNLPWAEILQLLQSPQQVVAFARKVFGDQHQPFSNAMVKYMQALDKLGMKIFEMLAHGMGLPDDFFTKNFEEKEATMIRVNRYPPCPLPEKCLGVGSHSDPHTLTILLQDDVGGLQVLKSDNQWIGIRPVPNSFVINIGDTLEAWTNGRLRSVVHRAVVNKEKHRLSVAYFLSPATSAIIDCPPQLIESSTNLRKYVSFTWGEFRKELLTQKRVVGKTALNRYLISP

>GSVIVT01008782001 VvGA20ox2

MDLGASTLLCPPLELTDKKEHGVSPSDSSFFKKQPNIPMEFIWAKGEVGHAHEELREPVVDLEGFFSGDEVATQHAAMLVRSACLNHGFFQVINHRVDPHLITVAHDHMEAFFKLPWSKKLRAQRKPGSLWGYSGAHADRFSLKLPWKETLSFGFHENGSESVVEDFFKSTLGEEFEQTGLVYQKYCQAMKDLSLVLMELLAISLGVDRLHYRKFFEDGSSIMRCNYYPPCQEPGLAFGTGPHCDPTSLTILHQDQVGGLEVYVNNKWRTIRPRCDALVINLGDTFKALSNGRYKSCLHRAVVNRYKERRSLVFFVCPRENKVYYRADDATLQNFTKWLLSSNPPNHHYIVLDYLVNHDKFLSSRCSFSSIKNISVEWDMAGCNTWSSAIYTSSKMMDRSLSLSSEKDPD

>AC203966.5_FGP005 ZmGA20ox1

MVLAAHDPPPLVFDAARLSGLSDIPQQFIWPADESPTPDSAEELAVPLIDLSGDAAEVVRQVRRACDLHGFFQVVGHGIDAALTAEAHRCMDAFFTLPLPDKQRAQRRQGDSCGYASSFTGRFASKLPWKETLSFRYTDDDDGDKSKDVVASYFVDKLGEGYRHHGEVYGRYCSEMSRLSLELMEVLGESLGVGRRHFRRFFQGNDSIMRLNYYPPCQRPYDTLGTGPHCDPTSLTILHQ

DDVGGLQVFDAATLAWRSIRPRPGAFVVNIGDTFMALSNGRYRSCLHRAVVNSRVARRSLAFFLCPEMDKVVRPPKELVDDANPRAYPDFTWRTLLDFTMRHYRSDMRTLEAFSNWLSTSSNGGQHLLEKK

>GRMZM2G021051_P01 ZmGA20ox2

MAAAAVVFDAEALSREEHIPAQFVWPTEERAPAGGVEEVAIPVVDLGEFLRRGVLPRGVAEACERHGVFQVVNHGVGAALLAEAYRCCDAFYALPLADKQRAQRRHGENHGYASSFTGRFHCCLPWKETLSFNCPAGAGTARAVVGYFVDVLGEDYRHMGEVYQEYCDAMTRLALDVTEVLAAALGLDRGALRGFFEGGDSVMRLNHYPACRQPHLTLGTGPHRDPTSLTLLHQDDVGGLQVRAGGGPWRAVRPRADAFVVNIGDTFAALTDGRHTSCLHRAVVTGGGSRRSLAFFLNPPLDRVVRPPGALLQENKQAGRPRAFPDFTWREFLEFTQKHYRSDAGTMDAFVSWIAGGRRHHGGQEEGN

>GRMZM2G049418_P02 ZmGA20ox3

MRPRLPPNVPSLPSSLSLLANSLSSPVTNTPTRPDSFPAYLQLAHLMVSQERQEPAVPSSSSSSAKRAATSMDASPAPPLLLRAPTPSPSIDLPAGKDKADAAASKAGAAVFDLRREPKIPAPFLWPQEEARPSSAAELEVPMVDVGVLRNGDRAGLRRAAAQVAAACATHGFFQVCGHGVDAALGRAALDGASDFFRLPLAEKQRARRVPGTVSGYTSAHADRFAAKLPWKETLSFGYHDGAASPVVVDYFVGTLGQDFEPMGWVYQRYCEEMKELSLTIMELLELSLGVELRGYYREFFEDSRSIMRCNYYPPCPEPERTLGTGPHCDPTALTILLQDDVGGLEVLVDGEWRPVRPVPGAMVINIGDTFMVTKRKRSLLCFPWPLLSCVYIQLSSLCAVISRILVDLNGQVITARTRRHVM

>GRMZM2G127668_P01 ZmGA20ox4

MTTSCAASSIVSPLAMDMAASGALPVVDLAPFFTDGDEGGASRARATEAVRQACRTHGFFRVVNHGVPAHLMARALELSSAFFALPDDDKARARAPEGSEAPLPAGYARQPAHSADKNEYLLVFGPKLGFNVYPAEPSGFREAVEECYTKLTELGLLVQEVLNECMDLPPGFLTDYNSDRSFDFLAALRYFPATEEEDNGISAHEDGNCITFVIQDGVGGLEVLNDDGDWVPAEPVEGSIIVNLGDVIQVLSNNKLKSATHRVVRKPVHRHSFVFFFNIHGAKWIEPLPEFTAKIGEAARYRGFVYNEYMQLRMRNKTHPPARPEDVVHITHYAI

>GRMZM2G368411_P01 ZmGA20ox5

MDASPTPPLPLRAPTPSIDLPAGKDRADAAANKAAAVFDLRREPKIPEPFLWPHEEARPTSAAELEVPVVDVGVLRNGDGAGLRRAAAQVAAACATHGFFQVCGHGVDAALGRAALDGASDFFRLPLAEKQRARRVPGTVSGYTSAHADRFASKLPWKETLSFGFHDGAAAPVVVDYFTGTLGQDFEPVGRVYQRYCEEMKELSLTIMELLELSLGVERGYYREFFEDSRSIMRCNYYPPCPVPERTLGTGPHCDPTALTILLQDDVGGLEVLVDGEWRPVRPVPGAMVINIGDTFMALSNGRYKSCLHRAVVNRRQERQSLAFFLCPREDRVVRPPASAAPRQYPDFTWADLMRFTQRHYRADTRTLDAFTRWLSHGPAAAAPCT

**GA3ox protein (EC: 1.14.11.15)**

>CSA002905 CsGA3ox1

MPSRVSDAFRTHPVHSPYKHLDFNSLEELPESHAWTSLEKYSSSDSVETITGTKESVPVIDLNDPNAMELVGHACRTWGVFQVTNHGINTSVLNRVESAGKSLFSLPIQQKLKAARSPEGVSGYGVARISPFFPKLMWSEGFTIFGSPLEHACQLWPQDYSNFCDVIEEYEKEMKILARRLMWLMLGSLDISKEDVKWAGPKGDFKGANAAVQLNSYPACPEPDRAMGLAAHTDSTLLTILYQNNTSGLEVHRDGYGWVTVPPIPDTLVVNIGDLLHILSNGLYPSVLHRAMVNRTRHRLSVAYLYGPPPNVQISPLSKLVDLTHPPLYRPVTWTEYLGTKAKYFDKALSSVRLCAFNRFG

>CSA020111 CsGA3ox2

MPSRVPDASHHKHLDFNSVKELPESHAWTSLLGDYPSGPDSCGVGSVPIIDLDDPNAQKLVGHACSTWGVFQVTNHGVKNSLLNSIEAAGKSLFSLPIQQKLKAARSPDGVSGYGVARISSFFPKLMWSEGFTIFGSPLEHARQLWPQDHNQFCHVIEEYEKVMQQLAGRLMWLMLGSLGITKEDIKWAGPKADFKGASAAIQLNSYPACPDPDRAMGLAAHTDSTLLTILHQNNTSGLQVLREGAGWVTVPPQPGTLVVNIGDLLHILSNGLYPSVLHRAMVNRTQHRLSIAYLYGPPTNVQISPLSKLVDPSHPPLYRPVTWTEYLGTKAKHFNKALSSVRLCIPLNGLVDVNDHNRVEVSWRQLPYIKMM

>CSA034282 CsGA3ox3

MTTFSEVYTNNPLQLHHIIPLDFNSLQAVPDSHIWPKSDDDVPTSDEYLSIPIVDLMDPDVVDRVSHACQTWGIFQVTNHGLSPSLLEGVEEETWRLFSLPVPEKMKVLRLPDGATGYGTARIAPFYSKSMWHEGFTIIGGSFVEHAKVLWPNDYKRFCDVIDECQKSMKTLAHKLLHIILKSLNLSEEQESNWATMIHQSETNALQLNSYPTCPDPNQAIGLPPHTDSLLLTILHHSNTSGLQIFRDGGFGWVPVSSFPGALTVNVGDLLHILSNGKFPTVYHRVVVSGTCHRVSMGYFYGLPVDSTVEPLSKVEFPMYKSLTVREYIGIKNECHEKALSLIRI

>AT1G15550 AtGA3ox1

MPAMLTDVFRGHPIHLPHSHIPDFTSLRELPDSYKWTPKDDLLFSAAPSPPATGENIPLIDLDHPDATNQIGHACRTWGAFQISNHGVPLGLLQDIEFLTGSLFGLPVQRKLKSARSETGVSGYGVARIASFFNKQMWSEGFTITGSPLNDFRKLWPQHHLNYCDIVEEYEEHMKKLASKLMWLALNSLGVSEEDIEWASLSSDLNWAQAALQLNHYPVCPEPDRAMGLAAHTDSTLLTILYQNNTAGLQVFRDDLGWVTVPPFPGSLVVNVGDLFHILSNGLFKSVLHRARVNQTRARLSVAFLWGPQSDIKISPVPKLVSPVESPLYQSVTWKEYLRTKATHFNKALSMIRNHREE

>AT1G80340 AtGA3ox2

MSSTLSDVFRSHPIHIPLSNPPDFKSLPDSYTWTPKDDLLFSASASDETLPLIDLSDIHVATLVGHACTTWGAFQITNHGVPSRLLDDIEFLTGSLFRLPVQRKLKAARSENGVSGYGVARIASFFNKKMWSEGFTVIGSPLHDFRKLWPSHHLKYCEIIEEYEEHMQKLAAKLMWFALGSLGVEEKDIQWAGPNSDFQGTQAVIQLNHYPKCPEPDRAMGLAAHTDSTLMTILYQNNTAGLQVFRDDVGWVTAPPVPGSLVVNVGDLLHILTNGIFPSVLHRARVNHVRSRFSMAYLWGPPSDIMISPLPKLVDPLQSPLYPSLTWKQYLATKATHFNQSLSIIRN

>AT4G21690 AtGA3ox3

MSSVTQLFKNNPVNRDRIIPLDFTNTKTLPDSHVWSKPEPETTSGPIPVISLSNPEEHGLLRQACEEWGVFHITDHGVSHSLLHNVDCQMKRLFSLPMHRKILAVRSPDESTGYGVVRISMFYDKLMWSEGFSVMGSSLRRHATLLWPDDHAEFCNVMEEYQKAMDDLSHRLISMLMGSLGLTHEDLGWLVPDKTGSGTDSIQSFLQLNSYPVCPDPHLAMGLAPHTDSSLLTILYQGNIPGLEIESPQEEGSRWIGVEPIEGSLVVIMGDLSHIISNGQFRSTMHRAVVNKTHHRVSAAYFAGPPKNLQIGPLTSDKNHPPIYRRLIWEEYLAAKATHFNKALTLFRC

>AT1G80330 AtGA3ox4

MPSLAEEICIGNLGSLQTLPESFTWKLTAADSLLRPSSAVSFDAVEESIPVIDLSNPDVTTLIGDASKTWGAFQIANHGISQKLLDDIESLSKTLFDMPSERKLEAASSDKGVSGYGEPRISPFFEKKMWSEGFTIADDSYRNHFNTLWPHDHTKYCGIIQEYVDEMEKLASRLLYCILGSLGVTVEDIEWAHKLEKSGSKVGRGAIRLNHYPVCPEPERAMGLAAHTDSTILTILHQSNTGGLQVFREESGWVTVEPAPGVLVVNIGDLFHILSNGKIPSVVHRAKVNHTRSRISIAYLWGGPAGDVQIAPISKLTGPAEPSLYRSITWKEYLQIKYEVFDKAMDAIRVVNPTN

>GM04G071000 GmGA3ox1

MNTLSEAYKAHPLHLSHIIPLDFSSALSLPDSHAWPHCQPNDDASSSSSSSSIPIIDLMDPNAMDLIGHACEKWGAFQLKNHGIPFGVIEDVEEEAKRLFALPTEQKLKALRSPGGATGYGRARISPFFPKFMWHEGFTIIGSPSHDAKKIWPNDYARFCDLMENYEKQMKVLADRLTEMIFNLMDISEEKRKWVGASNISEAVQLNFYPSCPEPNRAMGLAPHTDTSLFTILHQSQITGLQIFKEGKGWVPVHPHPNTLVVHTGDLLHIISNARFRCALHRVTVNRTWERYSVAYFYSPPMDYVVSPLVHSVARFRDVTVKEYIGIKAKNFGEALSFIST

>GM06G072600 GmGA3ox2

MNTTLSEAYKAHPLHLRDIIPLDFSSAHSLPDSHAWSHSQPNDDDYVSFNDDASSSSFIPIIDLMDPNAMEQIGHACEKWGAFQLKNHGIPFCVIEDVEEEAKRLFALPTEQKLKALRSPGGATGYGRARISPFFPKFMWHEGFTIIGSPSHDAKKIWPNDHAGFCDLMENYEKQMKVLAERLTQMMFSLMDISEEKTKWVGASNISGAVQLNFYPSCPEPNRAMGLAPHTDTSLFTILHQSRITGLQIFKEGKEWVPVHPHPNTLVVHTGDLLHIISNARFRSALHRVTVNSTRERYSVAYFYSPPLDYVVSPLVDSVARFRDVTVKEYIGIKAKNFGEALSLIST

>GM07G033800 GmGA3ox3

MPSLSEAFRGHPVYLHHKHSDFNSLQELPDSYSWTQPHDHHLPNYPSNNKTKIFVPVIDLNHPNAPNLIGHACKTWGVFQVVNHDIPMSLFSDIQRASLALFSLPLHQKLKAARSPDGVSGYGRARISSFFPKLMWSECFTILDSPLDLFLKLWPQDYAKYCDIVVEYEAAMKKLAAKLMCLMLASLGITKEDTKWAGPKGEFNGACAALHLNSYPSCPDPDRAMGLAAHTDSTLLTILHQNNVNGLQVLKEGEGWVAVPPLHGGLVINVGDLLHILSNGLYPSVLHRVRVNRTQQRFSVAYLYGPPANVQISPHVKLVGPTRPALYRPVTWNEYLGTKANLFNKALSAVRLSASINGLFDINEDQNNDFQVGFNLDI

>GM08G208300 GmGA3ox4

MPSLSEAFRGHPVYLHQKHHDFNSLQELPDSYAWTQPDDDDHRLTNYPSNNKTKTVVPIIDLNDPNAPNLIGHACKTWGVFQVVNHGIPTSLFSDIQRASLALFSLPLHQKLKAARSPDGVSGYGRARISSFFPKLMWSECFTILDSPLDLFLKLWPQDYAKYCDIVVEYEAAMKKLAAKLMCLMLASLGIPKEDIKWAGPKGEFNGACAALHWNSYPSCPDPDRAMGLAAHTDSTLLTILHQNNVNGLQVLKEGEGWVAVPPLPGGLVINVGDLLHILSNGLYPSVLHRVRVNRTRQRFSVAYLYGPPTNVQISPQVKLVGPTRPVLYRSVTWNEYLGTKAKLFNKALSAVRLSSSINGLFDVNEDKSNDFQVG

>GM13G361700 GmGA3ox5

MPSLSEAYRAHPVHVQHKHPDLNSLQELPESYTWTHHSHDDHTPAASNESVPVIDLNDPNASKLIHHACITWGAYQVVNHAIPMSLLQDIQWVGETLFSLPCHQKQKAARSPDGADGYGLARISSFFPKLMWSEGFTIVGSPLEHFRQLWPQDYHKYCDIVKRYDEAMKKLVGKLMWLMLDSLGITKEDLKWAGSKGQFKKTCAALQLNSYPTCPDPDRAMGLAAHTDSTLLTILYQNNISGLQVHRKGGGWVTVAPVPEGLVINVGDLLHILSNGLYPSVLHRVLVNRIQQRLSVAYLCGPPPNVEICPHAKLVGPNKPPLYKAVTWNEYLGTKAKHFNKALSTVRLCAPS

>GM14G128400 GmGA3ox6

MVTTLSEAYRDHPLHLHHIIPLDFSSLRTIPDSHAWPQSEDGDDDNHGAGSCIPIIDLMDPSAMELIGLACENWGAFQLTNHGIPLSVAEGVEEEAKRLFALPADQKLKALRSAAGATGYGRARISPFFPKHMWHEGFTIMGSPCDDAKKIWHNDCARFCHIMNNYQKQMKALAEKLTHMIFNLLGNISEEQKRWIGSTNLCEAVQLNFYPCCPEPNRAMGLAPHTDTSLLTILHQSQTNGLQIFQEGAGWVPVHPHPGTLFVHTGDILHILSNSWFRCALHRVMVNSMRQRYSAAYFYAPPMDHVVSPLVLDSLPRFRSLTVKEYIGIKAKNLGGALSLISMLNNN

>GM15G012100 GmGA3ox7

MPSLSEAYRSHPVHVQHKHPDLNSLQELPESYTWTHHGHDDHTNSPASNESVPVIDLNDPNASKLIHHACTTWGAYQVLNHGIPMSLLQDIQWVGETLFSLPSHQKHKAARSPDGVDGYGLARISSFFPKLMWSEGFTIVGSPLEHFRQLWPQDYDKYCDFVMQYDEAMKKLVGKLMLLMLDSLGITKEDLKWAGSKGQFEKTCAALQLNSYPTCPDPDRAMGLAAHTDSTLLTILYQNNISGLQVHRKGVGWVTVPPLSGGLVINVGDLLHILSNGLYPSVLHRVLVNRIQRRLSVAYLCGPPPNVEICPHAKLVGPNKPPLYKAVTWNEYLGTKAKHFNKALSTVRLCAPS

>Cucsa.004540.1 Citrus sinensisGA3ox1

MTTTTIPKLTDIYKTHPLDIPTNKLNLDSLQQLPDTYDWIQPDAFPSFRDPNISLSDSVPLIDLSLPNAPHLIGNALKTWGVFQVINHGVPISLLNSMESFTNTLFDLPSPHKLKAARTPDGVTGYGLPRISTFFPKRMWSEGFTIVGSPLEHFQKLWPNEYSKYCDITEEYSREMKSLCGRVIWLALGELGITREDVNWAEANGDFKTSNAVIQLNSYPVCPDPDRAMGLGAHTDTSLLTILYQNNTRGLQVLREGNRWVMVEPVTGALVVQVGDLLHILTNGLYPSSAHQAVVNQNRKRISIAYFFGPSESAEISPLNKLVTPTQPLLYPTVTWTEYLRKKAQLFNNTLSSIRLSAPLTGALDINDHNQVKV

>Cucsa.004550.1 Citrus sinensisGA3ox2

MSDVFKTHDPLNIPTNKSLDSDSDSIPVIDLSLPNAPALMNNAFKTCGAFQVLNHGVPLSLLKSMESFINDLFDLPTSQKLKVVRSPESISGFGLVPLSKIYPKRPWGEGFTIIGNPVDHLQKLWPQDCKKYCDLVEEYNKEMKSLCGKLLWLTLGELGITPEDIYWAGPDGDFKTNNQAIRMNSYPVCPEPDDLIGLPPHSDTSALTILYQTTKGLQVSMEGKGWVDVEPINGALVVQVGDMLHILTNGMYPPSVHQAVVNQTSDRISTAYFFGPPPKGEVSPLKKLVTPTQPLRYPTVTWADYLRKKYVLYEKALPSIRLSAPAPTGLSNGNDQNLVKVG

>Cucsa.004560.1 Citrus sinensisGA3ox3

MATIPKMIEAYKTDPVHALTNKNLDSFEQIPDSHDWVQPNSFPSFTDQSNISDLSSDTDSVPLIDLSLPNAPKLIGNALRTWGVFQAINHGVPISVLNSMESLLNDLFDLPTPQKLKAARSRDGVNGYGRFRISTFFPKSMWSEGFTVSGSPLEHFQILWPHDCTKYCDIIEEYDREMKGLCGRVVWLALGELGITREDVNWAGPNGDFPTSSGVMNLNSYPVCPDPDRAMGIGVHTDSCFLTLLYQNNASGLQVLREGKRWVTVDPVPGALVVQVADLLQILTNGLYSSPFHQAVVNRDRKRLSVAYFFGPPVHAEISPIKKLVNPTQPLLYPTVTWAEYLCKKAELFNDTLPSIRLSTPPTESSDVNDHSQ

>Cucsa.004570.1 Citrus sinensisGA3ox4

MGSNIKITEVFKSHPVHIPIHKNLDFDSLHELPDSYDWIQPDSFPSSSLSNNHLSDSIPLIDLSLPNAPQLIGNAFRTWGAFQVINHGVPISLLHSIESSANSLFSLPPPHKLKAARPSDGISGYGLVRISSFFPKRMWSEGFTIVGSPLEHFRKLWPHDYTRYCDIVEEYDREMKSLCGRLMWVALGELGITREDVNWAGPNGDFKTSNAATQLNSYPVCPDPDRAMGLGAHTDTSLLTIVYQNNTRGLQVLREGNRWVTVEPVPGALVVQVGDLLHILTNGLYPSPVHQAVVNRTRKRLSVAYVFGPPESAEISPLKKLLGPTQPPLYRTVTWTEYLRKKAEHFNNALSSVRLCAPLTGLLDVNDHSQVKVG

>Cucsa.162030.1 Citrus sinensisGA3ox5

MTTLSQTYLDHPLIHHHHIVPLDFDSLRTIPDSHDWFNSSLETPSSSSHHHTNVNVSIPLIDLTDPNAISLIGNACETWGVFQLINHDVPVSLIERAEGETRRLFDLPMTRKLKALRAPGDVTGYGLPRITPFFSKYMWHEGFTIMGPSIGHASQLWPSNYQPFCDVMEEYQRKMKSLAEQITRSIFNYLKISDGANWLHSAGSTEAAACSTALQLNCYPRCPDPTRVMGLAPHTDTFLLTILHQTRTCGLQVFRDGFGWVPVAPVPGALVLNVGDLFHILSNGRFPNVLHRVVVDPTRRRLSMAYFYGPPPDFCVSPLYDPPESPCYRSVMVKDYVRLKAKNLENALSMIRL

>Cucsa.162040.1 Citrus sinensisGA3ox6

MADQEIPPPSQLVPLDFTSVQTVPESHLWPNATELSAAIKLDKRVSLPLIDLVSDGASELLGRACEEWGMFQLINHGISKTLIEEAEEETRRLFTLPATQKMKTLRSPGSLTATGYGMAGISKYHPKLMWHEGFTILGSPINEAKKLWPNDYKRFCDVMEEYQREMKGLGERIIRLILKFLGISEEEMMKLLTSTDESIGKPHMALRLNSYPPCPDPGQVMGLAAHTDTSLCTILHQVGNNGLQIFKDGTGWVPLSPMSGTLIVNIGDLLHILSNGRFPSILHRVMIQENKEHRFSLAYFYDPPGEIYISPYCKPLSDTPQFPLYRSVNVKEYFAIKAKKTGKGLPAIKI

>orange1.1g036286 Cucumls sativusGA3ox1

MPSRLSDAFRAHPVFHQHKHLDFTSLQELPDSYAWTQRDEYPIGDSLISESVPVIDLNDPNALTLVGNACKTWGAFQVINHGIPTNLLDNTESTSRSLFSLPTQQKLKAARSPDGVAGYGLARISSFFSKLMWSEGFTVAGSPLDHFRKLWPQDFCKHCDIIEEYEQEMKKLAGRLMWLVLGSLGITTQDVKWAGPKGHFTDASAALQLNYYPACPDPDRAMGLAEHTDSTLLTILYQNNTSGLQVLKEGTGWVMVPPIPGALVVNVGDLIHILSNGSYPSVLHRAVVNRAKHRLSIAYLYGPPSSVQISPLQKLVGPSHPPLYRPITWSEYLDTKAKHFNKALSSVKSLLS

>orange1.1g043251 Cucumls sativusGA3ox2

MSTTTTLSEAYREHPLHPHHIIPIDFNSVCSLPESHKWPKFDDLYDNKISVPVIDLRDPRVAQLIGRACEKWGVFQLIYHGIPLNVLKDAESEARRLFSLPSRQKLKALRAPAGATGYGIARITPFFDKYMWHEGFTIMGTSIHDHAKQLWPHNHAKFCDVMETYQKKMNILADQLTQLIFKSLDISEEQAEEMNWVGSSSALQLNSYPSCPEPNRAVGLAPHTDTSLITILHENSIAGLQIFKQEVGWVSVKPVDGALVVNVGDLFHILSNARFPNVLHRVSVNQKRQRLSLAYFYNPPTDSTVVPIVKSGQVARYRPVTVKEFISLKAKSPEKALSCIKT

>Medtr1g011580.1 MtGA3ox1

MATTLSEAYRDHPLHLHHIIPLDFSSFRTLPDSHAWPQSNDDGSDNFTSNGCYDNDDEDGSCIPIIDLNDPNAMEQIGLACEKWGAFQLKNHGIPLNFIEEVEEEAKRLFSLPSKEKLKALRSAGGGTGYGRARISPFFPKFMWHEGFTIMGSPSNDAKKIWPNDYKRFCDTMENYQKQMKTLAEKLTNMILNILGISQEQNKWIGSNNHVGAMQLNFYPCCPDPKKAMGLAPHTDTSLFTILHQSQTNGLQLFKEGVGYVPVDPHPNTLVVNTGDILHILSNSRFRCSLHRVVVNDISDRYSVAYFYGPPVDYLVSPCVGDNSLPRFRALTVKDYIGIKAKNLGGALSLISTLLDHDD

>Medtr2g102570.1 MtGA3ox2

MPSLSEAYRAHPVHVNHKHPDFNSLQELPESYTWNHLDDHTLIKEGTTSSIVPVIDLNDPNASKLIGHACKTWGVYQVVNHGIPISLLDEIQWLGQTLFTLPSHQKLKAIRSPDGVSGYGLARISSFFPKLMWSEGFTIVGSPLDHFQQLWPQDYAKHCDTVLQYDEAMKKLAGKLMWLMLDSLGITMEDIKWAGSKAQFDEKACAAMQLNSYPSCPDPDHAMGLAPHTDSTFLTILSQNDISGLQVQREGSGWVTVPPLHGGLVVNVGDLFHILSNGLYTSVLHRVLVNRTRQRFSVAYLYGPPSNVEICPHEKLVGPTQPPLYRSVTWNEYLGTKAKYFNKALSSVSLCAPINGLFDVNDSNKSSVQVG

>Pp3c17_21350V3.1.p PpGA3ox1

MEGSRHEQQKQSLSDLIPVIDLAALNGDHIDEFERRRIITEIAHACKTWGAFQLVNHGIQPHVIERARAKACGVFELPNETRWKAKRSPGSLSGYGNGAVIADAVNNEIASEAITFGYPNSEADVIASIFWPRGNPGFSASIDDYNEESHELALKVVRLMVEGLELHGNLAHFQPYLTEHFGVLRINNYPASEHPTRDIGLPPHIDDTLLTIVHQGCEVEGLQVKKDGQWVTVPPRGDVMVVLVAAVCQVITNDNYKAVLHRAVPNRDKARLSMVYSAYPPANIFITAAPEFVSPAHPPLYKPFTWSEYLSGQVTHILNPIDGLQTVEREGETQNQTSESSESSSKLD

>Pp3c17_7090V3.1.p PpGA3ox2

MTNTFTADSHSARIDLRFEVYLCLSVAARARVSRQVVDMTTERERDHKSEVFSKALRDVIVTDNAVPDAYLWPKQELGPMLGHEEFGEDGIPLIDLGGFEELDNNQRRQIYDRIRGACATYGFFQVVNHGVDLRILDRIQAASKKFFDVPLETKEKLECKLEGDRLLGYGFYKSSKLKTQRRNWSEGLFVDKPHIARVSSTVWPEDTDSQTEFSESVEEYVGALRELGLRLTRLILNSLGVYPETYDKYMPKEPALMRLNHYPPCPDPSKTVGLVPHHDANFFTILHQGDVGGLQVKKDEGWVAVRPYPNAFAVNAGNMLQVISNDICKSVLHKAVVNQDSDRYSIAYFVQAPDWDHIAPLPELVDAAHPVKYRPFTWPEYLESQLANPSNALKNFEL

>Potri.001G176600.1 PtGA3ox1

MPSRSLLADAFRAHPVHVHQKHIDFTSLQEIPDSHKWTQIDDEQHPLVDPLNTESVPVIDLSDPNVLQNIGHACKTWGVLQVTNHGIPTSLLENIESASRSLFSLPIQQKLKAARSPDGVSGYGVARISSFFSKLMWSEGFTIVGSPLEHFRQLWSQDYTKFCDIIEEYKKEMQKLARRLTWLMLGSLGIAKKDLKWAGSTGESKKGSAALQLNYYPACPDPDQAMGLAAHTDSTLLTILYQSNTSGLQVLKEGIGWITVPPIPGGLVVNVGDLLHILSNGLYPSVLHRAVVNRTKHRLSIAFLYGPPSSVEISPLQKLVGPNHPPLYRPVTWNEYLGTKAKYFNKALSSVRICAPLNGLADVNDHNRVRVG

>Potri.003G057400.1 PtGA3ox2

MPSRSLTDAFRSHPVHLHQKHLDFSSLQEIPDSHKWTQLDDIEQQHPSVESFITESVPVIDLLDPNVLQNIGNACKTWGVLQVTNHGIPISLLESVEGVSRSLFSLPVQQKLKAARSPDGVSGYGVARISSFFSKLMWSEGFTIVGSPLEHFRQLWPQDYTKFCDVIEEHEKEMQKLARRLTWLMLGSLGITKKDLNWAGPKGESKEGGAALQLNSYPACPDPDLAMGLAAHTDSTLLTILYQNNTSGLQVLKEGIGWVTVPPIPGGLVVNVGDLLHILSNGLYPSVLHRAVVNRTKHRLSIAYLYGPPSSVQISPIQKLVGPNHPPLYRPITWNEYLVAKAKHFNKALSSVRICAPLNGLVDVNDHNSVKVG

>Potri.006G247700.1 PtGA3ox3

MSTLSEAYRDLPHHPHHIIPLDFDSVRTLPDSHVWQPTSHASESEDRLSIPTVDLKDPDAGKLIGHACEAWGAFQVTNHDIPLDLFHEVESEARRLFSLPTGQKLKALRSPGGATGYGLARISPFFNKYMWHEGFTIMGSSIDHARDLWPNDYRRFCDVMEDYQKKMKELAVTLMHLVLKSLDISEEEISRVVSAGGDSTALQLNSYPLCPDPNRAMGLAPHTDTSLLTILHQSTVNGLQIFKEGVGWVLVSPTNGSLVVNVGDLLHILSNAQFRSVLHRVVMKENQQRLSLAYFYCLPSDFHISPLALNSTQMPLYRSVSVRDYIGIKAKNLEKALSLIRI

>Potri.018G033600.1 PtGA3ox4

MSALSEAYRDHPLHLHHIIPLDFDSVRTVPDSHVWPTSHAFESDDQLSIPTVDLMDPDAVKLVGHACETWGVFQVINHGIPLDIIDEVESEARRLFSLPTGHKLKALRSPGGATGYGLARISPFFSKKMWHEGFTVMGSPVDHARELWPNDYQRFCDVMEDYQKKMKELAITLMHLILKSLDLSEEEISKVVSPGGASTALQLNSYPFCPDPSRVMGLAPHTDTSLLTILYQSTINGLEIFKDGVGWVLVSPTNGSLVVNVGDLLHILSNAQFPSVLHRVVLKEKQQRLSLAYFYSPPTDFDVSPLALNPAQIPLYRSVSVREYIHIKAKNVEKALSLIRI

>Sobic.003G045900.1.p SbGA3ox1

MPTPSHLANPRYFDFRAARRVPETHAWPGLHDHPVVDGGAPGPDAVPVVDLAGAADEPRAAVVAQVARAAEQWGAFLLTGHGVPAELLARVEDRIATMFALPADDKMRAVRGPGDACGYGSPPISSFFSKCMWSEGYTFSPANLRADLRKLWPKAGDDYTSFCDVMEEFHKHMRALADKLLELFLMALGLTDEQVGGVEAERRIAETMTATMHLNWYPRCPDPRRALGLIAHTDSGFFTFVLQSLVPGLQLFRHAPDRWVAVPAVPGAFVVNVGDLFHILTNGRFHSVYHRAVVNRDLDRISLGYFLGPPPHAKVAPLREAVPPGRTPAYRAVTWPEYMGVRKKAFTTGASALKMVALAAAAAAADLDDDAGAGAAAEPVVHQQLLVSS

>Sobic.009G064700.1.p SbGA3ox2

MMPSSSSSASTPAAASGGLFELGSAASVPETHAWPGVNEHPSVESAGRDAVPVVDMGMGGPDDADAAARAVARAAEEWGAFLLVGHGVPRGVAARAEAQVARLFALPAPDKARAARRRRAAAAAAAGYGMPPLALRFSKLMWSEAYTFPAAAVRDEFRRVWPDAGDDYLRFWYVRTPVWYVTRATLPHVDLHLHVHACMRAPSDVMEEYDREMRALGGRLLDLFFMALGGGLTDDDQIAGGETTTTERKIRDNLTAMMHPILYPKCPEPERAMGLAPHTDSGFITLITQSAGVPGLQLLRRGPDRWVTVPAPPGAFVVVLGDLFQVLTNGRYRSALHRAVVNRERDRISVPYFLGPPDGMKVAPLASALLPGRRKAAFRAVTWPEYMELKHKVLGTDTSALEMLQLDEEEM

>GSVIVT01017173001 VvGA3ox1

MPSRISDAFKAHPLHLNHRHLDLNSVQELPDLYAWAGVDENPSGDSLITESVPVIDLTDPNASELVGHACKSWGVFQVTNHGIPGSLLDDIESAGRSLFSLPAQQKLKAARSPDGVAGYGLARISSFFNKLMWYEGFTIFGSPLEHARQLWPQDYTKFCDVTEEFEKEMNQLAERLMWLMLGSLGITKEDLNWAGSKGDFKAALQLNSYPACPEPDRAMGLAAHTDSSLFTILYQNTVSGLQVQREGAGWITVPPLPGALVINVGDLLHILSNGVFPSVVHRALVNRTKHRLSVAYLYGPPAGVPISPVPKLVDSTHPPLYRPVTWSEYLCTKAKHFDKALSLVRLCMPRNGFIDVNDHNGVKVG

>GSVIVT01017178001 VvGA3ox2

MPSELSDAFKSMPANLYKKQLDLNSIQELPDSHAWASLGEHPCVDSLIAESVPVIDLSDPNALTLVGDACKSWGVFQVINHGIPISLLEAIEDASRNLFALPAEQKLKATRPPDGFSGFGQPRIAPFFAKQMWYEGFTVLGSPLELVSKLWPEEYCTKFCEVTEEYDKQMKQLANKLLWLLLGSLGINKEDVEWAGPEGQLEGAHAALQLNSYPACPQPDKAMGLAEHTDSSLLTILYQGSTSGLQVVLEGSGWITVPPLPGALVVNIGDLLHILSNAAFPSVLHRAMVNNSKQRISVAYFYGPPATIPVAPIPKLVDSSHPPVYRSVTWSEFLATKAKHFNKALSLVRMPVPETDSSE

>GSVIVT01035796001 VvGA3ox3

MASTLSQVFRDNPLPLNHIIPLDFTSVHSLPESHVWPAFDGFPFGTTYPGEKFSIPIIDLMDPNAAQLVGHACEKWGAFQLTSHGLPSILTDDVESQTRRLFALPAHEKMKALRLPSGGTGYGQARISPFYPKFMWHEGFTIMGSAVDHARKLWPDDYKGFCDVMEDYQKKMKELAESLLHIFLESLDISKEEYRSTTIQRGREACNTALQLNSYPPCPDPNRAMGLAPHTDSLLFTIVHQSHTSGLQILRDGVGWITVFPLEGALVVNVGDLLHILSNGRYPSVLHRAVVNQAEHRISLAYFYGPPADSLISPLCNLVSSGQQVVAPRYRSVSVKEYVDLKEKHKEKALSLLRL

>GRMZM2G036340_P01 ZmGA3ox1

MPTPSHLNKNPRYLDFRAARRVPESHAWPGLHDHPVVDGGAPGPDAVPVVDLGAADPAPAPAAAVARAAEQWGAFLLTGHGVPADLLARVEDRIATMFALPADDKMRAVRGPGDACGYGSPPISSFFSKCMWSEGYTFSPASLRADLRKLWPKAGDDYTSFCDVMEEFHKHMRALADKLLELFLMALGLTDEQASAVEAERRIAETMTATMHLNWYPRCPDPRRALGLIAHTDSGFFTFVMQSLVPGLQLFRHAPDRWVAVPAVPGAFVVNVGDLFHILTNGRFHSVYHRAVVNRDLDRISLGYFLGPPPHAKVAPLREAVPPGRAPAYRAVTWPEYMGVRKKAFTTGASALKMVALAAAADLDDDGDAAVVHQQQQLVVSS

>GRMZM2G044358_P03 ZmGA3ox2

MQSSSSSASTPAAASGLVFDLGSAAGVPETHAWPGVNEYPSVESAGRDVVPVVDMGVACPDATRALARAADEWGVFLLVGHGVPREVAARAEEQVARLFVLPAPDKARAGRRPGEPTATGYGRPPLALRFSKLMWSEGYTFRAATVREEFRRVWPDGGDDYLRFWCMRAPSDVMEEYDREMRALGGRLLDLFFMALGLTDVQFATGETERRIRETWTATMHPILYPRCPEPERAIGLTAHTDSGFITLIMQSPVPGLQLLRRGPDRWVTVPAPPGALIVMLGDLFQVLTNGRFRSPIHRAVVSRERERISVPYFLCPPEDMTVAPLASALLPGRKAVFRAVTWPEYMEVKHKVFGTDAPALEMLQLQVDEEEQGERAATT

**GA2ox proteins (EC: 1.14.11.13)**

>CSA032124 CsGA2ox1

MVVLAKPAIEQLSAIKTCKLTTTFFSGIPIIDLSKPDSKNLLVKACEEFGFFKVINHGVPTECITKLESEAVSFFSLPLSEKEKAGPPNPFGYGNKSIGSNGDVGWVEYLLLTTNPEFNYQGFESIYGKTPETIRCAVDDYVLAVKRMACEVLELLADGLKIQQRNVFSKLLMDEQSDSVFRLNHYPPYPELQEMNGSNFIGFGEHTDPQIISVLRSNNTSGLQISLKDGNWISVPPDQNSFFINVGDSLQVMTNGRFKSVRHRVIANSLKSRVSMIYFGGPPLSEKIAPLPSLMEGEDSLYKEFTWFEYKKSAYKSRLADNRLGLFEKIAAS

>CSA026961 CsGA2ox2

MVVLSKPAIEQYSVIKTSKPTTFSSSIPLIDLSKPDTKTLLVKACEEFGFFKVINHGVPTELINKLESEALKFFSSPLFEKEKTGPPNPFGYGNKNIGTNGDAGWVEYLLLTTNPEFNYQRFVSIFGKNPEMFRCVVNDYVSAVKKMACEVLELLADGLKIQQRNVLSKLLMDEQSDSVFRLNHYPPCREIQDVNGKHLIGFGEHTDPQIISVLRSNNTCGLQISLRDGNWISVPPDQNSFFINVGDSLQVMTNGRFKSVRHRVVANSLKARISMIYFGGPPLSEKIAPLPSLMEGEDSLYKEFTWFEYKTSAYNSRLADNRLGLFEKIAAS

>CSA004444 CsGA2ox3

MVILSQSVVEDFSHIMTCKPTSGLFTGIPEIDLSDPDAKILIVEACQEFGFFKVVNHGVPIELITRLEAESVKFFNLPQSEKDKAGPSIPFGYGNKRIGPNGDVGWVEYLLFSTNPEFISQMSLSIFQDNPEIFKSAVNDYISNVRDMVCEVLELIADGLKIEPRNVLSKLLRDEKSDSCFRLNHYPPCPELQALSGRNLIGFGEHTDPQIMSAVRSNNTSGLEICLKHGTWVSVPPDQYSFFINVGDSLQVMTNGRFRSVRHRVLADNLKPRVSMIYFGGPPLSEKLTPLCSLMKDGEESLYKEFTWHEYKTSAYKTRLGDNRLGFFLQNASQ

>CsGA2ox4

MVVATPTPIRSEKIREVELPIIDLSAERSEVSKLIVKACEELGFFKVINHGVPEDVIARMEEESFLFFSKSGSEKQRAGPANPYGYGSKNIGFNGDTGEVEYLILNTNFLSISQRSKAISNDPIKFSCAVRDYVEAVRIVACEILELMAEGLWVPDASVFSKLIRDVDEDDSLFRLNHYPPLLLTDSFQDSDTSPSSFHHHLHNHKIGFGEHSDPQILTILRSNDVSGLQISPQQGLWLPVSPYPTTAFCVNVGDLLQAMTNGRFVSVKHRALASSYKSRMSMAYFGAPKLHARITTPPELVTQFRPCLYRPFTWAEYKETTYSLRLSDSRLNLFKVQEGDEIV

>CSA014596 CsGA2ox6

MVVPSPTPSLRTKKTRAVGIPIIDLCLERSVLSQKIVQACEEYGFFKVVNHGVPKEIISSMEKEASDFFAKRSSEKQRAGPPSPFGYGCKNIGFNGDKGELEYLLLQTDPASISERSKAISNNPSKFSCVVNDYIQAVRDLSCEILDLIAEGLWVPDNSVFSKLIRDAHNDSCFRLNYYPPLKHFRELELSPKLNPDTRIGFGEHTDPQILTILRSNDVGGLQICLDDGLWVPVLPDPNVFCVFVGDALKAMTNGRFVSASHRVLVTNTIKPRMSTIYFGAPPLDAYISPIPELVSPQKPSLYKHFTWSEFKKTFYSLRLGDCRLDLFKTLMCSDKTSSC

>CSA013052 CsGA2ox7

MVESNPPLLHDFGQLLLRHPGETCASCHRNARDCVANEEMVILEECELPLIDLGGLRSKDEGERRACAAAICKASSEWGFFQLVNHGISLQLLAEMRREQVKLFNKPFERKATCGLLNNSYRWGNPTATSPKQFSWSEAFHIPLTKISEEACYGEFDSLRKVMQEYAAAMQELANELAGILVKNLGHQKEGVFGESCNGSTCFLRLNRYPPCPWSPEISGLVPHTDSDFLTILHQDDRVGGLQLMKDSKWVAVKPNRDALIVNIGDLFQAWSNDVYKSVEHKVITNAKVERYSIAYFLCPSYDSWIGSCKELSSIYRKFTFGEYRKQIQQDVKMNGHKVGLPRFLR

>CSA034015 CsGA2ox8

MGSSEPPFQDTYKALFDDYVDAESKYEKNDVVIVAECELPMVDLSRLNLGEEEREDCKREIAKASQDWGFFQVVNHGICREILEKMRREQMKVFKRPFCEKINDEFMNFSEGSYRWGTPSATCLKQLSWSEAFHVPLAHISGLGGLTNLSLTMEQFATTVSDLAQKLAEILAEKMGHKSTFFKENCLPNTCYLRMNRYPPCPISSEVFGLMSHTDSDFLTILHQDQIGGLQLVKDGNWIAVKPNQDALIINIGDLFQAWSNGVYKSVQHRVVANKLEERFSTAYFFCPSYDTEIQSCVEPSVYRRFSFREFRQEVQEDVKKFGYKIGLPSFIVQN

>AT1G78440 AtGA2ox1

MAVLSKPVAIPKSGFSLIPVIDMSDPESKHALVKACEDFGFFKVINHGVSAELVSVLEHETVDFFSLPKSEKTQVAGYPFGYGNSKIGRNGDVGWVEYLLMNANHDSGSGPLFPSLLKSPGTFRNALEEYTTSVRKMTFDVLEKITDGLGIKPRNTLSKLVSDQNTDSILRLNHYPPCPLSNKKTNGGKNVIGFGEHTDPQIISVLRSNNTSGLQINLNDGSWISVPPDHTSFFFNVGDSLQVMTNGRFKSVRHRVLANCKKSRVSMIYFAGPSLTQRIAPLTCLIDNEDERLYEEFTWSEYKNSTYNSRLSDNRLQQFERKTIKNLLN

>AT1G30040 AtGA2ox2

MVVLPQPVTLDNHISLIPTYKPVPVLTSHSIPVVNLADPEAKTRIVKACEEFGFFKVVNHGVRPELMTRLEQEAIGFFGLPQSLKNRAGPPEPYGYGNKRIGPNGDVGWIEYLLLNANPQLSSPKTSAVFRQTPQIFRESVEEYMKEIKEVSYKVLEMVAEELGIEPRDTLSKMLRDEKSDSCLRLNHYPAAEEEAEKMVKVGFGEHTDPQIISVLRSNNTAGLQICVKDGSWVAVPPDHSSFFINVGDALQVMTNGRFKSVKHRVLADTRRSRISMIYFGGPPLSQKIAPLPCLVPEQDDWLYKEFTWSQYKSSAYKSKLGDYRLGLFEKQPLLNHKTLV

>AT2G34555 AtGA2ox3

MVIVLQPASFDSNLYVNPKCKPRPVLIPVIDLTDSDAKTQIVKACEEFGFFKVINHGVRPDLLTQLEQEAINFFALHHSLKDKAGPPDPFGYGTKRIGPNGDLGWLEYILLNANLCLESHKTTAIFRHTPAIFREAVEEYIKEMKRMSSKFLEMVEEELKIEPKEKLSRLVKVKESDSCLRMNHYPEKEETPVKEEIGFGEHTDPQLISLLRSNDTEGLQICVKDGTWVDVTPDHSSFFVLVGDTLQVMTNGRFKSVKHRVVTNTKRSRISMIYFAGPPLSEKIAPLSCLVPKQDDCLYNEFTWSQYKLSAYKTKLGDYRLGLFEKRPPFSLSNV

>AT1G47990 AtGA2ox4

MVKGSQKIVAVDQDIPIIDMSQERSQVSMQIVKACESLGFFKVINHGVDQTTISRMEQESINFFAKPAHEKKSVRPVNQPFRYGFRDIGLNGDSGEVEYLLFHTNDPAFRSQLSFSSAVNCYIEAVKQLAREILDLTAEGLHVPPHSFSRLISSVDSDSVLRVNHYPPSDQFFGEANLSDQSVSLTRVGFGEHTDPQILTVLRSNGVGGLQVSNSDGMWVSVSPDPSAFCVNVGDLLQVMTNGRFISVRHRALTYGEESRLSTAYFAGPPLQAKIGPLSAMVMTMNQPRLYQTFTWGEYKKRAYSLRLEDSRLDMFRTCKD

>AT1G02400 AtGA2ox6

MVLPSSTPLQTTGKKTISSPEYNFPVIDFSLNDRSKLSEKIVKACEVNGFFKVINHGVKPEIIKRFEHEGEEFFNKPESDKLRAGPASPFGYGCKNIGFNGDLGELEYLLLHANPTAVADKSETISHDDPFKFSSATNDYIRTVRDLACEIIDLTIENLWGQKSSEVSELIRDVRSDSILRLNHYPPAPYALSGVGQIGFGEHSDPQILTVLRSNDVDGLEICSRDGLWIPIPSDPTCFFVLVGDCLQALTNGRFTSVRHRVLANTAKKPRMSAMYFAAPPLEAKISPLPKMVSPENPRRYNSFTWGDYKKATYSLRLDVPRLEFFKTL

>AT1G50960 AtGA2ox7

MASQPPFKTNFCSIFGSSFPNSTSESNTNTSTIQTSGIKLPVIDLSHLTSGEEVKRKRCVKQMVAAAKEWGFFQIVNHGIPKDVFEMMLLEEKKLFDQPFSVKVRERFSDLSKNSYRWGNPSATSPAQYSVSEAFHIILSEVSRISDDRNNLRTIVETYVQEIARVAQMICEILGKQVNVSSEYFENIFELENSFLRLNKYHPSVFGSEVFGLVPHTDTSFLTILSQDQIGGLELENNGQWISVKPCLEALTVNIGDMFQALSNGVYQSVRHRVISPANIERMSIAFFVCPYLETEIDCFGYPKKYRRFSFREYKEQSEHDVKETGDKVGLSRFLI

>AT4G21200 AtGA2ox8

MDPPFNEIYNNLLYNQITKKDNDVSEIPFSFSVTAVVEEVELPVIDVSRLIDGAEEEREKCKEAIARASREWGFFQVINHGISMDVLEKMRQEQIRVFREPFDKKSKSEKFSAGSYRWGTPSATSIRQLSWSEAFHVPMTDISDNKDFTTLSSTMEKFASESEALAYMLAEVLAEKSGQNSSFFKENCVRNTCYLRMNRYPPCPKPSEVYGLMPHTDSDFLTILYQDQVGGLQLIKDNRWIAVKPNPKALIINIGDLFQAWSNGMYKSVEHRVMTNPKVERFSTAYFMCPSYDAVIECSSDRPAYRNFSFREFRQQVQEDVKKFGFKVGLPRFLNHVY

>GM05G130600 GmGA2ox1

MVVHSHQSALNELFLVKASCKSRFMWVPEVDLTHPEAKTVIVKACQEFGLFKVVNYGVPLELMTHLENEALKFFMQSQCQKDKAGPPDPYGYGSKRIGTNGDLGWVEYLLLNTNPDVISPKTLQLFEQNPEVFRCAVEEYIGAVKKMCCEVLELMADGLEIEPRNVFSRMIRDERSDSCFRMNRYPACPELRVEALSGRNLIGFGEHTDPQIISVLRSNNTSGLQMCLRDGTWASIQPDHTSFFVNVGDLLQVMTNGSFKSVKHRVLANSSMSRLSMIYFGGPPLNEKIAPLPSLVSREEESLYRELTWREYKNAAYKSKLSDNRLSLFDKFADHSDKSPL

>GM08G085400 GmGA2ox2

MVVLSHQSALNELFLVKTCKSTFIGVPEVDLTHPEAKTTIVKACQEFGLFKVVNHGVPLELMTHLENEALKFFMQPQSLKDKAGPPDPYGYGSKRIGTNGDLGWVEYLLLNTNPDVISPKTLQLFEQNPEMFRCGVEEYIGAVKKICCEALELMADGLEIVPRNVFSRMIRDERSDSCFRMNRYPECPELKVEALSGRNLTGFGEHTDPQIISVLRSNNTSGLQICLPDGDGDGTTWASIQPDHTSFFINVGDLLQVMTNGSFKSVKHRVLVDSSMSRLSMIYFGGPPLNEKIAPLPSLVSREEESLYRELTWLEYKNAAYKSKLSDNRLSLFDKSADHSDKSPL

>GM10G123000 GmGA2ox3

MLNESCLVKTCKPSIMFTRVPEVDLSDPEAKSLIIKASKECGFFKVVQHGVAFELITNLENEVLRFFHQPQPQKDKVVPPDPCGYGSRKIGANGDEGWLEYLLINTNPDDPKSLHLFQQNPANFRSAVEDYIGAVKNLCSDVLELMADGLGVEPRNVFSRLTMDERSDCLLRVNRYPVCAELDEFEALSEQYLIGFGEHTDPQIISVLRSNNSHGLQICLRDGTWASIPPDQTSFFVIVGDLLQVMTNGRFKSVKHRVLTDSTISRISIIYFGGPPLNENIAPLPSLVLKEEESLYKELTWQEYKTATFKSRLSDNRLRLFEKFPSE

>GM13G218200 GmGA2ox4

MVVLSQPALNQFFLLKTCKPTPLFSGIPVVDLTDPDAKTHIVKACRDFGFFKLVNHGVPLEFMANLENETLRFFKKPQSDKDRAGPPDPFGYGSKRIGPNGDVGWVEYLLLNTNPDVISPKSQFIFRESPQNFRVVVEEYIRALKNMCYEVLELMAEGLGITQRNALSRLLKDEKSDSCFRLNHYPPCPEVQALNGRNLVGFGEHTDPQIISVLRSNSTSGLQICLTDGTWVSVPPDQTSFFINVGDTLQVMTNGRFKSVKHRVLADPTKSRLSMIYFGGAPLSEKISPLPSLMLKGEESFYKEFTWWEYKKAAYASRLADNRLAPFEKSAAD

>GM13G259400 GmGA2ox5

MVLLSKATTEQYSYIKNCMPTKFSSTIPIVDLSKPDAKTLIVKACEEFGFFKVINHGVSMEAISELEYEAFKFFSMSLNEKEKVGPPNPFGYGSKKIGHNGDVGWIEYLLLNTNQEHNFSVYGKNPEKFRCLLNSYMSSVRKMACEILELMAEGLKIQQKDVFSKLLMDKQSDSIFRVNHYPACPEMTLNDQNLIGFGEHTDPQIISLLRSNNTSGLQIYLRDGNWISVPPDDKSFFINVGDSLQVMTNGRFRSVRHRVLANGFKSRLSMIYFGGPPLSEKIAPLSSLMKGKESLYKEFTWFEYKKSIYGSRLSKNRLEHFERIAAS

>GM13G259500 GmGA2ox6

MVLLSKATTEQYSYIKNYMPTAFSSTIPIVDLSKPDAKTLIVKACEEFGFFKVINHGVPIEAISQLESEAFKFFSMPLNEKEKAGPPKPFGYGSKKIGHNGDVGWVEYLLLNTNQEHNFSFYGKNAEKFRCLLNSYMSSVRKMACEILELMAEGLKIQQKNVFSKLLMDKQSDSVFRVNHYPACPELAVNGQNLIGFGEHTDPQIISLLRSNNTSGLQIFLRDGNWISVPPDHKSFFINVGDSLQVMTNGRFRSVRHRVLANGFKSRLSMIYFGGPPLSEKIAPLPSLMKGKESLYKEFTWFEYKNSTYGSRLADNRLGHFERIVA

>GM15G093900 GmGA2ox7

MVVLSQPALNQFFLLKTCKPTPLFAGIPVVDLTDPDAKTHIVNACRDFGFFKLVNHGVPLQFMANLENETLGFFKKPQSEKDRAGPPDPFGYGSKRIGPNGDVGWVEYLLLNTNPDVISPKSQFIFREGPQNFRAVVEEYIRAVKNMCYEVLELMAEGLGITQRNVLSRLLKDEKSDSCFRLNHYPPCPEVQALNGRNLVGFGEHTDPQIISVLRSNSTSGLQICLTDGTWVSVPPDQTSFFINVGDTLQVMTNGRFKSVKHRVLADPTKSRLSMIYFGGPPLCEKIAPLPSLMLKGEESFYKEFTWWEYKKAAYASRLADNRLGPFEKSAAD

>GM02G010100 GmGA2ox8

MVAPCPTSMMVRTKKTKAMGVPTIDLSLERSKLAELVVKACEEYGFFKVVNHSVPKEVIARLEEEGKEFFSKTSSEKRQAGPANPFGYGCRNIGPNGDMGHLEYLLLHTNPLSISERSKTIAKDPTKFSCVVNDYIEAAKELTCELLDLVAEGLWVQDKFSLSKLIRDVHSDSLLRINQYPPVSLKGTKNWDTSKVEARQIQSQNNNNNNNNNIGFGEHSDPQILTIMRSNNVDGLQISTHDGLWIPVPPDPNEFFVMVGDALQVLTNGRFASVRHRVLTNTTKARMSMMYFAAPPLNRWITPLPMMVTPHNPSLYKPFTWAQYKQAAYSLRLGDARLDLFKIQRQQDTHLAPASP

>GM03G221600 GmGA2ox9

MVVLSPSPTSIKTSYKKKKTMKIPTIDLSMERTELSETVVKACEEYGFFKVINHNVPKEVIARMEEEGAKFFAKPTHEKRRAGPASPFGYGFTNIGPNGDKGDLEYLLLHANPLSVSQRSKTIASDSTKFSCVVNDYVEAVKEVTCEILDLVLEGLGVPEKFALSKLIRDVNSDCVLRINHYPPLNQKLKGNKNSIGFGAHSDPQILTIMRSNDVGGLQIYTREGLWIPIPPDPNQFFVMVGDVFQVLTNGKFMSVRHRALTNTLGARMSMMYFAAPPLDWWITPLAKMVSPPQNPSLYKPFTWDHYKKATYSLRLGDSRLDLFKAQLDTHHVSVSPSQIQC

>GM07G236100 GmGA2ox10

MVLASPNPIRSEGILPSNELIPVVDLTAERSEVAKLIVKACEEYGFFKVINHGISHEVISKTEEAGFSFFEKPVAEKRVAAPAYGCKNIGLNGDMGEVEYLVLGATTHSIAQISKTVSTDPLNFSSTLSAYTEAVRELACEILELIAEGLGVPDTRAFSRFIRDVDSDSVLRLNHYPPIINKDKDKDMSQYSKVGFGEHSDPQIITILRSNDVGGLQISLQDGVWIPVTPDPSAFYVNVGDVLEVMTNGRFVSVRHRAMTNSYKCRMSVAYFGAPPLHATIVAPSVMVTPQRPSLFRPFTWADYKKATYSLRLGDTRIQLFTNRLTTNHHPKV

>GM09G032200 GmGA2ox11

MVAPFPISTRSEKILPIDLPVVDLTAERSMVTKLIVKACEEYGFFNVINHGIPRDTIAEMEETAFDFFAKPMAQKKQLALYGCKNIGFNGDMGEVEYLLLSATPPSISHFKNISNMPSKFSSSVSAYTEGVRELACEILELMAEGLGVPDTWFFSRLIREVDSDSVLRFNHYPPIILNNKDCKDNHNHTKVIGFGEHSDPQILTILRSNDVGGLQISLQDGVWNPVAPDPSAFCVNVGDLLQVMTNGRFVSVRHRAMTNSHKSRMSVAYFGGPPLDACIVAPPVMVTPERPSLLFKPFTWAEYKKVTYSMRLGEHRIDLFRSN

>GM10G010700 GmGA2ox12

MVAPCPTSMMVRTKKTKAMGVPTIDLSMERSKLSELVVKACEEYGFFKVVNHSVQKEVIARLEEEGKEFFSKTSSEKRQAGPANPFGYGCRNIGPNGDMGHLEYLLLHTNPLSISERSKTIANDPTKFSCAVNDYIEAVKELTCEVLDMVEEGLWVQDKFSLSKLIRDVHSDSLLRINQYPPVSLKGTKNWDTSKLEAHQLQSQNNNNNNNNIGFGEHSDPQILTIMRSNNVDGLQISTHDGLWIPVPPDPNEFFVMVGDALQVLTNGRFVSVRHRVLTNTTKARMSMMYFAAPPLNWWITPLPKMVTPHNPSLYKPFTWAQYKQAAYSLRLGDARLDLFKIQRQQDTHLIAPAST

>GM05G081600 GmGA2ox13

MDYEPPFLETYKALVQNHVDDSKNDSSLVERCELPVIDLGKFNYERDECEKEIAEAANKWGFFQVVNHGISQELLKSLEFEQKKLFYQPFVNKSAKFNFSSLSAKTYRWGNPFATNLRQLSWSEAFHFYLSDISWMDQHHSMRSSLEAFASRVFSLAKSLAEILAFNLNTKSNYFRENCLPKSSYIRLNRYPPCPISSKVHGLLPHSDTSFLTIVHQDQVGGLQLMKDGKWVGVKPNPQALVVNIGDFFQAFSNGVYKSIKHRVVASEKVERFSVAFFYCPSEEAVIESHIKPATYRKFTSREYRQQTEKDVKQTGDKVGLSRFLL

>GM11G104800 GmGA2ox14

MIDSNPPLVQHYGALVRNSGEEAKKAKSFNDQNHPLVDACDLPLIDLSGLKSSNERERKACTAAICKAASEWGFFQVVNHGISHDLLRKMREEQVKLFEVPFEKKVTCGLLNNPYRWGTPTATRSKHFSWSEAFHIPLTMISEAASWGEFTSLREAINEFAPAMLEVSRLLASILAQNLGYPEDALEKLCDAGTCFLRLNHYPCCPKSKDEIFGLVPHTDSDFLTILYQDHVGGLQLMKDSKWVAVKPNPDALIVNIGDLFQAWSNDEYKSVEHKVVANNKMERYSIAYFLCPSYSTVINGCKGPSVYRKFTFGEYRHQIQEDVKKIGHKIGLSRFLL

>GM11G003200 GmGA2ox15

MESISDPPFHEAYKILFDKTRNGNNFEEHKELLAVAEECDLPVIDLSRLEESDEVVREECKSQIARASQEWGFFQVVNHGISTEIFSSLRCEQEKVFKQPFEKKTKEDKFLNFSAGSYRWGTPSATCIKQLSWSEAFHIPLTDILGSTGSNSLSWTIEQFATTVSSLAQTLADILAEKMGHKSTFFKENCLPNTCYLRLNRYPPCPIGFGIHGLMPHTDSDFLTILYQDQVGGLQLVKDSKWIAVKPNPDALIINIGDLFQAWSNGVYKSVEHRVMTNPKLERFSMAYFFCPSNDTVIESCREPSFYRKFSFREYRQQVRDDVQKLGSKIGLPRFLTHA

>GM12G213700 GmGA2ox16

MAHEPPFLETYKTLVQKHQGDSRNGYSSCSIVERCDIPLIDLSRLSLEREECMREIAEAAREWGFFQVVNHGISHELLKSLQIEQKKVFYQPFVNKSSTQAKAYRWGNRFATDLRQLSWSEAFHFYLTDISRMDKHETLRSSLEAFAIRMFSVAQSLAEILVCRLNTKSNYFREHCLPESSFIRLNRYPPCPISSKVHGLLPHSDTSFLTIVHQDQVGGLQLLKDGKWVGVKPNPHALVVNIGDLFQALSNGVYKSIKHRVVAAEKVERFSMAFFYCPSEEAVIKSKIKPLMYRKFTLMEYRQQTEKDVKQTGDKVGLSRFLL

>GM12G214000 GmGA2ox17

MDYEPPFLEFYKPLLLQGSHSVDVGISGSGIIRNDKSEWRELPLIDLGQLSLGHVEREDCMREICEAARTWGFFQVVNHGVSQELLQSLRHEQVEVFRTPFARKSRESFLNLPAARSYRWGNPSATNLRQISWSEAFHMFLPDIARMDQHQSLRSTIEAFASVVSPLAESLVQILVQKLNIKFSYFRENCSANTSFLRLNRYPPCPIFHSRVFGLLPHTDSSFLTIVNQDQIGGLQIMKDGNWFGVKPNPQALVVNIGDLLQALSNDIYISAKHRVVAAEKVERFSVAYFYNPSKDALIESHIMPPMYRKFTFGEYRRQIEKDVKETGDKVGLSRFLL

>GM12G029800 GmGA2ox18

MACEREFQMAVLLTLVMIWTPMFVLSDCPLQDFLDHSVSILQFNHINAIQISISTSPPYTLFFHFGAIMIDSNPPLMQHYGALVRNSGEAKEATSFNDQNHPLVDACDLPLIDLSGLKSSNERERRACTAAICKAASEWGFFQVVNHGIRHDLLRKMREEQVKLFEVPFEKKVTCGVLNNPYRWGTPTATRSNQFSWSEAFHIPLTMISEAASWGEFTSLREAINEFAPAMLEVSRLLASILAQNLGYPEDALEKLCDAGACFLRLNHYPCCPKSKDEIFGLVPHTDSDFLTILYQDQVGGLQLMKDSKWVAVKPNPDALIVNIGDLFQAWSNDEYKSVEHKVVANNKMERYSIAYFLCPSYSTVINGCKGPSVYRKFTFGEYRHQIQEDVKKIGHKIGLSRFLL

>GM13G287600 GmGA2ox19

MDYEPPFLEFYKPLLLQGSHRVDVAISGGAIRNDKSEWCELPLIDLGRLSLGGGGEKEECMREISEAARTWGFFQVVNHGVSQELLQSLRHQQVEVFRTPFARKSQESFFNLPARSYRWGNPSATNLGQISWSEAFHMFLPDIARMDQHQSLRSTIEAFASVVAPLAENLMQILAQKLNIKFNYFQENCSANTSFLRLNRYPPCPIFYSRVFGLLSHTDSSFLTIVNQDQIGGLQIMKDGNWVGVKPNPQALVVNIGDLFQALSNDIYISAKHRVVAAEKVERFSVAYFYNPSKDALIESHIMPPMYRKFTFGEYRGQIEKDVKETGDKVGLSRFLL

>GM13G288000 GmGA2ox20

MAYEPPFLETYKTLVQQHLGDSRNEFIVERCDIPLIDLGRLSLEREECMREIAEAAREWGFFQVVNHGISHELLKSLQIEQKKVFYQPFLNKSSTQGKAYRWGNPFATNLRQLSWSEAFHFYLTDISRMDQHETLRSSLEVFAITMFSLAQSLAEILVCKLNTKSNYFREHCLPKSSFIRLNRYPQCPISSKVHGLLPHSDTSFLTIVHQDQVGGLQLLKDGKWVGVKPNPHALVVNIGDLFQALSNGVYKSIKHRVVAAEKVERFSMAFFYSPSEEAIIQSQIKPPIYRKFTLREYRQQTEKDVKQTGDKVGLSRFLL

>Cucsa.047740.1 Cucumls sativusGA2ox1

MVVLSQPPTLDFNQYSLLRSTCKPTASFADIPEIDLSDPNAKFHIVKACEEFGFFKLVNHGVPVELMTKLEDESLCFFKLSKSEKDKARTPHPLGYGSKNIGSNGDKGWIEYLLLNANPLPIFSHDFLCAATEYVTAVKKLSCEVVELIAEGLKMERRNAISKLLKDEKADCCFRVNHYPPCPEMQGLSGLNNMIGFGEHTDPQILSILRSNNSTGLQICLRDGAWVSVPADAAAFFVNVGDVLQVMTNGRFKSVKHKVVVDPNRERVSMIYFGGPPLSEKITPLPEVLKDGEESLYKEFTWWEYKTAAYKSKLADYRLGAFEKSPIC

>Cucsa.054500.1 Cucumls sativusGA2ox2

MVVQSQLVFDHFAPIKSCKRTALFTGIPVINLKDPEAKIQIVKACEDFGFFKLVNHGVSDELIAALESQTLKFFHLPQSEKEKAGPPDPFGYGSKRIGPNGDVGWIEYLLLNTNPQLISQKSISIFHENPEIFRCLVEEYISAMKEMACQVLELMAEGLKIEKSALSKLLKDERSDCCFRLNHYPPCPELEALSGRNLIGFGEHTDPQIISVLRSNNTTGLHICLRDGNWVSVPPDHTSFFINVGDSLQVMTNGRFKSVKHKVLADPIKSRFSMIYFGGPPLNEKIAPLPSLLEEGEKSLYREFTWWDYKSSAYKSRLADCRLTPFEIKNLTPLLT

>Cucsa.291280.1 Cucumls sativusGA2ox3

MVILSKQSIQQLSSNSFMRNSMASAASSSPAFLSEVPLIDLSSPDAKQLIVKACEELGFFKVVNHGVPMEFISTLESESTNFFSLPLSEKQKAAPPSPFGYGNKQIGRNGDVGWVEYILLNTHLESNSDGFLSIFGQDPQKLRSAVNNYISAVRNMACEILELMAEGLKIQQRNVFSKLVMDEESDSVFRVNHYPPCPQIQALKGMNMIGFGEHTDPQIISVLRSNNTSGLQISLADGNWISVPPDQNSFFINVGDSLQVMTNGRFKSVKHRVLTNSLKSRISMIYFGGPPLSEKIAPLPSLMKGEERSLYKEFTWFEYKRSAYNSRLADNRLVHFERIAAS

>Cucsa.372030.1 Cucumls sativusGA2ox4

MDSNPPLFDQYEFLLQSTAHLQPFNNGNLPTNSVMEECQLPLIDLKGLKSSDEKERVACRREIFEASEEWGFFQVINHGIHTELLNRMNKEQIKLFGVPFEKKFTSGILDNSYRWGTPTATHPNQFSWSEAFHIPLTKPINELTDLTSWNGREVMEEVASAMSKVARKLAGVLVESMGQRKELLEDICDESTCFLRLNHYPICPFSGEVSGLVPHTDSDFLTILHQDSGGGLQVMKGSQWLAVKPNPQALVVNIGDLLQSLLPIPQSEHFGEKKE

>Cucsa.059620.1 Cucumls sativusGA2ox5

MAEIEPPLEERYNELKKEELNGRRTIEADEEVIEECEEEIELPVIDLGQLKKGNLEREKCKKEIVEAAMNWGFFQVINHGVAEKVLNAMINEQKKVFNQPFVNKSLSTNFLNLPSTHYRWGNPVAISSSQISWSEAFHIPVLEVSTSHHHITLRTTMEGVVKKLGSLAEKISEILGQSLGIKSNYFKERCEKGKSSFRMNRYPPCPIASQVYGLIPHTDTDYLTILYQPQISGLQLKKSAKWFPVKPNPRALLLNIGDLFQVVSNDIFRSLKHRVVASEGVERYSFAYFYCPSDDVIIESWLKPSIYKHFSYKEYRQQIEKDVEKTGNKVGLSRFFLHNIALPH

>Cucsa.086080.1 Cucumls sativusGA2ox6

MTHTLKHQSSANVAMDPPFHEAYKTLLAERAAAATGAKGASDVVLTVVEEWDLPLVDLERLTAGKVEEVEQCKNDIITASKEWGFFQVVNHGISNQLLAKMRAKQIELFKQPFERKSKEDQFSNFSAGSYRWGTPSATSITQLSWSEAFHVSLSDILGTNGSDDDDLRSTMEEYAGKVSRLAQKLAEILGENLGRSSKFFVENCVPSTCYLRMNRYPPCHVPGQIFGLMPHTDSDFLTILHQDQVGGLELVKDGKWIAVKPNPQALIINIGDLFQVWSNDEYKSVEHRVVTNSKKERYSIAYFLCPWSETVIKSKSEPGVYRRFSFREFRNQVQEDVRKYGYKIGLPRFVL

>orange1.1g019134m Citrus sinensisGA2ox1

MVVQSQPAALDPLSNLIKSCKPTSLFTEIPEVDLTDPAAKTQIVKACEEYGFFKVVNHGVSLELMNKLEAEAVKFFSQSQSEKDKASPPDPFGYGSKRIGPNGDVGWIEYLLLNANPQLTSHKTLAIFQERSQKFRRAVDEYIVAVKKMSYQVMELMADGLNITPRNVMSKLLKDERSDSCFRLNHYPPCPDHQLQALSGRNLIGFGEHTDPQIISVLRSNNTSGLQIQLRDGTWVSVPSDQSSFFLNVGDALQVMTNGRFKSVKHRVLADTNKSRISMIYFGGPPLSEKIAPLPTLLAEEEESLYKEFTWCEYKMSAYKSRLADHRLGLFEKTTAKRSGQISPN

>orange1.1g020152m Citrus sinensisGA2ox2

MVALSTKPAIEQFPYIETCCTTSALFTSTSNSIPLIDLSNPDSKNLLVKACEEFGFFKVINHGVPLESISRLESEAIRFFSLPFSEKEKSGPPSPFGYGNKCIGRNGDVGWVEYLLLNTNQDSNSSLGNNPDQFRFALSEYISAVKRMACEILELMADGLKIQPRNVLSKLLMDEQSDSVFRLNHYPPRPDRISNLIGFGEHTDPQIISVLRSNNTSGLQISLREGSWISVPPDEDSFFINVGDSLQVLTNGRFKSVKHRVLANSLKSRVSMIYFGGPPLSERIAPLPSLMKNPEQSLYKEFTWFEYKRSAYNSRLAENRLMHFERIAAS

>orange1.1g019891m Citrus sinensisGA2ox3

MQLIAMVVASPNPVRNEKILAIDLPMIDLSADRSEVGKLIVRACEEYGFFKVINHGVPEGVIAEMEQESVNFFEKPLAEKQRAGPANPFGYGCKNIGFNGDMGEVEFLLLHTNPLSIAQRSKSISNDPSKFGSAMNNYIRAVRELACEILDLMAEGLWVRDPSYFSKMIRDVENDSFFRINHYPSCCNNNTTRVGFGEHSDPQILTILRSNDVGGLQISPDHGVWIPVAPDPAAFCVNVGDVLQAMTNGRFVSVRHRALSDSSQSRMSMAYFGAPALQARVSAPPEMVTTNRPSLYRPFTWAEYKATAYSLSLGDNRLDLFINCKDDDHKKLLD

>orange1.1g048702m Citrus sinensisGA2ox4

MVVASASAIRTKKTRAVGVPTIDLSLENKELLIKQIVSACEEYGFFKVTNHGVPREIISRLEDEGVDFFDKPVREKQRAGPACPLGYGSRNIGFNGDSGELEYLLLHANPTSIAERSKTISNDPLKFSRAANDYVHAVRGLTCEILDLAAEGLGVRDRFVFSRLIRDVHSDSILRLNHYPPVEDWDPHNSRVGFGDHSDPQILTLLRSNNVGGFQIRLNDGLWVPVPPDPTGFYVLVGDALQVLTNGRFRSVRHRAITNSLNSRMSMVYFGAPPLDAWITSLPETVSLQRPSLYMPFTWGEYKKAVYSLRLGESRLDLFKIRPGDKTGL

>orange1.1g018014m Citrus sinensisGA2ox5

MHPSFKKVQMLESDNFITELNQPLNMATDPPFHQAYKSLIEDTSNNIFGKKLVLVEECELPLIDLSRILHNDNVVDESEREECKEEIARASQQWGFFQVTNHGISKDLLEKMREEQVKVFKQPFDKKSKEDKFMNFPAGSYRWGTPTATCLNQLSWSEAFHIPMADISASAAAFTTLSSTLEQFATTVAGLARKLTAILAEKLGRESTFFQENCLPSTCYLRMNRYPPCPVPSAIHGLMPHTDSDFLTILHQDEVGGLQLVKDGKWIAVKPNPEALIVNIGDLFQAWSNDVYKSVEHRVVTNPSTERFSIAYFFCPSYDTVIQNSEPSNYRKFSFREFRLQVQEDVQKYGHKVGLPRFLISH

>orange1.1g018025m Citrus sinensisGA2ox6

MHPSFKKVQMLESDNFITELNQPLNMATDPPFHQAYKSLIEDTSNNIFGKKLVLVEECELPLIDLSRILHNDNVVDESEREECKEEIARASQQWGFFQVTNHGISKDLLEKMREEQVKVFKQPFDKKSKEDKFMNFPAGSYRWGTPTATCLNQLSWSEAFHIPMADISASAAAFTTLSSTLEQFATTVAGLARKLTAILAEKLGRESTFFQENCLPSTCYLRMNRYPPCPVPSAIHGLMPHTDSDFLTILHQDEVGGLQLVKDGKWIAVKPNPEALIVNIGDLFQAWSNDVYKSVEHRVVTNPSTERFSIAYFFCPSYDTVIQNSEPSNYRKFSFREFRLQVQEDVQKYGHKVGLPRFLISH

>orange1.1g019160m Citrus sinensisGA2ox7

MTVDSNPPLVHHFEKLLRHPDEISTPKRHDCHQQNGIMMEECQLPLIDLIGLNSHDERERAASAKAMCRASSEWGFFQVVNHGISPELLRKMRKEQELLFKTPFERKATCGLLNNSYRWGTPTATCPKQFSWSEAFHIPLTKISDQSCYGEFTSLRLVMTEFAAAMSKLARLLARILAENLSQQGGMLDDICNESTCFLRLNRYPVCPISAEMFGLVPHTDSDFLTILYQDQVGGLQLMKDSKWVAVRPNPDALIVNIGDLFQAWSNDVYKSVEHKVMANGKMERYSVAYFLCPSYDSSIGSCTEPSTYRKFTFEEYRKQVQEDVKQTGHKVGLPRFLQVKNAQL

>orange1.1g019715m Citrus sinensisGA2ox8

MATDPPFHQAYKSLIEDTSNNIFGKKLVLVEECELPLIDLSRILHNDNVVDESEREECKEEIARASQQWGFFQVTNHGISKDLLEKMREEQVKVFKQPFDKKSKEDKFMNFPAGSYRWGTPTATCLNQLSWSEAFHIPMADISASAAAFTTLSSTLEQFATTVAGLARKLTAILAEKLGRESTFFQENCLPSTCYLRMNRYPPCPVPSAIHGLMPHTDSDFLTILHQDEVGGLQLVKDGKWIAVKPNPEALIVNIGDLFQAWSNDVYKSVEHRVVTNPSTERFSIAYFFCPSYDTVIQNSEPSNYRKFSFREFRLQVQEDVQKYGHKVGLPRFLISH

>orange1.1g019949m Citrus sinensisGA2ox9

MDDIEPPFEETYPTLFHNSTARANDKTLFAVDKECELPLIDLARLNFWSFDQWIEEMAEAASQWGFFQVMNHGIPQKVFESMRKEQMKIFHQPFRKKSEQNFMNLSADSYRWGNPKATSLRQFLWSEAFHIPVADISKLEDESINPRSSIGLFATKAANLAERLAEYLAHNLRIKSSYFRDNCLPSSSYLRMNRYPPCPPSFEVLGLIPHTDSDFLTLLYQDHVGGLQLKKDGRWLSVKPNPDVLIVNVGDLFQALSNGVYKSVEHRVVSHPKVERYSVAYFYCPSYEAVIESTENIIKPAIYRKFSFREYKQQIQEDVRATGNKVGLSRFLL

>orange1.1g040559m Citrus sinensisGA2ox10

MDDIEPPFEKTYPTLFHNSTAKANDERLVAVDEECQLPSIDLARLNFRSFDKWIEEMAEAASQWGFFQVMNHGIPQKVLESMRKEQMKIFHQPFRKKSEQNFMNLSADSYRWGNPKATCLKQFLWSEALHIPVTDISRLGDESNNPRSTIGLFSTKAANLAERLAEYLARNLKIKSSYFRENCLPGSSYLRMNRYPPCPPSFEVLGLIPHTDSDFLTILYQDHVGGLQLKKDGRWLSVKPNPDVLIVNVGDLFQALSNGVYKSVEHRVVSHPKVERYSVAYFYCPSYEAVIESTENIIEPAIYRKFSFREYKQQIQEDVRATGNKVGLSRFLL

>Medtr2g070870.1 MtGA2ox1

MVLLSKPSSEQYTYVRNNMQATTFSSSIPLVDLSKPDAKSLIVKACEDFGFFKVINHGIPMEAISQLESEAFKFFSLPLTEKEKAGPANPFGYGNKRIGPNGDVGWVEYLLLNTNQEHNFSLHGKDIDKFRCLLNDYKCAMRNMACEILDLMAEGLKIQPKNVFSKLVMDKQSDSAFRVNHYPACPELAINGENLIGFGEHTDPQIISLLRSNNTSGFQISLRDGSWISVPPDHRSFFINVGDSLQVMTNGRFKSVRHRVLANGINPRLSMIYFGGPPLSEKIAPLPSLMKGNESLYKEFTWFEYKNSTYGTRLADNRLGNYERIAAS

>Medtr8g461330.1 MtGA2ox2

MVVLSKTSLEQYPCIRNLKQTIFSTEIPMVDLSKPDAKNLIVKACEEFGFFKVINHGVSMKCISLLESEAVKFFSMSIDQKEKAGPANPFGYGNKKIGQNGDIGWVEYLLLTNNQDFNQFKLSPAFGKDSDKLRCLLSEYMSSVKKMGCEILELMASGLNIEENNVFSKLLMDKESDCIFRLNHYPPCPPKSNLNNNENENVIGFGEHTDPQIISLLRSNNTSGLQIRLKDKSWISVPSDHNSFFVNVGDSLQVMTNGRFKSVRHRVLANGFKSRLSMIYFGGPSLNEKIAPLPCLIKGNECLLYREFTWFEYKKSAYATRLSDNRLCHFERIKDSS

>Medtr1g086550.1 MtGA2ox3

MVVPSPTSMIRTKKTKAVGIPTIDLSMERSELSELVVKACEEYGFFKVVNHGIPKEVISRLENEGTEFFSKNSTEKLQAGTSTPFGYGCKNIGPNGDKGDLEYLLLHTNPNSISERSKTIAKDHPIKFSCIVTDYIEAVKELACEILELAAEGLWVPDKSSLSKVIKDVHSDSVLRINHYPPVKKLSKDNLDPSKFQNNNNTIGFGEHSDPQILTILRSNNVGGLQISTQHGLWIPVHPDPNEFYVMVGDSLQVLTNGRFVSVRHRVLTNTTKPRMSMMYFAAPPLNWWISPLSKMVTAHNPSLYKPFTWAQYKQAAYALRLGDSRLDQFKLQKQEDNTHYHHDT

>Medtr2g033270.1 MtGA2ox4

MVVASPNSILGERIIPIDLPMIDLSAEKSMVIKLIVKACEEYGFFNVINHGVPHDIISKMEEVGFDFFAKPMEQKKLVALGNPFGYGCKNIGFNGDMGEVEYLLLNANAPSIPNDSSNFSSSVSAYTEAVKELACEILELMAEGLGVPDTSIFSTFITQLDNDSLLRFNHYPPKDCKDRDNSNSYNVGFGEHSDPQILTILRSNDVAGLQISLQHGVWNPVTPDPAAFCVNVGDLLEVMTNGRFVSVRHRAVTNSYKSRMSVAYFGAPPLDACIVAPSVMVTPNRPSLLFKPFTWAEYKKVTYSLRLGDSRIDLFKNCTQIE

>Medtr3g015110.1 MtGA2ox5

MVLASPKPMRNETILPNDLIPIVDLKSERSEVIKQIVKASEEYGFFKVINHGISDGTIEKMEEAGFSFFAKPMSQKKQAAPAYGCKNIGFNGDIGEVEYLLLNANTSSIAQISKTISNDDPHSNFRNVMKTTSLLRYKYEYCKGLKNDILRSNDVSGLQISLQHGLWIPVNPDPEALCVNIGDVLEVFFFSYSYKYVLIERQNKISYLYAFK

>Medtr4g123020.1 MtGA2ox6

MVLASPKPMRNETILPNDLIPIVDLKSERSEVIKQIVKASEEYGFFKVINHGISDGTIEKMEEAGFSFFAKPMSQKKQAAPAYGCKNIGFNGDIGEVEYLLLNANTSSIAQISKTISNDDPHSNFRCRVSEYTEAVKEVACEILELMAEGLGVPDTKVFSSLIKDIDSDSVLRLNHYPPTLNKDKSHSNNVGFGEHSDPQILTILRSNDVSGLQISLQHGLWIPVNPDPEALCINIGDVLEVMTNGRFVSVRHRAMTNSYKSRMSMAYFGAPPLNASIVAPPVLVTPTRPSLFRPFTWADYKKATYSLRLGDTRIQLFRANIA

>Medtr2g083000.1 MtGA2ox7

MDYEPPFLQTYMSLLQGTNDLISDLSDVEKREIPLIDLKRLKLDQLEREECMKEITEAARKWGFFQVVNHGVSQEVLKNMQFEEKEVFRTPFGIKSQENFLNLPSRTYRWGNASAINPKQLMWSEALHIFLPDIEKMDQHKSLRSSIESFVKVVTPLAENLVQILAQELNINFSYFQQNCSANTSYLRLNRYPPCPFPSKVIGLLPHADTSFITIVHQDHIGGLQLMKDGKWISVKPNSEALIVNVGDLFQALSNGLYTSVGHRVVAAEKVERFSLAYFYGPSIDAVIESYATPPLYRKFTFGEYKEQTMKDLKEGGDKVGISRFLL

>Medtr2g083030.1 MtGA2ox8

MDFEPPFLKIYNTLLEKNLGDNSENDLYSKVEGSEELPLIDLEKLNLEDPKREECMKEISEAASKWGFFQIINHGISNEILNKMISEQKKLFYQPFVNKLSAETVFNLSPKTYRWGNPCATNLRQLSWSEAFHFALTDIPNMDQHITLRSSLEDFATRMDTLAENLVEILALKVNMKSNHFQENYLPKSSFIRLNRYPPCPISSEVFGLLAHCDTSFLTILYQDSVGGLQLMKDGKWVDVKPNPSALVVNIGDLFQALSNDVYKSIKHRVVAAEEVERFSTAFFYCPFNDAVIQSENKPAVYKKFTLREYRQQTLNDVKETGDKVGLSRFVL

>Medtr4g074130.1 MtGA2ox9

MIDSNPPLLNHYGALLRNSAEPQKAKSSNGQDNTVVECELPLIDLNGLKSCNVSERLACTAAICKAASEWGFFQVINHGINPDLLRNMREEQMKLFRVPFEKKVTCGLLNNPYRWGTPSATSSNHFSWSEAFHIPLTMISEAACWGEFNTLREAINEFAAAMLEVSRLLAGILAENLGHPTDAVEKLCDASTCFLRLNHYPSCPKSKEEIFGLVPHTDSDFLTILYQDQVGGLQLMKDSKWVAVKPNPEALIVNIGDLFQAWSNDEYKSVEHKVVANDKVERYSIAYFLCPSYTTMISGCKEPSTYKNFTFGEYRHQIQEDVKKIGHKVGLSKFLRKDTYTTTMA

>Medtr5g005570.1 MtGA2ox10

MGLIDSDPPFEETYKNLFNKDQNIINDELMVDNECELPVIDLSRLNDDDEVAREECKSMIANASQEWGFLQVVNHGISSDILTRLRCEQKKVFKEPFDKKTKEDKFLNFSAGSYRWGTPTATCIKQLSWSEAFHIPLTDILGSNTHLSSIIEQFATTVSNLAQILANILAEKLGHQSSFFKENCLPNTCYLRLNRYPPCPIDFRIHGLMPHTDSDFLTILYQDQVGGLQLVKDGKWVAVKPNPDALIINIGDLFQAWSNGVYKSVEHRVVTNPRVERFSVAYFLCPSNDTMIESCKEPSIYRKFSFKEYRQQVRDDVQKLGSKIGLPRFIIN

>Medtr7g047670.1 MtGA2ox11

MDPVSDPPFEEAYKILLNKTKNNRNNVPNDNKFPVVEECELPVIDLSRLEDDNEMVREACKYEIARASQEWGFFQVINHGIPNDIFSRLKCEQEKVFKLPFDKKTKEDKFLQFSSGSYRWGTPSATCVGQLSWSEAFHIPLKDVLESNAQPNTLRSTIEQFAIISSNLAQTLAHILAEKMGHESTYFKENCLPNTCYLRLNRYPPCPIASEIHGLMPHTDSDFLTILYQDQVGGLQLVKDKKWIAVKPNPSALIINIGDLFQAWSNGLYKSVEHRVVTNPKVERFSMAYFLCPSNESVIESCKKPSLYKEFSFQEYRQQVRDDVQKLGTKIGLPRFLLF

>Medtr7g451860.1 MtGA2ox12

MDPVSDPPFEEAYKILLNKTKNNRNNVPNDNKFPVVEECELPVIDLSRLEDDNEMVREACKYEIARASQEWGFFQVINHGIPNDIFSRLKCEQEKVFKLPFDKKTKEDKFLQFSSGSYRWGTPSATCVGQLSWSEAFHIPLKDVLESNAQPNTLRSTIEQFAIISSNLAQTLAHILAEKMGHESTYFKENCLPNTCYLRLNRYPPCPIASEIHGLMPHTDSDFLTILYQDQVGGLQLVKDKKWIAVKPNPSALIINIGDLFQAWSNGLYKSVEHRVVTNPKVERFSMAYFLCPSNESVIESCKKPSLYKEFSFQEYRQQVRDDVQKLGTKIGLPRFLLF

>Pp3c16_4060V3.1.p PpGA2ox1

MASTGEVSQGEEDSEDEVQKKPEIPLYSMQALWDGKMSLKDFEVKGEEKLTLPHDVFEHDEALPVIDISALLGTDKKIRDENMAAMLDAAKTWGFFKIRNHGVPLEVVKKVESNVKNFFALPMEKKLMVKAINFAFGYVGGSPVSWRYKWWLEGLHMKVNYQAIRDMVNLVWSDDKDFAEEFISDLTSYFDTMRYLSRLIVECLTEGLSLPRDTYTKLETENAICNARVNHYPACPDPSKVFGIPGHTDPQMLSILYQDDVGGLQVLKDGKWIGIRPDDSTLVCNLGDTFQVITNGILHSAGHRVAVNATRSRYATIYFYGIDNVIPLCVPPQLVTKDRPLKYRPFTVHEYRAHCVEKQVPIDGVKFLEIQPQSNNPSTSQSKSPQ

>Pp3c16_4070V3.1.p PpGA2ox2

MTAGAEKQPEDGSHEKQNVHLDSTKQAAASPVYTMQAFFDGKMKVEDFARSEQERPTVPHDIFDDTLPVVDIAAIKDGTAEERRANVVKMLKAAKSWGFFKISNHEIPLQVVLSNLQLIFVTADHACDEAVKRVKKNGKQFFALPMERKLPVRAPDFVFGYTGGSPVNWKSKWWLEALMIKVTDEALDDMIGQVFPDEKDFGVRFKEDLKAFFSPMHELSRFIVEELTEALGLERDTFTRLETPGSNCTGRMNHYPVCPNPDSVLGIPPHADTQLLGILHQDDTGGLQVLKDGEWVGIQPDDSTFVVNIGDTFQAITNGILRSAAHRAVVNAKKDRYSTIYFYGIDNSITLTVPPKLITEDRPLKYRPFTVREHRKYIVDNEVPINAVQHLEINPEATALFLEKLFG

>Pp3c27_4790V3.1.p PpGA2ox3

MAAGAAVTQSEDSGHDKPNAQIQLEKPVDTSPLYTMQAFFDGKMKVEDFARSEQERPSVPHYVFDDTLPVIDIAAIKERSSEEREANVAKMLMAARSWGFFRIINHDVPVELVKQVEANGKQFFALPMERKLSVRAPDFVFGYTGGSPIKWKSKWWLEGLMIKVTDEALDDMVNQVYPHEKEFAAQFKKDLKSFFGPMHELSRFIVEELTEGLGLERDTYTRLETPNSNCTGRMNHYPVCSDPDSVLGIPGHADTQMLAILYQDDVGGLQVLKDGEWVGIRPDDNSFIVNIGDTFQAITNGILHSASHRAVVNSKKDRYSTVYFYGIDNSITLTVPPGLITEDRPLKYRPFTIMEHRKYIVDNEVPLDAVRHLQINPEA

>Pp3c27_4820V3.1.p PpGA2ox4

MGSSELLIHGQDNRGGGEVEKKSEIPLYTMQALWDGKISLKDFEVVGMDKPIIPHDVFEPDEALPVIDVEALLGADNEARLENMVRMLEAAKTWGFFKIRNHGVSLEVVKKVEANVKKFFALPMEKKLLVKANNFSFGYVGGSPVSWKYKWWLEGLHMKVQDEAIKNIVDLVWSDDEEFSTEFTADLTNYFAIMRDLSRLIVECLTEGLGLPRDTYTKLETPNAICNARVNHYPACPDPSSVFGIPGHTDPQMLSILYQDDVGGLQVLKDGKWIGIRPDDSTFVVNLGDTFQVISNGILHSAAHRVAVNTTRSRYATIYFYGIDNEIPLFIPPQLVTKERPLKYRPFTVNEYREHLVIKQVPMDGVKYLEMEPEIQDAASPSNSSQ

>Pp3c5_2410V3.1.p PpGA2ox5

MGVSEMNRTEDCVDADIVMKEPEVETATIPTYTMQALWDKKLSLKDFEVSGEDKPTLPYDVFEQDEVLPIIDLQALLGNDKDARYVNLARMLEAAKTWGFFKIRNHGVALETVKKVEQNVKKFFALPMEKKLQVKAVNFVFGYIGGSPVSWKNKWWLESLHMKVQEEAIRKMVALVWSDNPKFADEFMSALTRYYSSMRELSRLIVECLTEGLGLPPNTYTKLETPDAICNARVNHYPACPDPSKVFGIPSHTDPQMLSILYQDDVGGLQVLRNGKWIGIRPDDSTLVVNLGDTFMAITNGILHSAVHRVALNTTRSRYSTIYFYGVDNAAPLSVPPELITEDRPLLYRPFTVNEYRAILTEHQVPDDGIKFLRIQPGVTATARKKMFRR

>Pp3c6_5720V3.1.p PpGA2ox6

MAVSMSDANTSMHAGLVSPLPQNSTQLPQEFVEADQKHLSNAELESTEDPELDFPVLDLSLLSGDKKHHDEVITAAAEACQNWGFFQIQNHGIDQRLIEKCKEEALRMFQLPLEAKKRCDRPPGTSFGYGSNTWVNQKVQHWAESFHLQLKPMSNVPAMASKLFPDQTSSQQFSSVVEEYMETVQNLAIQVVEILTEGLGLPPAYFSQHLQRERMVSMRLNFYPPCPEPSKAIGLRAHTDPHLITILHQDTVRGLQVQVAEKWVTVKPRPDCFVVNIGDIFQILSNTRYKSGLHRAVVNGQFQRLSMACFLNLPLDCVVAAPPELITTDCPQKYRPFLWLEYLKHAYLYHPITGNDRHEKFFLKPSGDIPVAASSVH

>Pp3c13_6170V3.1.p PpGA2ox7

MTLPPERIIQNSGLLQTRGLVTHMPNEMDLALPSHNMFSASSHTLPTIDLHSAHLGEQVVSACRDWGFFQVHNHGIPPELLNRLRAHAHCFFELPLQQKERVAACKSNNFYGYGVSKARTYFPNDWMEAFDMEWTPISRVRRHVQQVGLAHARYEDFCGAVEDYASKTEKLAVRLTEQVALGLGLDATAFSRHFEESTTSTVRMNYYPPHPNPSRTLGISPHSDFNIFTILLHDTVPGLQVLKDGKWITVKPSPDTLVINVGDTFQAWCNGRIKSVMHRALVNATEPRLTVVHFFGPHPDTMIVPPAALVDNDNPLRYRSFAYKEMVEQIMHIRGKSVFESPLTSFLL

>Pp3c16_9590V3.1.p PpGA2ox8

MPVAQPLHPSHHKSLMHEAPNHFRTLAPRNGLHRGQSDNYSGLRPEAVKSNLLRGASADLSEFIWPKDQWPSVSHNDFHMAQELPTVDLSDLHHGSEESREATAKLLVKTFSEWGFVQVINHGVPTEVIEKMQNQARNFFDLPLEQKEKGVASSTSKHEGFGYGVESGFYYAGKPWIDRFQCRWSPVCEIREPVEKVFSPTDAEEFSTSIEDYNGRLDKLAMQILELTAQGLGLPSDTFIKPFNGTAGDCIARMNYYPPCPLSSLTLGLGAHTDPNLLTILSQCKVGGLQVCKNGTWISVKPKPDTLIINIGDTFEAWTNGRFCSVEHRAVVNESEARMSLVYFASPPSKSVIQIPEQLITAKHPLRFRPSFTWEEYKSHLFKRHVGGNGVKTSKEWLRLPAPTSHT

>Pp3c25_8200V3.1.p PpGA2ox9

MPVAQPLHMSHHKSLMRGVPNHYRTMALKNSLHRGQSDNYHRLRPEAVKSNLLRGASADLSEFIWPKDQWPSLAHNDFQLAQELPTVDLSGLLFGSEEERDKTADLLVKMFSEWGFVQVINHGVPTEVTQKMMIQARNFFDLPLEQKEKGVASSSSKHEGFGYGVESGFYYAGKPWIDRFQCRWSPVCEIREPVEKVFSPSDAEEFSNSIESYNSHLDKLAMQILELCARGLGLPHDTFTKPFNGTAGDCIARMNYYPPCPLSSLTLGLGAHTDPNLLTILSQCKVGGLQVCKNGTWISVKPKPDTLVINIGDTFEAWTNGRFQSVEHRAVVNETEARMSLVYFSSPPTKSLIQIPEQLITAEHPLRFNPSFTWEEYKMYLFKKHVEGNGVKVAKDWLRRPATTSQN

>Pp3c26_14320V3.1.p PpGA2ox10

MTLSDAANHILLNSGLLQSRTLGEFVLQDSDQPLPAQNLFSSSDALPTIDLYCAGAAEQLAAACRDWGFFQIQNHGIPLELLNRLRAHSHSFFELPLEQKERAAACSANSFYGYGIAKGRTYSPNAWMEAFHMEWTPASQVRRHMERIGIPQPQFEGFCEAVEQYASQAEKLAVQLMELVALGLGLDATTFSRHFAGSSTSTVRMNYYPPCPQPSRTLGISPHSDFNIFTILLQDTVAGLQVLKDNDEWVTVKPNPNALVVNVGDTLHAWSNGRIKSVMHRALVNTTEPRLSVVYFFGPHPDTKIDPIPALVSSDCPLRYRSFVYKQFVEQILQNRGKKVFESPLNSLLL

>Potri.001G378400.1 PtGA2ox1

MVLISKPALEQFSFIRNRKPTTVFSGIPLIDLSKPDSKHLLVEACEEFGFFKVINHGVPMEFISKLESEAVNFFSLPLSEKEKVGPPSPFGYGNKSIGQNGDVGWVEYLLLTTNQESISQRFSSVFGDNPEKFRCALNDYVSAVKKMACEILEMMADGLKLQQRNVFSKLLMDEQSDSVFRLNHYPPCPEIEALTDQNMIGFGEHTDPQIISVLRSNNTSGLQISLSDGSWISVPPDQNSFFVNVGDSLQVMTNGRFKSVRHRVLTNSMKARVSMIYFGGPPLSEKIAPLPSLIKGKESLYKEFTWFEYKRSAYSSRLADNRLVLFERIAAS

>Potri.004G065000.1 PtGA2ox2

MVVLSQLALEPFSVIKTCKPIGLFSEIPVIDLTDPHAKTLIIKACEEFGFFKLVNHGVPMEVMTKLEALATNFFNLPQPEKDKAGPPNPFGYGNKKIGPNGDVGWVEYLLLNTNPQISSQKTSIFQENPQIFRSAVEDYILAVKRMAFEVLELMADGLEIESRNVFSRLLRDDKSDSCFRLNHYPPCSELQALSGGNLIGFGEHTDPQIISVLRSNNTSGLQICLKEGTWVSVPPDQTSFFINVGDALQVMTNGRFRSVKHRVLADPLKPRISMIFFGGPPLSEKIAPLPSLMAERGGSLYKEFTWFEYKRSAYKSRLADYRLGLFEKTAGQ

>Potri.011G095600.1 PtGA2ox3

MVLVPKPALQQFSFIRNIKPTTFFSGIPLIDLSKPDSKHLLVKACEEFGFFKVVNHGVPLEFISKLESEAVKFFSLPLSEKEKASPPNPFGYGKKSIGQNGDVGWVEYLLLTTNQESVSQRLSSVFGDNPEKFRCALNDYVSAVKKMACEILEMMADGLKIQNRNVFSKLLMDEQSDSVFRLNHYPPCPEIQALKDHNMIGFGEHTDPQIISVLRSNNTSGLQISLNDGSWISVPPDPSSFFINVGDSLQVMTNGRFKSVRHRVLANSIKARISMIYFGGPPLREKIAPLPSLMEGEESLYREFTWFEYKRSAYNSRLADNKLVLFERITAS

>Potri.002G191900.1 PtGA2ox4

MVVPSPTPARTKKTKAFGIPTVDLSLDRSNVSKLIVRACEEYGFFKVTNHGVSKEVVTRMEEEAAHFFSKPATEKHRAGPASPFGYGCKNIGCNGDMGELEYLLLHANPLSVFERSKTISNDPSEFSCVVNDYIQAVKQLACEILDLTAEGLWVPDKRAFSRLIRDVHSDSVLRLNHYPAFEEIMDWDPSPKTIGFGEHSDPQILTILRSNDVGGLQIYLRDGLWVPVPPDPTGFYVIVGDAFQVLTNGRFESVRHRVLASSGKPRMSMMYFGAPPLNAWISPPPQLVLPQNPSLYKPFTWSEFKKAAYSLRLRDTRLDLFKIHATEKFAS

>Potri.008G101600.1 PtGA2ox5

MVVASPTKLHSEEHLAIELPTVDLSGDRSMVSNLIVKACEEYGFFKVKNHGVPHDIIAQMEKESFNFFAKPFDEKQKVEPAKPFGYGCKNIGFNGDMGEVEYLLLNINPLSIAESSAVSAYIEAVRELACELLDLMAEGLRVPDRSVFSRLIRDVDSDSLIRLNHYPPMPLLCKDEDSSPCNQNKVGFGEHSDPQILTILRSNDVGGLQISLNDGAWVPVTPDPATFWVNVGDLLQAMTNGRFVSVRHKALTNSSKSRMSMAYFAGPPPNARITVPPEMITPTKPALYKPFTWAEFKKAAYAMRLGDRRLGLFRMEGDEQVA

>Potri.010G149700.1 PtGA2ox6

MVVASPTQIHGEKLLAIELPVIDLSGERSMVSSLIVKACEEYGFFKVKNHGVPHDIIAKMENESFNFFAKTFDEKQKAGLDNSFGYGCKNIGFNGDTGEVEYLLCNTNPLSIAERSKTISNDPTEFRQQVYGYIEAVRELACELLDLMAEGLWVPDRSVFSRLIRDDDSDSIIRLNHYPAMPILCKDKDSSSPCNHNKVGFGEHSDPQILTILRSNDVGGLQISLNDGAWVPVTPDPTAFCVNVGDLLQAMTNGRFVSVRHKALTNSYKSRMSMAYFAAPPLNARIAVPPEMVTPIKPALYRPFSWAEFKNAAFALRLGDSRLGLFMLEVDDQVA

>Potri.014G117300.1 PtGA2ox7

MVVPSPTPIRTKTTKALGIPTVDLSLDNSSVSQLIVRACEEYGFFKVINHGVNKEVVTRLEEEAARFFGKPAAEKQQAGPASPFGYGCKNIGCHGDTGELEYLLLHTNLLSVSERSKTISNDPSGFSCAVSDYIRAVRQLACEILDLAAEGLWVPDKHVFSRLIRDVHSDSVLRLNHYPAVEEIADWDPSPIRIGFGEHSDPQILTILRSNDVAGLQICLHDGLWVPVPPDSTGFYVIVGDSFQVLTNGRFESVRHRVLTNSSQPRMSMMYFGAPPLTAWIAPLSHMVSQQNPSLYKPFTWSEFKKAAYSLRLRDTRLDLFKIHATEKSASL

>Potri.001G418200.1 PtGA2ox8

MDPPFQEKYRSLFNDYTIVSKDKDDSLMNANDECELPLIDLHRLTLEYSEREQCVKEIKQAASEWGFLQVVNHGIPQEMLKSLQYEQRKAFQHPFRKKAEDNILNLSANSYRWGNPRATCLRQLAWSEAFHVPLTDISRIGDAYKSLSASIEAFTTTANALAKGVAEILAENLGVSSTFFEENCPEETSYLRMNRYPPCPFSSEVFGLIPHTDSSFLTVLNQDQIGGLQLLKNGRWINVKPNPEALVINIGDLFQALSNDVYKSIKHRVLAPQQVERFSLAFFYCPTYETVIESSIKPSKYKEFTFREFMMQIQRDLKATGDKVGVSRFLL

>Potri.004G022800.1 PtGA2ox9

MVESSNFLQIINQAQRMDVDPPFQLTYKTLLEKTTEGATDHKDVVDIIEECELPLIDLGRLNLKNLEKEKCKSEIARASREWGFFQVVNHGISREILDKMRSEQVKVFKQPFNEKSKEEKFLNFSRGTYRWGTPTATCLKQLSWSEAFHIPMSEIQVSNGFSTALSSTMEQFATTVANLAQKLAEILAEKFGCKSDFIKENCLSSTCYLRMNRYPPCPIPSEVFGLMPHTDSDFLTILHQDEVGGLQLVKDGKWFAVKPNPEALIINIGDLFQAWSNDVYKSVEHRVVTNPRVERFSTAYFFCPSYDTVIQSCYEPSVYRKFSFKEYRQQVQEDVQKLGHKIGLPRFLV

>Potri.008G145300.1 PtGA2ox10

MHDLTSQPVSMIDSSPPLLRHYGEISRLPPQIPTPERSDLPDGIVLMEEYCQLPLIDLSCLSSTNEKVRLACADAICRASSEWGFFQVVNHGISPELVRNMRREQVKLFQTPFDKKVTCGVLNNSYRWGTPTATCPKQFSWSEAFHIPISKVFEQACYGEFSSLREVMVEFAAAMSKLARLLAGVLAENLGHPRGVFESTCQESNCFLRLNRYPACPISSEISGLVPHTDSDYLTILSQDEVGGLQLMKDSKWVAVKPNPDALIVNIGDLSQAWSNDIYKSVEHRVTANREKERYSIAYFLCPSYDSLIGSCRETSSIYRKFTFGEYRNQVQEDVKRTGRKIGLPRFLL

>Potri.010G096800.1 PtGA2ox11

MHDLTSQPIMMIDSSPPLLHHYGELTRLPPQIPTLECNDLPSGAVVMKEYCQLPLIDLSCLNSTIERERLACAEAICRASSEWGFFQVVNHGISPELVRNMRREQVELFQTPFDKKATCGVLNNSYRWGTPTATCPRQFSWSEAFHIPLSRVSEQACYGEFSSLREVMMEFAAAMSKLARVLAGVLAENLGHPRGVFENICQEINCFLRLNRYPACPISSEIFGLVPHTDSDFLTILSQDEVGGLQLMKDSKWVAVNPNQDALIVNIGDLFQAWSNDVYKSVEHKVVANGKMERYSIAYFLCPSYDSLIGSCMEPSIYREYRSQVQEDVKRTGRKIGLPRFLL

>Potri.011G026700.1 PtGA2ox12

MAMDPPFLETYKTLLDKATEGAHGHKEVVIIEECELPLIDLGRLNLGKLEKEKCKSDIARESQEWGFFQVVNHGISREILEKMMSEQVKVYRQPFNNKSKELFNFSSGTYRWGTPTATCREQLAWSEAFHIPMNDIPFSNGFSSLSSTMEQFATTVADLAQKLAAILAEKLGFKSNFFQENCLSSTCYLRMNRYPPCPIPSDVFGLMPHTDSDFLTILYQDEVGGLQLVKDGKWFAVKPNPEALIVNIGDLFQAWSNDVYKSVQHRVVTNPRVERFSTAYFFCPSYDTEIQSCYEPSVYKKFSFRMYRQQVQDDVKKLGRKVGLPRFLV

>Potri.011G134000.1 PtGA2ox13

MDPPFEEKYKSLLTNTTLLSKDKDYVIMSDYEEHELPIIDLHRLTLSFSEREQCVKEIRQAAREWGFFQVVNHGIPQEILEGIQLEQRKAFHHPFSKKAEENILNLPGYTWGNPAATCLRQLSWLEVFHIPLTDISKISGEYKSLRESIEAYTATAEKLAKDLSEILAENLGVSSTFFQENCLPETSYLRMNRYPPCPFSSEVLGALPHTDSCFVNVLNQDQIGGLQLWMNGKWISVKPNPEALIINIGDLFQVSSNDVYKSIRHRVLASKQAERFSLAYLYCPRKDAVIESGMKPSMYRKFTFGELTEQNARDVKETGNKGGIPRFLM

>Potri.011G134100.1 PtGA2ox14

MDPPFEEKYKSLLANATLLSKDKDDVIMSDYEEYELPIIDLHRLTLSFSEREQCIKEIRQAASEWGFFQVVNHGIPQEILERIQLEQRKLFHHPFSKKAEENILNLSENNGYRWGNHTATCLRQISWSEAFHIPLTDISKIGGEYKSLRESIEAYAASAEKLAKEMTEILAKNLDISSTYFQENFLPETNYLRMNRYPPCPFYSEVFGILPHTDSCFVNVLIQDQIGGLQLRVNGEWISVKPHPEALLINLGDLFQALSNDVYKSIRHRVVLASKQVERLSLAYLYCPRNDAVIQSGMKPSIYRKFTFEELMKQNSRDIEETGRKLGISRFLM

>Sobic.003G022700.1.p SbGA2ox1

MVVLAKGELEQIALPAAEPPPADVRSVDLSAPAGPAREAAARALVAACEEHGFFRVTGHGVPPELVRAAEAAAAGFFAQPQDEKDEEAPTLGYGSKRIGGNGDLGWVEYLLLGVTPAGAAVPAASASSSTLPCAAAAAAATASSPSAPAGSAGLLRDLLDEYTVAVRRMACAVLELMAEGLGIAGGAGDGDVLARLVTRADSDCMLRVNHYPPRPALNPCSLTGFGEHTDPQIISVLRANGTSGLEIALRDGAWASVPPDGDGFFVNVGDTLQVLTNGRFRSVRHRVVVNSEKSRVSMVFFGGPPPGERLAPLPQLLGDGGRS

>Sobic.003G300800.1.p SbGA2ox2

MVVLANPPVVDQIPLLRSPGPRDTFSGVPVVDLSSPAAARAIVDACERFGFFKVVNHGVPAATMGRAESEAVRFFAQAQADKDRAGPAYPFGYGSKRIGLNGDMGWLEYLLLAVDSASLSDACPVPSTAAFRSALNEYVAAVRKVAVRVLEAMAEGLGIADAAALSSMVTGAGGDQVFRVNHYPPCPALQGLGCSATGFGEHTDPQLISVLRSNGTSGLQIALRDGAQWVSVPSDRDAFFVNVGDSLQVLTNGRFKSVKHRVVTNSLKSRVSFIYFAGPALEQRIVPLPELLAEGEESLYKEFTWGEYKKAAYKTRLGDNRLAQFEKRSI

>Sobic.009G077500.1.p SbGA2ox3

MVVLTKGELEQITLPAVQRAAPPLAFVPEVDLSAAAASVAARSAAARAVAKACEDHGFFKVTGHGVPAPLLARLEAAAAAFFALPQRDKDKAAAAAVGGSPPFGYASKRIGGNGDLGWVEYLLLGVTPAGAAAAPSALSAAPVSSASEGAAPSCCFRDVLDEYIAAVRRMTCTVLELMAQGLGLDDDTAVFSRLVLDRDSDSMLRVNHYPPRPETAAAAGEPAEVRRPRLTGFGEHTDPQIISVLRSNDTAGLEISLRDGSWVSVPSDTQSFFVNVGDALQVLTNGRFRSVRHRVMVSSGRPRVSVIFFGGPPPRERLAPLPGLVDREGGRRRYREFTWREYKTSAYRTKLADNRLCYFETTTAAAAATS

>Sobic.009G196300.1.p SbGA2ox4

MVVLAKPPPALDQISLLRCPQPGDAASFFGVPAVDLSSPGAALAVVDACERFGFFKVVNHGVPTGVVDRLEAEAVRFFASPQADKDACGPANPLGYGNKRIGRNGDMGWLEYLLLALDGASSVSKASPVPSSSLRDAVNQYVAAVRGLATSVLEAVAEGLGVAPRDALSGMVTDAASDQVFRINHYPACPLLQRLPDSCGVTGFGEHTDPQLVSVLRSNGTAGLQVALHDDGGRWVPVPPDRDAFFVIVGDSLQVLTNGRLKSVRHRVVANSLKPRVSMIYFAGPAPAQRIAPLPQVLGHGEQSLYRDFTWGDYKKAAYRSRLGDNRLDPFRIQ

>Sobic.009G230800.1.p SbGA2ox5

MVAITAPSSIEQIPLVQCPRANASAAIPCVDLSAPGAAAAVADACRGVGFFRATNHGVPARVVEALEARAMAFFALPAQEKLDMSGAARPMGYGSKRIGSNGDVGWLEYLLLSVSANTVKISSLPPSLRAALEEYTAAVREVCGRVLELIAEGLGVDRSLLRAMVVGREGSDELVRVNHYPPCPLLPPVDCGVTGFGEHTDPQIISVLRSNSTAGLQIKLRDGRWVPVPPAPESFFVNVGDALQVLTNGRFKSVKHRVVAPEGAQSRLSVIYFGGPAPSQRIAPLPEVMRDGEQSLYREFTWAEYKTAMYKTRLADHRLGPFELRATNTNSCVPPPPPPSVDPYCNGSGICMPQPPPQQQQVAEVH

>Sobic.003G154100.1.p SbGA2ox6

MVVPSAAGREMAPESLPLGIIPTVDMSAPCGELARRLVRACAERGFFKAVNHGVPPRVSARMDAAASAFFARPGQAKQAAGPPDPLGYGSRNIGANGDVGELEYLILHADPGAVARKAKVIDKDDPSRFSVAVNEYVGAVRHLACRVLDLLGEGLGLRDPTSLSRLISAVDSDSLLRINHYPTSRSSAADISTKGIGFGEHTDPQILSLLRANDVDGLQVLLPDGHGGGDEQWVQVPADPSAFFINVGDLLQALTNGRLVSIRHRVMASTTRPRLSTIYFAAPPLDARVAALPETVTAGAPRRYRTFTWAEYKKAMYALRLSHNRLDLFHATSGSSRDVIDDDHEQ

>Sobic.009G053700.3.p SbGA2ox7

MVVPSTTPVVVRQETPPPPLPLPPSHDGIIGIPTVDMSAPGGRGALSRQVARACAEHGFFRAVNHGVAVAPAAGPAARLDAAARTFFALPPHDKQRAGPPSPLGYGCRTIGFNGDAGELEYLLLHANPAAVAHRARSIDTDDPSRFSNVVNEYVGAMRQLACEILDLLGEGLGLKDPRSFSKLITDTDSDSLLRINHYPPACTIQKLDHDNQCKMKSSFRIKTGNGVNQSAGARIGFGEHSDPQILSLLRSNDVDGLQVLLNCDGREVWVQVPSDPSAFFVNVGDLLQALTNGKVISVRHRVIANSCRARLSTIYFAAPPLHARILALPETITANSPRQYRPFTWAEYKKTMYSLRLSHSRLNLFHIDHDDHSNVGKGEQE

>Sobic.002G003100.1.p SbGA2ox8

MEDYDYEPPLMATYRHLLDSHPHRLDVVDHRSGADDDEEGFLLPVIDLSSLLEQSSSGAEAAAEQCRASIVRAASEWGFFQVTNHGVPQALLDELHQAQVAVFRRPFHLKASQPLLDFSPESYRWGTPTATCLDQLSWSEAYHIPTTNTTAAADDKTRLVVEEVSTAMSKLAQRLAGILVADLLLGDSSIGDGEDDDTAAAVVSRCTRSTCFLRLNRYPPCPAPSGAYGLCPHTDSDFLTILHQDGVGGLQLVKAGRWVAVKPNPGALIVNVGDLLQAWSNDRYRSVEHRVMASDARERFSVAFFLCPSYDTLVRPRCGAGGPPRYESFTFGEYRNQIREDVRLTGRKLGLQRFRKPE

>Sobic.004G222500.1.p SbGA2ox9

MPAFAGSAAEPPLADSYYALLRRRGGGGNNDDEEASGGAYYYDTTSSTVPSSDDDVAECELPMIDVGCLTTLTRSEHGSSSEAERAACTAAIARAAEEWGFFQVRNHGVSQELLDEMRREQARLFRLPFEAKATAGLLNHSYRWGTPTATSPQQLSWSEAFHVPLAGVSGSAAAGTCDFGDLTTLRDVTREVAGAMSKLAGTLARVLAEEARPRPCLPAGGGGGGGGERFPEGCDETTCFLRLNRYPPCPIAADAFGLVPHTDSDFLTVLCQDQQVGGLQLMKGGRWVAVKPIPGALIVNIGDLFQAWSNNRYKSVEHKVMTNAKTERYSVAYFLCPSYDSPIGTCEEPSLYRTFTFGEYRRKVQEDVKRTGKKVGLPNFLV

>Sobic.006G150800.1.p SbGA2ox10

MRYVAATPTMPALTTTTAESAAEPPLADSYLDLLRRGGIATPPRTEGRCCVQERELPLIDLSCLQQRSGGGGGSARAACADAMARAASEWGFFQVTGHGVSRALLERLRAEQARLFRLPFETKAKAGLLNGSYRWGAPTAATSLRHLSWSEAFHVPLASISGAACDFGDLSSLRGVMQEVADAMSRVAKTVAVALAGSLLQGGGHDEHEAAAAFPAGCDETTCYLRLNRYPACPFAADTFGLVPHTDSDFLTVLCQDHVGGLQLLKDGRWVAVKPRPDALIVNIGDLFQAWSNNRYKSVEHKVVANATAERFSAAYFLCPSYDSPVGTCGEPSPYRDFTFGEYRRKVQEDVKRTGRKIGLPNFLKQQSRQ

>GSVIVT01000687001 VvGA2ox1

MGVFSKPAIEQLPLIRNCMPFSGIPLIDLSQPDSKALLIEACQEFGFFKVINHGVPMDLISKLEAEAINFFSLPLSEKEKAGPPNPSGYGNKRIGSSGDIGRVEYLLLNPQSFPSVFGQNPDMFRSAVSDYLSAVRKMACEILELLADGLMIQPRNVFSKLLMDEQSDSVFRLNHYPPYPERQALSGKCMIGFGEHTDPQIISVLRSNNTSGLQISLRNGNWISVPPDENSFFINVGDSLQVMTNGRFQSVKHRVLTNSCKSRVSMIYFGGPPLSEKIAPLPSLMEGEESHYKEFTWFEYKRSAYNTRLADNRLVFFQKVAAT

>GSVIVT01000689001 VvGA2ox2

MVVLSKPTIADFPPIINCKSTTLFPVIPTVDLSEPDSKHLVVKSCEEFGFFKVINHGIPLELISRLETEVIEFFSLSLSEKQKAGPPDPFGYGNRSIGPNGDVGWVEYLLLTMNQERNSQKLATIFGKYPEKLCSALNDYVLAVKKMACELLELMADGLRIKPRNVFSKLLMDEQSDSVFRLNHYPPYSELQASNGKNMIGFGEHTDPQIISVLRSNNTSGLQISLGNGSWISVPPDANSFFINVGDSLQVMTNGRFKSVRHRVLANSIKSRISMIYFGGPPLNEKIAPLPSLVEGKESLYKEFTWFEYKRSAYKSRLGDNRLSQFERTAAT

>GSVIVT01028169001 VvGA2ox3

MVVPSPSPIRSKKTKAVGIPVIDLSLNRSAIAELIVNACEDYGFFKVVNHGVPKEIIGRLEEEGLSFFAKPSSEKQKAGPASPFGYGCKNIGFNGDRGELEYLLLHTNPVSISERSKAISNDPTEFSCAVTDYIQGVRELCCEILDLIGEGLWLQDKMVFSRMIRDVHSDSVIRVNHYPAVKDVKEWDPCDPIGFGEHSDPQILTILRSNDVPGLQIRLRDGLWVPVPPDPTEFCVFVGDALEAMTNGRLMSVRHRALTSSVKARLSMMYFGAPPLNAWISPLPDMVSPQKPSLYRPFSWVEYKKAAYSLRLGDRRLDLFKINTISTTLVTRTI

>GSVIVT01034945001 VvGA2ox4

MVVASPTPIGSEKLIAVEVPIIDLAARGPKVAELIVKASQEFGFFKVINHGVPEDVIRKMEEESFNFFGKPDSEKKKAGPAQPFGYGCKNIGFNGDMGEVEYLLFNTNPHCISQRSETISNDPTKFSSAVSGYIQAVTDLACEILDLMAEGLWVQNTSVFSSLITHLDSDSVFRLNHYPPPLKDRDTSPSSSFQIHHQGNNIGFGEHSDPQILTILRSNDVGGLQICLGDGVWVPVPPDPTSFCVNVGDLLQAMTNGRFVSVRHRALTNSDKPRMSMAFFGAPPLHALITSPPEVVTPERPSLYRPFTWAEYKEITYSLRLGDSRLNLFKACPDKEAQE

>GSVIVT01001966001 VvGA2ox5

MESEPPFGEFVNSVFGNIVEQEETEAKFDVDECELPLIDLGHLNLGNLEHEECKRKICEASTEWGFFQIVNHGVSKEILSRIHREQVELFRQPFQIKTNEKLLNLSSGCYRWGTQTAITQKQFAWSEAFHIPLSTIFQLSEFEGLRSSLQEFAIKASDLAQQIAKILAENLGCKSTFFFKNCLPSSCYIRMNRYPACPVSSKVFGLIPHTDSDFLTVLHQDQVGGLQLLKDGKWIRVKPNPDALVINIGDLFQAWSNGVYKSLEHRVVANHEIERFSFAYFLCPSHDTVIQSCCEPSIYRKFSFREYRQQVEEDVKTTGDKVGLSRFRR

>GSVIVT01010228001 VvGA2ox6

MRASNIKHQKWVGRGAVCTVSGRVVEKPENEKPTFSSQLLKTMDVHFRRTMTNSNPPLLQHYGVLCQHGETPAVQQRDCGNNGAAMEECQLPLIDLEGLWSESEEERLACASAIGRASSKWGFFQVVNHGIRPELLSEMRREQVKLFETPFERKAACQLLDNSYRWGTPTATCPKELSWSEAFHIPLTKVSEEACYGEFCSLREVMQEFAKAMSNLARLLAGVLAESLGHQKGVFDEICDENTCFLRLNRYPPCPVSPEVFGLVPHTDSDFLTILYQDEVGGLQLMKDSKWVAVKPNKDTLIVNIGDLFQAWSNNEYKSVEHQVMANAIKERYSIAYFLCPSYDAFIGSCSEPTIYRKFTFGEYRQQVQEDVKKTGHKVGLPRFLLQTTQ

>GSVIVT01012628001 VvGA2ox7

MPFLQYKYHTPHPTFLLEHSSPLSLNPTPFSSGFSAHSLSFRHFTYRYFHILFPTKLSVEPLAQHLQVVVHWCLKEEMVESDTKLPFSMDSDPPFEETYKTLFENSIEESKINRANQILITCEECELPLIDIGRLSMGELEREECKKEIARASQEWGFFQVINHGVSSEILEDMRSKQMQVFKQPFRLKTNHQYLNLSAGCYRWGTPTATCLSQLSWSEAFHIPLMDISSSGGLPTTLSSTMGQFAATVSDLAQRLVEILAEEMGHKSTFFKEKCLPSTCYIRMNRYPPCPTSQIFGLMPHTDSDFLTILHQDQVGGLQLVKDGRWIAVKPNPEALIINIGDLFQAWSNGVYKSVQHRVVTNQKVERFSTAYFLCPSYDAVIESCVEPLLYRKFSFREFRQQVQEDVQKLGYKVGLPRFLV

>GRMZM2G022679_P01 ZmGA2ox1

MVVLAKPPVVDQIPLLRSPGPRDSFSGVPVVDLSSHGAARAIVDACERFGFFKVVNHGVAAATMDRAESEAVRFFAQAQADKDRAGPAYPFGYGSKRIGLNGDMGWLEYLLLAVDAASLSDACPVPSSAAFRSALNEYVAAVRKVAARVLEAMAEGLGIADADALSSMVSGAGSDQVFRVNHYPPCPALQGLGCSTTGFGEHTDPQIISVLRSNGTSGLQIALRDGAQWVSVPSDRDAFFVNVGDSLQVLTNGRFRSVKHRVVTNSLKSRVSFIYFAGPPLGQRIAPLPQVLAEGEESLYKEFTWGEYKKAAYKTRLGDNRLAQFEKRSNI

>GRMZM2G031432_P01 ZmGA2ox2

MVVLAKGELEQIALPAAAPPRADVRSVDLSAPAGPAREAAARALVAACEEHGFFRVTGHGVPAELVRAAEAAAAGFFARPQGEKDGEAPTLGYGSKRIGGNGDLGWVEYLLLGVNPAVPAASASSSTLPLRGLLDEYTVAVRRMACAVLELMAEGLGIAGAGDGDGDTVLARLVARADSDCMLRVNHYPPRPALNYPCLTGFGEHTDPQIISVLRANGTSGLEVALRDGAWASVPPDGDAFFVNVGDTLQVLTNGRFRSVRHRVVVNSEKSRVSMIFFGGPPPDERLAPLPQLLGDDGGRSRYRDFTWSEFKTSGCRTRLAEDRLSRFEKQ

>GRMZM2G031724_P01 ZmGA2ox3

MVVVLANPPVVDQIPLLRSPGPRDSFSCVPVVDLSGPGAARAIVDACERFGFFKVVNHGVPAATMDVAESEAVGFFAQAQADKDRAGPRASYPFGYGSKRIGLNGDMGWLEYLLLAVDSASLSDACPVPSGAAFRSALNEYVAAVRDVAARVLEAMAEGLGIADAAALSSMVTGAGSDQVFRVNHYPPCPALQGLGCTATATGFGEHTDPQIISVLRSNGTSGLQVALRDAAQAQQWVSVPSDRDAFFVNVGDSLQVLTNGRFESVKHRVVTNSLKSRVSFIYFAGPALEQRIAPLAQLLAEGEESLYREFTWGEYKTAAYKTRLGDNRLAQFQRCSI

>GRMZM2G051619_P01 ZmGA2ox4

MVVLTKGELEQIALPAVQRASPPPPAAVPEVDLAAGDAAAAARAVAKACEDHGFFKVTGHGVPPHLLARLEAAAAAFFALPQREKDRAAGCPFGYASKRIGANGDLGWVEYLLLAVTAAGAAAAPGSACEGAEPSCFRAVLDEYVAAVRRMTCTVLQLMAQGLGLDDRDVFSRLVLDRDSDSMLRVNHYPPAAETRRLTGFGEHTDPQIISVLRSNDASGLEITLRDGTWVSVPSDTESF

FVNVGDALQVLTNGRFRSVRHRVMVSSARPRVSVIFFGGPPPRERLAPLPGLVDREGGRRRYREFTWREYKNSAYRTKLADNRLCSFETMATS

>GRMZM2G152354_P01 ZmGA2ox5

MVVLAKGELEQIALPAAEPPPADVRSVDLSAPAGPAREAAARALVAACEEHGFFRVTGHGVPARLVRAAEAAAAGFFARPQGEKEEEGPTLGYGSKRIGGNGDLGWVEYLLLGGTPAAAPSSALLPCAAAPSPSAPAGPLRDLLDEYTVAVRRMACAVLELMAEGLGIAGGAGDAVLARLVARADSDCMLRVNHYPPRPALNPSLTGFGEHTDPQIISVLRANGTSGLEIALRDGAWASVPPDGDAFFVNVGDTLQVLTNGRFRSVRHRVVVNSEKSRVSMVFFGGPAARREAGPAAAAPGRRRPEPVPGLHLERVQDQRVQDQARGRPPVPLREEVARGYVICMTGGRIDYHVCFSVYVSCIATALATGARTCILVAMLVLYVHVYARIYMVAPHRVSCIQCYLLSMEYQPCMHGPVISFYNYNYPPVLPDSPGVVRVRVSVNQSAVQQRRHDDVLRTATNDAGTGRDPSRTIYQD

>GRMZM2G155686_P01 ZmGA2ox6

MVAITAPSSIEQIPLMRCPRANAVQQAGAAVPCVDLSAPGAGAAVADACRSVGFFRATNHGVPARVADALEARAMAFFALPAQEKLDMSGAARPLGYGSKSIGANGDVGWLEYLLLSVSANTVKISSLPPSLRAALEEYTAALREVCGRGAGADSGGAGRGAVPAARDGGGAGRQRRGGAGEPLPALPAAAAGGLRRDGVRGAHGPADHLRALVQPHCGPADQAPGRQVGPRAPHTGIPLRQRRRLAAGSDERAVKEREAPGGGAGAGAGWRAVPAVRDLLRRAGAVAADRAAAAGDAGRGAEPVPGVHVGRVQARHVQDPPRRPPPRALRAARQHQRRQQARRARGGQRGPGALQRRQRLRHAAAAAGGAGALDYAGFGLDMVSSAKASSPPRLSCCCSLVHAPWSRSAPGSLSRSSDPSLLFNPYRVTEM

>GRMZM2G427618_P01 ZmGA2ox7

MVVLAKPPALDQISLLRSPQPGDASSFPGVPAVDLSSPGAALAVVDACERFGFFKVVNHGVPAGVVDRLEAEAVRFFASPQAAKDACGCGPASPLGYGNRRIGRNGDMGWLEYLLLALDGNASVSKASPVPSSSLRDAVNQYVASVRGLATSVLEAVAEGLGVAPRDALSGMVADAASDQVFRINHYPACPLLQRLPDSCGVTGFGEHTDPQLVSVLRSNGTPGLQLALHGDDGRWVPVPPDRDAFFVIVGDSLQVLTNGRLKSVRHRVVANSLKPRVSMIYFAGPAPAQRIAPLPQLLGHGKQSLYRDFTWGDYKKAAYRSRLGDNRLDPFRI

>AC215639.3_FGP002 ZmGA2ox8

MVVPSTTPVVRQQTTPPQSSHAGGIPTVDLSAHGGRGALSRQVVRACAEHGFFRAVNHGVPPGPAARLDAAARTFFALAPRDKQRAGPPSPLGYGCRSIGFNGDAGELEYLLLHANNPAAVAHRARAIDAEEPSRFSNVVNEYVGAMRQLACEILDLLGEGLGLEDPRSFSKLITDTDSDSLLRINHYPTACNAHNLDHDSQCKMKSSVRTKTTSNGVKPSAGGRVGFGEHSDPQILSLLRANDVDGLQVLLNADGKEVWVQVPADQSAFFVNVGDLLQALTNGKLVSVRHRVIASSSRARLSTIYFAAPPLHARILALAETITANAPSQYRPFTWAEYKKTMYSLRLSHSRLNLFHIDHDGHSNVGEGEE

>GRMZM2G078798_P03 ZmGA2ox9

MVVPSTTPVVRQETPPPSHDGIGIPTVDLSAPGGRGALSRQVARACAQHGFFRAVNHGVAPGPAARLDAAARTFFALAPHNKQRAGPPSPLGYGCRSIGFNGDAGELEYLLLHANPAAVAHRARSIDTDDPSRFSNVVNEYVGAMRQLACEILDLLGEGLGLKDPRSFSRLIADTDSDSLLRINHYPPPCAIHKLDHDSQCRMKNSFRIVAGNGANQSAGARIGFGEHSDPQILSLLRSNDVDGLQVLLNSDGREVWVQVPADPSAFFVNVGDLLQAFTNGKVISVRHRVIASSSRARLSTIYFAAPPLHARILALPETVTANSPRQYRPFTWAEYKKTMYSLRLSHSRLNLFHIGHDDHGKGEQE

>GRMZM2G176963_P01 ZmGA2ox10

MAPESLPLGIIPTVDMSAPSGRGELARRLVRACAERGFFKAVNHGVPPRVSARLDAATSAFFARPAPVKQAAGPPDPLGYGSRNIGANGDVGELEYLILHAQPAAVARKAMVIDAEDPSRFRKHADRSFQAIYIRTLQTGRRTNGSFTMQPYLARPNPLPKKLYLVLVVPCSSIWVVHALIGLMLVRIYNRFIVTEINNMSGNLCVAVNEYVDAVRRLACRVLDLLGEGLGLGDPTSLSRFVSAVVDSDSVLRINHYPTSSAAADVSAKGIGFGEHTDPQILSLLRANDVDGLQVLLPDARGGSGGDQWVQVPADPSAFFINVGDALQALTNGRLVSIRHRVMASTTRPRLSTIYFAAPPLDARIAALPETVVAGAARRYRTFTWAEYKKAMYALRLSHNRLDLFHADASSGGSGDKKHDI

>GRMZM2G006964_P01 ZmGA2ox11

MRYVAATPTMPSLVAESAAEPPLVDSYLELLRRGGGGGGIAAATEGCVQERELPLIDLTCLQGSAGEAARTTCADAMARAASEWGFFQVTGHGVSRALLERLRAEQARLFRLPFETKAKAGLLNGSYRWGAPTATSLRHLSWSEAFHVPLASISGTACDFGELSSLRDVVQEVADAMSRVAKTVAVALAGSLLGHDEAAAFPAGCGETTCYLRLNRYPACPFAANTFGLVPHTDSDFLTVLSQDQVGGLQLMTDAGWVAVKPRPDALIVNIGDLFQAWSNNLYKSVEHKVVANAAAERFSAAYFLCPSYDSLVGTCGEPSPYRDFTFGEYRRKVQEDVKRTGRKIGLPNFLKHRPPPQSRPA

>GRMZM2G153359_P01 ZmGA2ox12

MPAFAGGAAEPPLADSYYALLRRGNDDEGAYTTSTAPWDDVSLPVAECELPMIDVGCLTTADDGSSPEAEAERAACAAAIARAAEEWGFFQVRNHGVPQELLEEMRREQARLFRLPFETKATAGLLNDSYRWGTPTATSPRQLSWSEAFHVPLAGVSGSGTTCDFGDLTTLRDVTREVAGAMSKLAGTLARVLAEALLGRRPAGERFPEGCDETTCFLRLNRYPACPISPGALGLVPHTDSDFLTVLCQDQQVGGLQLMKGDSWVAVKPIPGTLVVNIGDLFQAWSNNRYKSVEHKVMTNARTERYSVAYFLCPSYDSPIGTCEEPSLYRTFTFGEYRRKVQEDVKRTGKKVGLPNFLAQT

>GRMZM2G177104_P01 ZmGA2ox13

MQQSSAASMAMEEDDDYEPPLMATYKHLLDSHPHRQLGAGAAQDDEDCFLLPVIDLSSLLPPVHHRKQSSSAAAAEQCRASIVRAASEWGFFQVTNHGVPQVLLDELHQAQAGVFRRPFQLKAHQPLLDFSPESYRWGTPTATCLEQLSWSEAYHIPTTTTTTGNDDKTRLVVEEVSTAMSKLAQRLAGILVAELGEDSAAAVVSRCTRSTCFLRLNRYPPCAAAASGVVYGLCPHSDSDFLTILHQDGVGGLQLVKDGQWVAVKPSPGALIVNVGDLLQAWSNDRYRSVEHRVMASATRERFSAAFFLCPSYDTLIRPRCGAGGPPPRYESFTFGEYRNQIREDVRLTGRKLGLQRFRKAE
